# Supplementary material for: Amplification is the primary mode of gene-by-sex interaction in complex human traits
Source: Cell Genom. 2023 Apr 6;3(5):100297. doi: 10.1016/j.xgen.2023.100297 (PMC10203050; doi:10.1016/j.xgen.2023.100297)
Supplement: Document S2. Article plus supplemental information [file mmc2.pdf]

Cell Genomics

Article

Amplification is the primary mode of gene-by-sex interaction in complex human traits

Graphical abstract

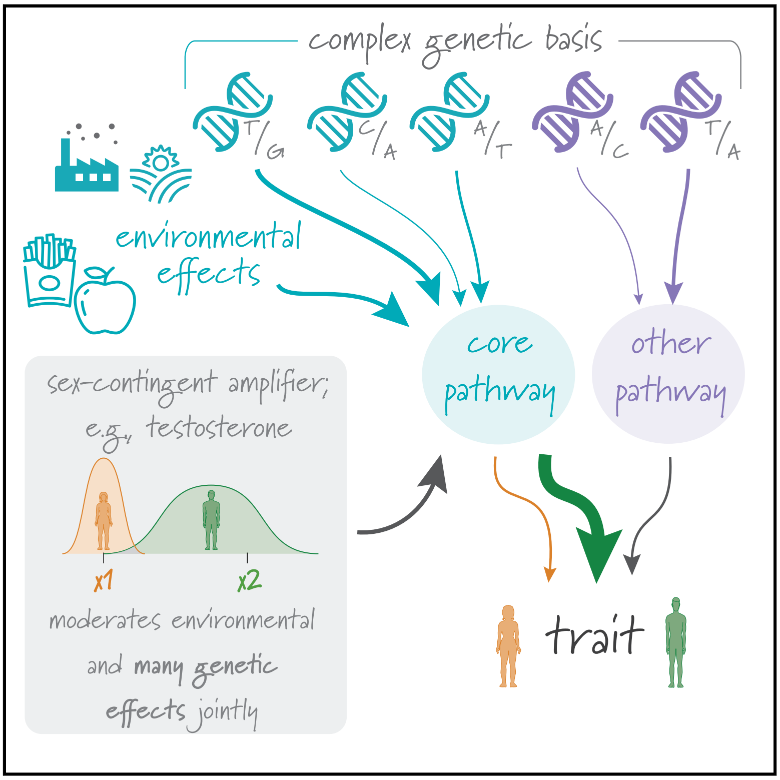

Authors

Carrie Zhu, Matthew J. Ming, Jared M. Cole, Michael D. Edge, Mark Kirkpatrick, Arbel Harpak

Correspondence

arbelharpak@utexas.edu

In brief

Zhu et al. estimate the ways in which genetic effects on anthropometric measurements and biomarkers depend on sex. They find pervasive sex contingency acting not through differences in genetic basis but largely through "amplification"—systematic differences in the magnitude of numerous genetic effects. They show that amplification explains trait variance differences between the sexes and that it may be consequential for sex-specific natural selection.

Highlights

- Genetic effects on physiology often depend on sex in an underappreciated way
- Amplification: differences in magnitude shared by many genetic effects
- Amplification may act jointly on co-moderated environmental and genetic effects
- Polygenic scores that incorporate amplification improve phenotypic prediction

Zhu et al., 2023, Cell Genomics 3, 100297
May 10, 2023 © 2023 The Author(s).
https://doi.org/10.1016/j.xgen.2023.100297

CellPress

Cell Genomics

CellPress
OPEN ACCESS

Article

Amplification is the primary mode of gene-by-sex interaction in complex human traits

Carrie Zhu,[1,2] Matthew J. Ming,[1,2] Jared M. Cole,[1,2] Michael D. Edge,[3] Mark Kirkpatrick,[2] and Arbel Harpak[1,2,4,*]
[1]Department of Population Health, The University of Texas at Austin, Austin, TX, USA
[2]Department of Integrative Biology, The University of Texas at Austin, Austin, TX, USA
[3]Department of Quantitative and Computational Biology, University of Southern California, Los Angeles, CA, USA
[4]Lead contact
*Correspondence: arbelharpak@utexas.edu
https://doi.org/10.1016/j.xgen.2023.100297

SUMMARY

Sex differences in complex traits are suspected to be in part due to widespread gene-by-sex interactions (GxSex), but empirical evidence has been elusive. Here, we infer the mixture of ways in which polygenic effects on physiological traits covary between males and females. We find that GxSex is pervasive but acts primarily through systematic sex differences in the magnitude of many genetic effects ("amplification") rather than in the identity of causal variants. Amplification patterns account for sex differences in trait variance. In some cases, testosterone may mediate amplification. Finally, we develop a population-genetic test linking GxSex to contemporary natural selection and find evidence of sexually antagonistic selection on variants affecting testosterone levels. Our results suggest that amplification of polygenic effects is a common mode of GxSex that may contribute to sex differences and fuel their evolution.

INTRODUCTION

Genetic effects can depend on context. If the distribution of contexts differs between groups of people, as it does for males and females, so should the average genetic effects on traits.[1,2] In particular, such gene-by-sex interaction (GxSex) may be a result of sex differences in bodily, environmental, and social contexts or epistatic interaction with sex chromosomes.[3–9] Sex differences in genetic effects on complex traits are clearly of high evolutionary[8,10–14] and translational[9,15–22] importance. However, with the exception of testosterone levels,[23–26] the basis of sexual dimorphism in complex traits is not well understood.[19] To date, empirical evidence of GxSex in genome-wide association study (GWAS) data—whether focused on identifying large GxSex effects at individual loci or by estimating genetic correlations between the sexes for polygenic traits—has been lacking.

Here, we set out to study governing principles of GxSex in complex human traits and explain why current approaches for characterizing GxSex may be lacking for this goal. We then suggest a mode of GxSex that may have gone largely underappreciated: a shared difference in the magnitude of effect of many variants between the sexes, which we refer to as "amplification."[27] Amplification can happen for a large set of variants regulating a specific pathway if the pathway responds to a sex-contingent cue.[28–31] In classic hypothesis-testing approaches that test for a GxSex effect separately in each variant, the signal of amplification may be crushed under the multiple-hypotheses burden. On the other hand, even state-of-the-art tools designed with complex traits in mind may miss amplification signals. They often treat genetic correlation (between GWAS estimates based on samples from two contexts, such as males and females) as a litmus test for whether effects are the same in the two contexts,[32–36] but correlations are scaleless and thus may entirely miss amplification signals.

We developed a new approach for flexibly estimating male-female genetic covariance relationships and applied it to 27 complex physiological traits in the UK Biobank. We found that amplification is pervasive across traits. The inferred polygenic covariance structure explains sex differences in trait variance remarkably well and, in most cases, helps improve phenotypic prediction. Finally, we consider an implication of polygenic GxSex for sexually antagonistic selection. We develop a model that demonstrates how variants that affect traits may be subject to sexually antagonistic selection when male and female trait optima are very different or, surprisingly, even when the trait optima are very similar. We developed a novel test for sexually antagonistic polygenic selection that connects GxSex to signals of contemporary viability selection. Using this test, we find subtle evidence of sexually antagonistic selection on variants affecting testosterone levels.

RESULTS

The limited scope of single-locus analysis

We conducted GWASs stratified by sex chromosome karyotype for 27 continuous physiological traits in the UK Biobank (UKB) using a sample of ∼150,000 individuals with two X chromosomes, another sample of ∼150,000 individuals with XY, and a combined sample that included the XX and XY samples. We chose to analyze traits with SNP heritabilities over 7.5% in the

This is an open access article under the CC BY license (http://creativecommons.org/licenses/by/4.0/).

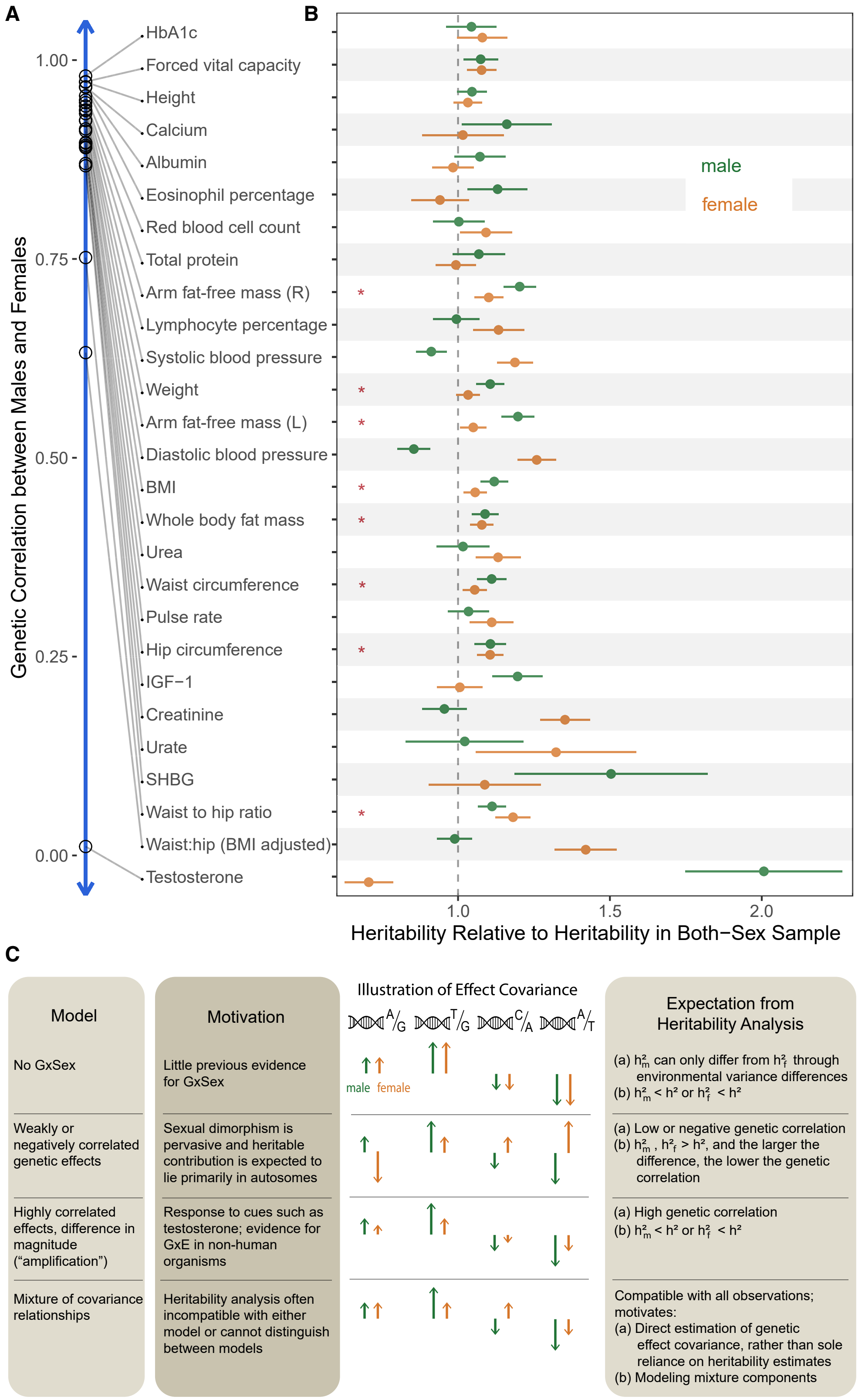

(legend on next page)

combined sample to have higher statistical power. While there is not a strict one-to-one relationship between sex chromosome karyotype and biological sex, we label XX individuals as females and XY individuals as males and view these labels as capturing group differences in distributions of contexts for autosomal effects rather than as a dichotomy.[9,22,37] Throughout, we analyze GWASs on the raw measurement units as provided by the UKB. (See the note on the rationale behind this choice under Flexible model of sex-specific genetic effects as arising from a mixture of covariance relationships).

Among the 27 traits, we observed substantial discordance between males and females in associations with the trait only for testosterone and waist:hip ratio (whether or not it is adjusted for BMI; Figure S1). For testosterone, as noted in previous analyses, associated genes are often in separate pathways in males and females.[23,25] This is reflected in the small overlap of genes neighboring top associations in our GWAS. For example, in females, the gene CYP3A7 is involved in hydroxylation of testosterone, resulting in its inactivation. In males, FKBP4 plays a role in the downstream signaling of testosterone in the hypothalamus. Both genes, to our knowledge, do not affect testosterone levels in the other sex.

For waist:hip ratio, we saw multiple associations in females only, such as variants near ADAMTS9, a gene involved in insulin sensitivity.[38] As a previous work established,[23,25,26] testosterone and waist:hip ratio are the exception, not the rule; most traits did not display many sex differences in top associations. For instance, arm fat-free mass, a highly heritable dimorphic trait, showed near-perfect concordance in significant loci (Figure S1). A previous study[26] examining the concordance in top associations between males and females found few uniquely associated SNPs (<20) across the 84 continuous traits they studied; waist:hip ratio was an exception with 100 associations unique to one sex. Considering the evidence of the polygenicity of additive genetic variation affecting many complex traits,[39–41] it stands to reason that looking beyond lead associations, through a polygenic prism, may aid characterization of non-additive effects (such as GxSex) as well.

The limited scope of analyzing GxSex via heritability differences and genetic correlations

We turned to consider the polygenic nature of GxSex, first by employing commonly used approaches: comparing sex-specific SNP heritabilities and examining genetic correlations. We used linkage disequilibrium score regression (LDSC)[36,42] to estimate these for each trait. In most traits (17 of 27), males and females had a genetic correlation greater than 0.9. Testosterone had the lowest genetic correlation of 0.01, which suggests very little sharing of signals between males and females (see similar results by Flynn et al.[25] and Sinnott-Armstrong et al.[23]).

For the majority of traits (18 of 27), male and female heritabilities were greater than the heritability in a sample that included both sexes. For instance, in arm fat-free mass (right), the heritability in the both-sex sample was 0.232 (±0.009), while the heritabilities for male and female were 0.279 (±0.012) and 0.255 (±0.011), respectively. In particular, all body mass-related traits, excluding BMI-adjusted waist:hip ratio, had greater sex-specific heritabilities (Figures 1A and 1B).

In addition, we noticed a trend where, as the genetic correlation decreased, the difference between the heritabilities within each sex and in the sample combining both sexes tended to become larger (Pearson $r = -0.88$, paired t test $p = 10^{-10}$; Figures 1A and 1B). Nonetheless, several traits with genetic correlation above 0.9 also present relatively large sex differences in heritability. For example, diastolic blood pressure and arm fat-free mass (left) had differences of 5.2% (two-sample t test $p = 3 \cdot 10^{-6}$) and 3.4% (two-sample t test $p = 0.04$), respectively. These examples are incompatible with a model of pervasive uncorrelated genetic effects driving sex-specific genetic contributions to variation in the trait (Figure 1C, second model).

We therefore considered two other alternative hypotheses under a simple additive model of variance in a trait. Differences in heritability are due to sex differences in genetic variance, in environmental variance, or both. If genetic effects are similar, then differences in environmental variance alone could cause heritability differences (Figure 1C, first model). But as we show in the STAR Methods, under such a model, the heritability in the combined sample cannot be smaller than both sex-specific heritabilities.

Therefore, the observation of higher sex-specific heritabilities for most traits suggests that the genetic variance must differ between males and females. Given the random segregation of autosomal alleles, independent of an individual's sex chromosome karyotype, and assuming, further, that there is little to no interaction of sex and genotype affecting participation in the UKB,[43] allele frequencies in males and females are expected to be very similar. Thus, this observation suggests that causal genetic effects differ between males and females for most traits analyzed.

A last hypothesis that might tie together many of the observations summarized in Figure 1 is a less appreciated mode of GxSex, amplification, where the identity and direction of effects are largely shared between sexes (leading to high genetic correlation), but the magnitude of genetic effects differs—e.g., larger genetic effects on blood pressure in females—which, in turn, leads to differences in genetic variance (Figure 1C, third model).

We can test the hypothesis that amplification acts systematically—across a large fraction of causal variants—by examining

Figure 1. Heritabilities and genetic correlations cannot fully distinguish models of GxSex
(A) Genetic correlations between males and females, estimated using bivariate LDSC, are shown in descending order.
(B) The x axis represents the relative heritability (i.e., the SNP heritability divided by the SNP heritability) estimated in the sample with both sexes combined. Red asterisks indicate body mass-related traits with greater heritability in both sex-specific samples compared with the sample combining both sexes. Error bars represent ± 1 SE.
(C) Polygenic models of GxSex. We examine different models of the nature of GxSex in complex traits that link to previous studies and motivations. Each model leads to different expectations from the analysis of heritability and genetic correlations (A and B). The illustrations in the third column depict examples of directions and magnitudes of genetic effects corresponding to each model. h_m^2, h_f^2, and h^2 denote narrow-sense heritabilities in males, females, and a combined sample, respectively.

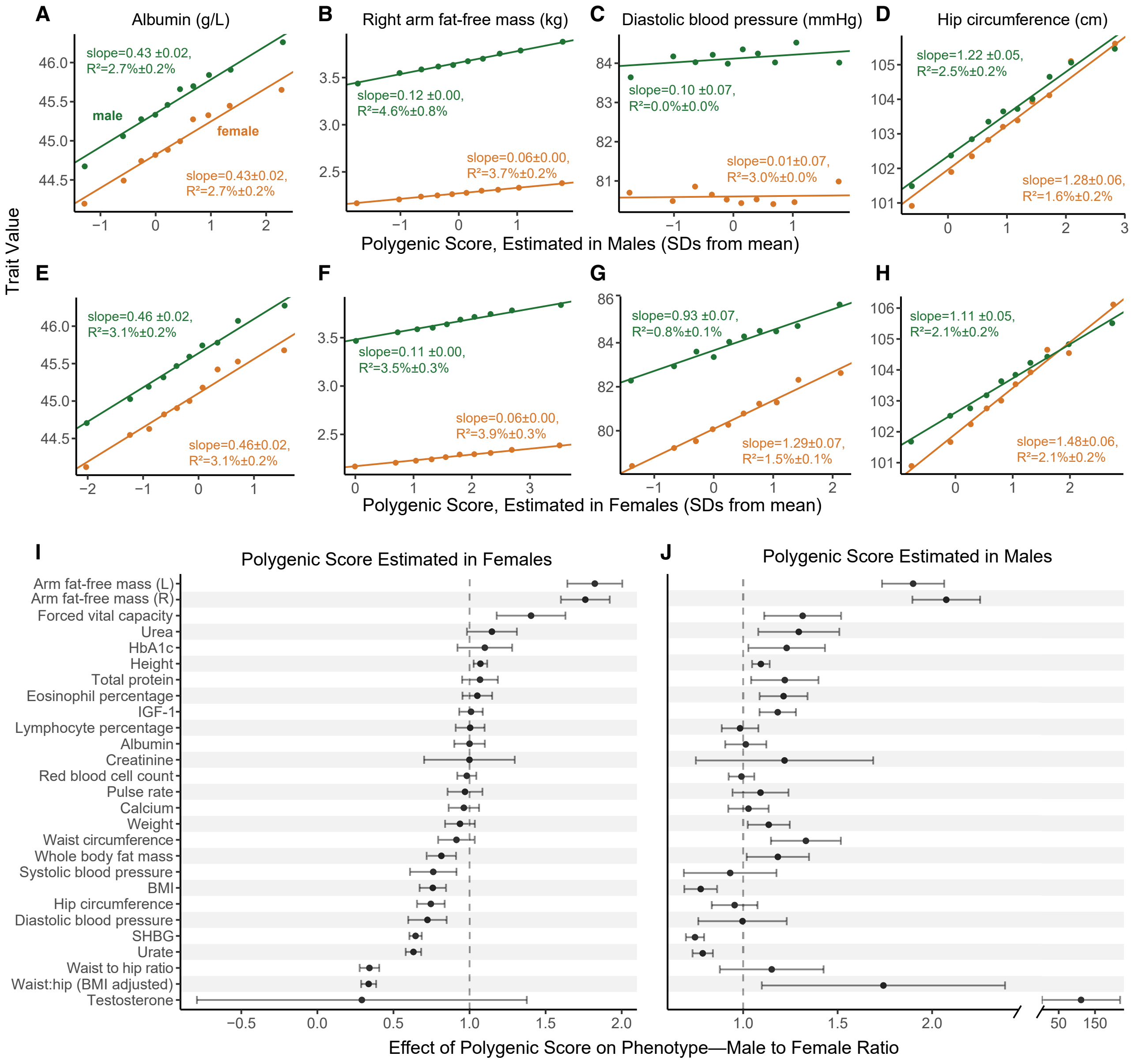

Figure 2. Evaluating evidence of systematic amplification

(A–D) We regressed trait values in males (green) and separately in females (orange) on a PGS estimated in an independent sample of males. Points show mean values in one decile of the PGS; the fitted line and associated effect estimate and R^2 correspond to regressions on the raw, non-binned data. In some traits, like albumin (A), the PGS has a similar effect on the trait in both sexes. In other traits (B and D), the estimated effect of the PGS differs significantly, consistent with a substantial difference in the magnitude of genetic effects of sites included in the PGS.

(E–H) Same analysis as in (A)–(D) but with a PGS pre-estimated in an independent sample of females.

(I and J) Summary of the ratio of the effect of the PGS on the trait (±2 SE) in males to the effect in females across physiological traits. See results for other traits in Figure S11.

the effects of polygenic scores (PGSs), genetic predictors of a complex trait. Under this hypothesis, regardless of whether the PGS is estimated in a sample of males, females, or a combined sample of both males and females, it should be predictive in both sexes because the causal variants and the direction of their effects are shared, and the magnitude is correlated (Figure 1C, third model). At the same time, in the sex for which genetic effects are larger, the effect of the PGS is expected to be larger. To evaluate evidence of the systematic amplification model, we estimated PGSs based on our sex-specific GWASs and examined their effect in both sexes. For some traits, like albumin and lymphocyte percentage, the effects of the same PGS on trait value in males and females were statistically indistinguishable (Figures 2A, 2E, 2I, and 2J). In a few other traits, such as diastolic

blood pressure, the result was contingent on the sample in which the PGS was estimated (Figures 2C, 2G, 2I, and 2J). However, for roughly half of the traits examined, regardless of the sample from which the PGS was derived, the effect of the PGS was predictive in both sexes but significantly larger in one of the sexes (17 of 27 traits with t test $p < 0.05$ using the PGS derived from the males sample; 13 of 27 using the PGS derived from the females sample; Figures 2B, 2D, 2F, 2H, 2I, and 2J). These observations are consistent with systematic amplification.

The results presented in Figures 1 and 2 suggested to us that various modes of polygenic GxSex ought to be jointly evaluated. None of the hypothesized rules of thumb (Figure 1C) for interpreting genetic correlations and sex differences in heritability worked across all traits (see also a relevant discussion in Khramtsova et al.[9]). This motivated us to directly estimate the covariance between genetic effects in males and females. Another reason to treat covariance of genetic effects themselves as the estimand of interest is that multiple, distinct GxSex patterns may exist across subsets of genetic factors affecting a trait, depending on the pathways through which the subset acts, and whether and how the pathways are sex contingent (Figure 1C, fourth model).

Flexible model of sex-specific genetic effects as arising from a mixture of covariance relationships

We set to directly infer the mixture of covariance relationships of genetic effects among the sexes. We analyzed all traits in their raw measurement units as provided by the UKB. In particular, we did not normalize or standardize phenotypes within each sex before performing the sex-stratified GWAS because sex differences in trait variance may be partly due to amplification. Standardization would have therefore resulted in masking amplification signals that may exist in the data. In some cases, this is indeed the purpose of standardization.[44] More generally, while each scaling choice has it merits, we view the measurement of genetic effects in their raw units as the most biologically interpretable.

We used multivariate adaptive shrinkage (mash),[45] a tool that allows inference of genome-wide frequencies of genetic covariance relationships. We model the marginal SNP effect estimates as sampled (with SNP-specific, sex-specific noise) from a mixture of zero-centered normal distributions with various pre-specified covariance relationships (2×2 variance-covariance matrices for male and female effects; Equation 1 in Urbut et al.[45]). Our pre-specified covariance matrices ("hypothesis matrices") span a wide array of amplification and correlation relationships and use *mash* to estimate the mixture weights. Loosely, these weights can be interpreted as the proportion of variants that follow the pattern specified by the covariance matrix (Figure 3A). Our covariance matrices ranged from -1 to 1 in between-sex correlation and 10 levels of relative magnitude in females relative to males, including matrices corresponding to no effect in one or both sexes (Figure S2).

We first focus on testosterone, for which previous research sets the expectation for polygenic male-female covariance. In terms of magnitude, the vast majority of effects should have much greater effect in males. In terms of correlation, we expect a class of genetic effects acting through largely independent and uncorrelated pathways alongside a class of effects via shared pathways.[23] Independent pathways include the role of the hypothalamic-pituitary-gonadal axis in male testosterone regulation and the contrasting role of the adrenal gland in female testosterone production. Shared pathways involve sex hormone-binding globulin (SHBG), which decreases the amount of bioavailable testosterone in males and females. As expected, we found that mixture weights for testosterone concentrated on greater magnitudes in males and largely uncorrelated effects. Of the 32% total weights on matrices with an effect in at least one sex, 98% of the weights were placed on matrices representing larger effects in males, including 20.4% (±0.7%) having male-specific effects (Figures 3 and S5).

Amplification of genetic effects is the primary mode of GxSex

The only trait of the 27 where a large fraction ($\geq$ 10%) of non-zero effects was negatively correlated was testosterone (17%). Most effects were instead perfectly or near-perfectly correlated. For example, diastolic blood pressure and eosinophil percentage had 66% (Figure 3B) and 68% (Data S8) of effects being perfectly correlated, respectively. Overall, the low weights on matrices representing negative correlation do not support opposite directions of effects being a major mode of GxSex (Figure S7).

In some traits, such as hemoglobin A1C or diastolic blood pressure, previously considered non-sex specific because of high genetic correlations between sexes and a concordance in top GWAS hits, we find evidence of substantial GxSex through amplification (Figures 3B and 3F).[25,26] Furthermore, about half (13 of 27) of the traits analyzed had the majority of weights placed on greater effects in just one of the sexes (x axis in Figure 4A). For instance, 92% of effects on BMI-adjusted waist:hip ratio were greater in females, and 92% of effects on (right) arm fat-free mass were greater in males. Both traits had mixture weights concentrated on highly correlated effects (Figure 3). We confirmed, using a simulation study, that this summary of sex-biased amplification indeed captures sex differences in the magnitude of genetic effects and that it is not due to differences in the extent of estimation noise (e.g., variation in environmental factors independent of genetic effects; Figures S5 and S6; STAR Methods).

Across traits, the difference between the fraction of male-larger effects and the fraction of female-larger effects correlates strongly with male-to-female phenotypic variance ratio (Pearson $r = 0.873$, $p = 6 \times 10^{-9}$ after removing testosterone as an outlier; Figure 4A). This observation is consistent with our hypothesis of amplification leading to differences in genetic variance between sexes, thereby contributing substantially to sex differences in phenotypic variance. Together, these observations point to amplification, rather than uncorrelated effects, as a primary mode of polygenic GxSex.

Another important question about the implication of pervasive amplification is whether it is a major driver of mean phenotypic differences. The ratio between male and female phenotypic means is correlated with the difference between male-larger and female-larger amplification (Pearson $r = 0.75$, $p = 2 \times 10^{-5}$ after removing testosterone and BMI-adjusted waist:hip ratio as

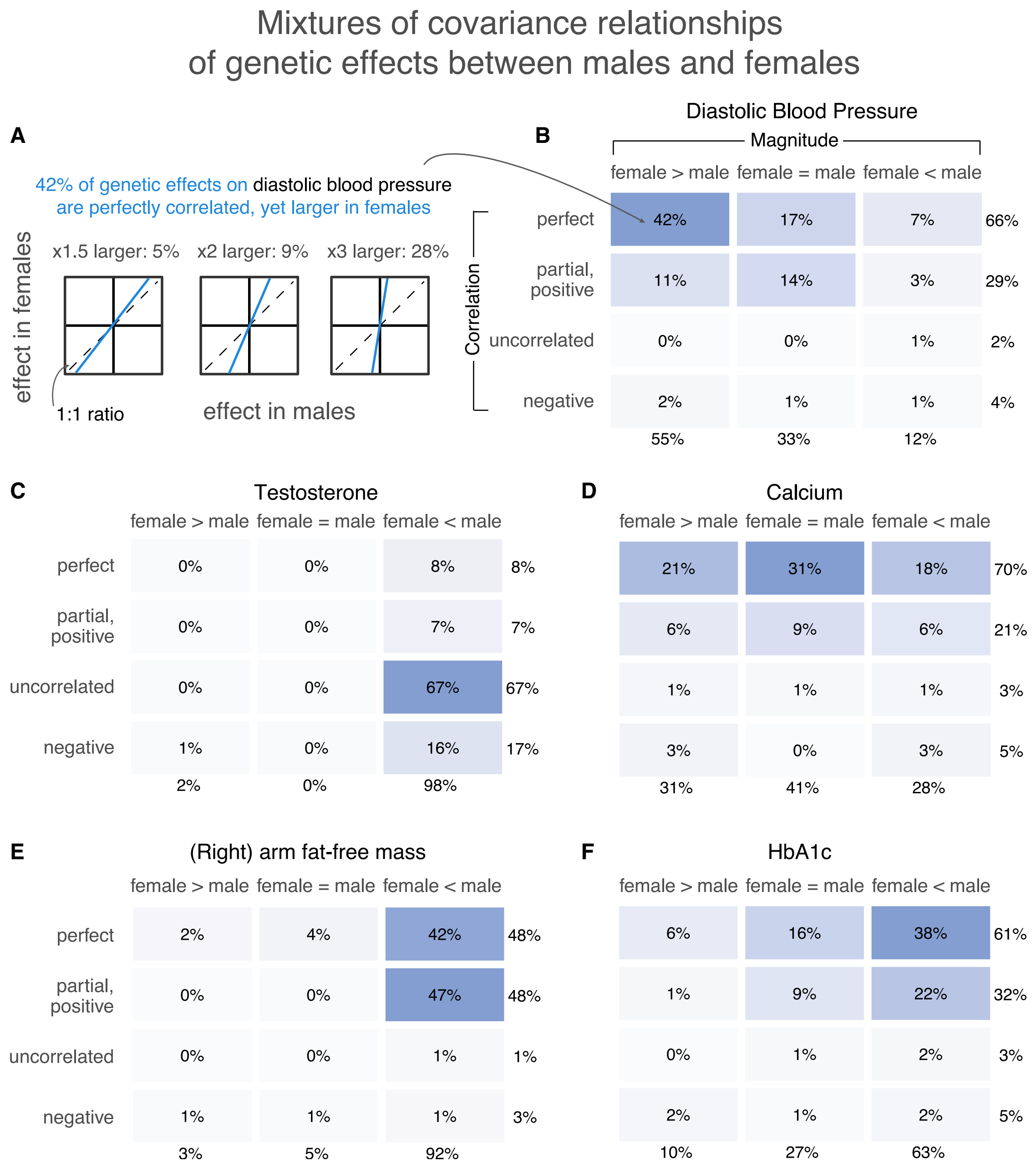

Figure 3. Polygenic covariance structure between males and females

(A) Our analysis of the polygenic covariance between males and females is based on sex-stratified GWASs. We modeled the sex-stratified GWAS estimates as sampled with error from true effects arising from a mixture of possible covariance relationships between female and male genetic effects. As an example, shown are illustrations for three possible relationships of the same qualitative nature—perfectly correlated effects that are also larger in females—and the mixture weights estimated for each in the case of diastolic blood pressure.

(B–F) Each box shows the sum of weights placed on all covariance relationships of the same qualitative nature, as specified by relative magnitude (horizontal axis) and correlation (vertical axis) between male and female effects. The full set of pre-specified covariance matrices is shown in Figure S2, and the weights placed on each of them for each trait are shown in Data S1–27. All weights shown are percentages of non-null weights; i.e., the weight divided by the sum of all weights except for the one corresponding to no effect in either sex.

outliers). Although this correlation is intriguing, within-sex GWAS aims to explain individual differences from the mean of the sex, and such GWAS results do not dictate the values of the sex means. Further, the ratio of mean trait values between sexes and the difference in amplification are strongly correlated with phenotypic variance ratios (Figure 4A; Figure S8; see also Karp et al.[8]), and many different causal accounts could explain these correlations.

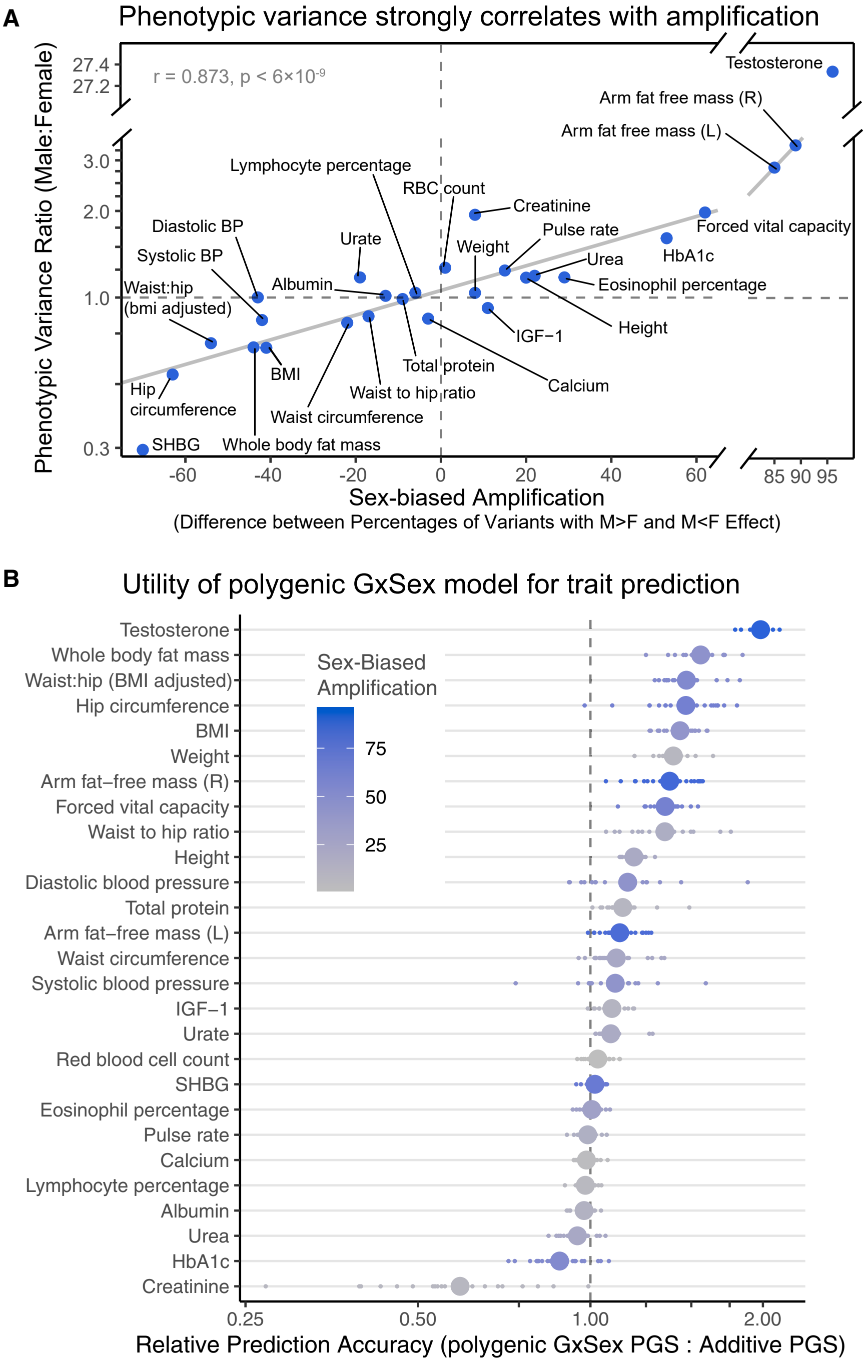

Figure 4. Consequences of amplification for trait variance and polygenic score predictive utility

(A) Phenotypic variance strongly correlates with amplification. “Sex-biased amplification” on the x axis is calculated by taking the difference between the sum of mixture weights on covariance matrices with male effects greater in magnitude than female effects (M > F) and the sum of weights of M < F matrices. The solid gray line shows a linear fit across traits, excluding testosterone as an outlier, with correlation summaries in gray in the top left corner.

(B) Utility of the polygenic GxSex model for trait prediction. The x axis shows the relative prediction accuracy estimated from the incremental R^2 ratio of a GxSex model informed by polygenic covariance patterns and an additive model. For each trait, smaller points show relative prediction accuracies across 20 cross-validation folds, and larger points show the average across the 20 folds. The phenotypes are ordered by the mean relative prediction accuracy. The color of each point corresponds to the degree of sex-biased amplification as described in (A).

Finally, the pervasiveness of GxSex, alongside the mixture of covariance relationships across the genome for many traits, may be important to consider in phenotypic prediction. We compared the prediction accuracy of PGSs that consider the polygenic covariance structure with that of additive models that ignore GxSex as well as models that include GxSex but do not consider the polygenic covariance structure (supplemental information; Figure S12). Indeed, for most traits (20 of 27 traits; Figure 4B), models that consider the polygenic covariance structure outperform all other models evaluated. Traits that showed better prediction accuracy using the model that considered polygenic covariance structure included many body mass-related traits, such as BMI and whole body fat mass, that also tended to have higher sex-based amplification (Figure 4B; Pearson $r = 0.56$, $p = 0.003$ between sex-biased amplification and prediction accuracy ratio). These results point to the utility of considering polygenic covariance structure in PGS prediction.

Testosterone as an amplifier

Thus far, we treated the genetic interaction as discretely mediated by biological sex. One mechanism that may underlie GxSex is a cue or exposure that modulates the magnitude (and less often the direction) of genetic effects and varies in its distribution between the sexes. As an example of such a cue, we considered testosterone. Testosterone may be a plausible instigator because the hormone is present in distinctive pathways and levels between the sexes and is a known contributor to the development of male secondary characteristics and therefore could modulate genetic causes on sex-differentiated traits.

To test this idea, we first binned individuals of each sex by their testosterone levels. Then, for each trait and within the bin, we quantified the magnitude of total genetic effect as the linear

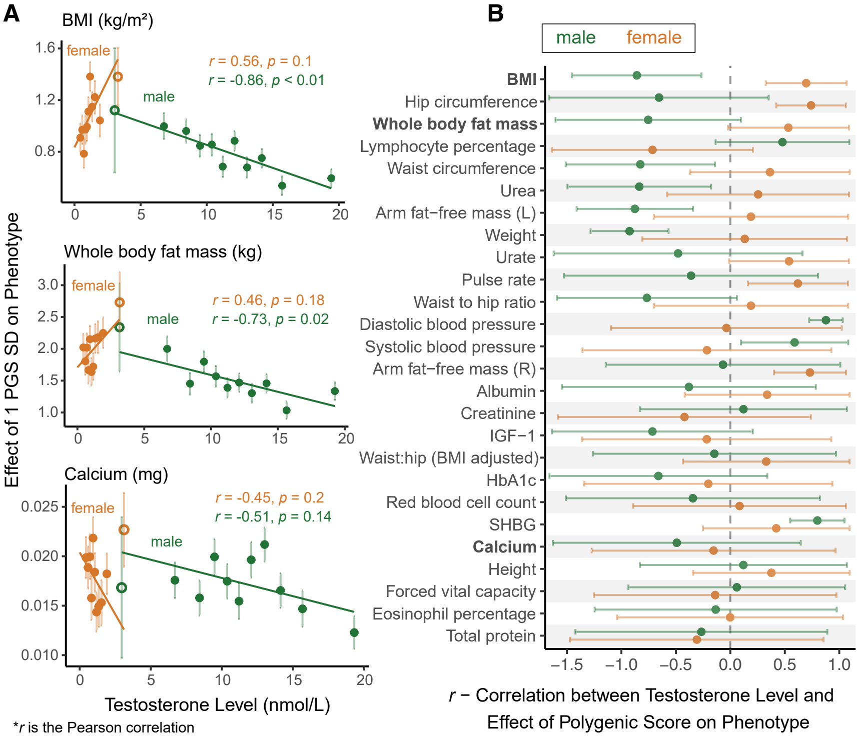

Figure 5. Amplification of total genetic effect in relation to testosterone levels

(A) The relationship between testosterone level bins and estimated magnitude of genetic effect on traits is shown for three traits. The magnitude of genetic effect is estimated using the slope of the regression of phenotypic values to PGSs in that bin. The units on the y axis are effect per standard deviation (SD) of the PGSs across all individuals in all bins. The hollow data points are bins with overlapping testosterone ranges between males and females; these are based on fewer individuals (∼800 compared with ∼2,200 in other bins) and not included in the regression. Figure S13 show all other traits analyzed.

(B) The correlation for each sex (90% CI) are shown for all 27 traits. Traits are ordered in descending order of male-female differences in Pearson correlation.

regression coefficient of trait values to a PGS for the trait (STAR Methods; see Figure S14 for results obtained using sex-specific PGSs). For BMI, testosterone (mean per bin) and the magnitude of genetic effect were correlated for males and females (Pearson $p < 0.05$; Figure 5A). For all body mass-related traits, there was a negative correlation between the magnitude of genetic effect and testosterone levels for males and a positive correlation for females (Figure 5B). Because the relationship with testosterone remains contingent on sex, a model of testosterone as the sole driver of the observed sex specificity would be invalid. These observations may help explain previous reports of positive correlations between obesity and free testosterone in women and negative correlations in men.[46] We conclude that, in body mass-related traits, testosterone may be modulating genetic effects in a sexually antagonistic manner.

We performed two additional analyses designed to control for possible caveats to the association of testosterone and the magnitude of polygenic effect. First, a test that controls for possible confounding with age (Figure S16). Second, a test that mitigates confounding with other variables or reverse causality (where the magnitude of genetic effect affecting the focal trait causally affecting testosterone levels; Figure S15). The evidence of an effect of testosterone on the magnitude of polygenic effect did not remain statistically significant in either of these tests. It is possible, however, that this was due to the low statistical power of these more conservative analyses (STAR Methods).

Are polygenic and environmental effects jointly amplified?

Our results thus far suggest that polygenic amplification across sexes is pervasive across traits and that the ratio of phenotypic variance scales with amplification (Figure 4A). An immediate question of interest is whether the same modulators that act on the magnitude of genetic effects act on environmental effects as well (see also a relevant discussion by Domingue et al.[47]). Consider the example of human skeletal muscle. The impact of resistance exercise varies between males and females. Resistance exercise can be considered an environmental effect because it upregulates multiple skeletal muscle genes present in males and females, such as insulin growth factor 1 (IGF-1), which, in turn, is involved in muscle growth.[48] However, after resistance exercises at similar intensities, upregulation of such genes is sustained in males, while levels return sooner to the resting state in females (Figure S17). It is plausible that modulators of the effect of IGF-1, such as insulin[49] or sex hormones,[50,51] drive a difference in the magnitude of effect of core genes such as IGF-1 in a sex-specific manner. To express this intuition with a model: if amplification mechanisms are shared, then amplification may be modeled as having the same scalar multiplier effect on genetic and environmental effects (Figure 6A). In the STAR Methods, we specify the details of a null model of joint amplification, which yields the prediction that the male-female ratio of genetic variances should equal the respective ratio of environmental variances (blue line in Figure 6B). As we explain in the supplemental information, this expectation is qualitatively different from those of two longstanding "rule of thumb" predictions for sex differences in trait variance[52]: the "greater male variability" and "estrus-mediated variability" models, which provide a poor fit across the 27 physiological traits analyzed (Figure S18B).

We tested the fit of the theoretical prediction under pervasive joint amplification across traits. We used our estimates of sex-specific phenotypic variance and SNP heritabilities to estimate the ratios of genetic and environmental variances. We note that environmental variance is proxied here by all trait variance not due to additive genetic effects, and caution is advised with interpretation of this proxy. Twenty of the 27 traits were

CellPress
OPEN ACCESS

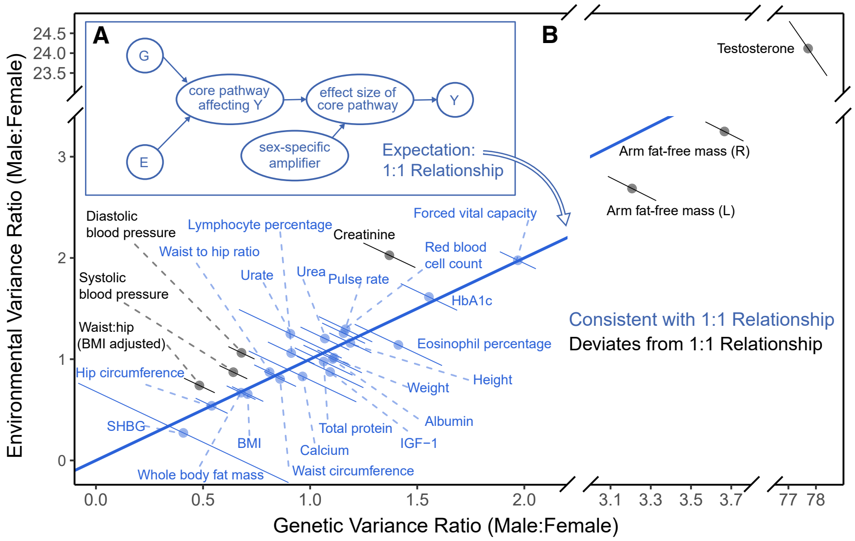

Figure 6. Testing a model of pervasive, joint amplification of environmental and polygenic effects

(A) A model of equal amplification of genetic (G) and environmental (E) effect that produces the sex differences in the distribution of the phenotype, Y. G and E act through a core pathway that is amplified in a sex-specific manner.

(B) The blue 1:1 line depicts the theoretical expectation under a simple model of equal amplification of G and E effects in males compared with females. Error bars show 90% confidence intervals. Traits in blue are consistent (within their 90% CI) with the theoretical prediction. Figure S18 shows the same data alongside the predictions under other theoretical models of male-female variance ratios.

consistent with the null model of pervasive joint amplification (within 90% confidence interval [CI]; Figure 6B). This finding may suggest a sharing of pathways between polygenic and environmental effects for these traits (Figure 6A). Interesting exceptions include diastolic blood pressure, which was the strongest outlier ($p = 3.06 \times 10^{-12}$, single-sample z test), excluding testosterone.

Sexually antagonistic selection

A hypothesized cause of sexual dimorphism is sexually antagonistic selection, in which some alleles are beneficial in one sex but deleterious in the other.[11,12,14,53,54] Sexually antagonistic selection is difficult to study using traditional population genetics methods because Mendelian inheritance equalizes autosomal allele frequencies between the sexes at conception, thereby erasing informative signals. One way around this limitation is to examine allele frequency differences between the sexes in the current generation, known as "selection in real time."[14,55,56] In this section, we consider a model of sexually antagonistic selection acting on a polygenic trait and use it to estimate the strength of contemporary viability selection acting on the 27 traits we analyzed.

Most theoretical models of sexually antagonistic selection on a trait under stabilizing selection usually posit either highly distinct male and female fitness optima or genetic variants affecting traits antagonistically. Our findings on pervasive amplification suggest that variant effects on traits tend to have concordant signs. However, under pervasive amplification, a somewhat surprising intuition arises. Alleles affecting a trait may frequently experience sexually antagonistic selection in the case in which trait optima for males and females are very distinct (Figure 7B) and for the case in which they are similar (Figure 7A).

We developed a theoretical model of sexually antagonistic viability selection on a single trait that builds on this intuition. The model relates sex-specific effects on a complex trait to the divergence in allele frequency between males and females (measured as F_{ST}[57,58]) because of viability selection "in real time"; i.e., acting in the current generation between conception and the time of sampling. We derive the expected relationship for each site i,

$$F_{ST\,i} \approx A V_{GxS\,i}, \qquad \text{(Equation 1)}$$

where

$$V_{\{GxS\}_i} = 2p_i(1 - p_i)\left(\beta_i^m - \beta_i^f\right)^2,$$

and p_i, β_i^m and β_i^f are the allele frequency of an allele at site i, its effect on the trait in males, and its effect in females, respectively. A is a constant parameter shared across all variants and can therefore be interpreted as the effect of sexually antagonistic selection on male-female divergence at variants associated with the trait (STAR Methods). We estimated $F_{ST\,i}$ for all sites i across subsamples of various ancestry groups in the gnomAD dataset.[59] To estimate $V_{\{GxS\}_i}$ at each site and for each trait, we used our sex-stratified GWAS results. Because there is large heterogeneity in uncertainty of GxSex-genetic variance estimates, we use a variance-weighted linear regression to estimate A (see STAR Methods for the derivation of the variance of $V_{\{GxS\}_i}$ estimates and supplemental information for further details).

Recent work has shown that apparent sex differences in autosomal allele frequencies within a sample are often due to a bioinformatic artifact: mismapping of sequencing reads from autosomes to sex chromosomes or vice versa.[53,60,61] We identified and excluded sites that are potentially vulnerable to this artifact (supplemental information). In Figure 7D, we only show results for gnomAD subsamples that are the closest in their genetic ancestry to our UKB sample[62] (results for other subsamples are shown in Figures S19 and S20). Furthermore, given the concerns of study recruitment biases,[43,60] we place higher confidence in results that replicate qualitatively across different subsamples, even though we note that subsample-specific selection signals may be real because sexually antagonistic selection may act heterogeneously across groups.

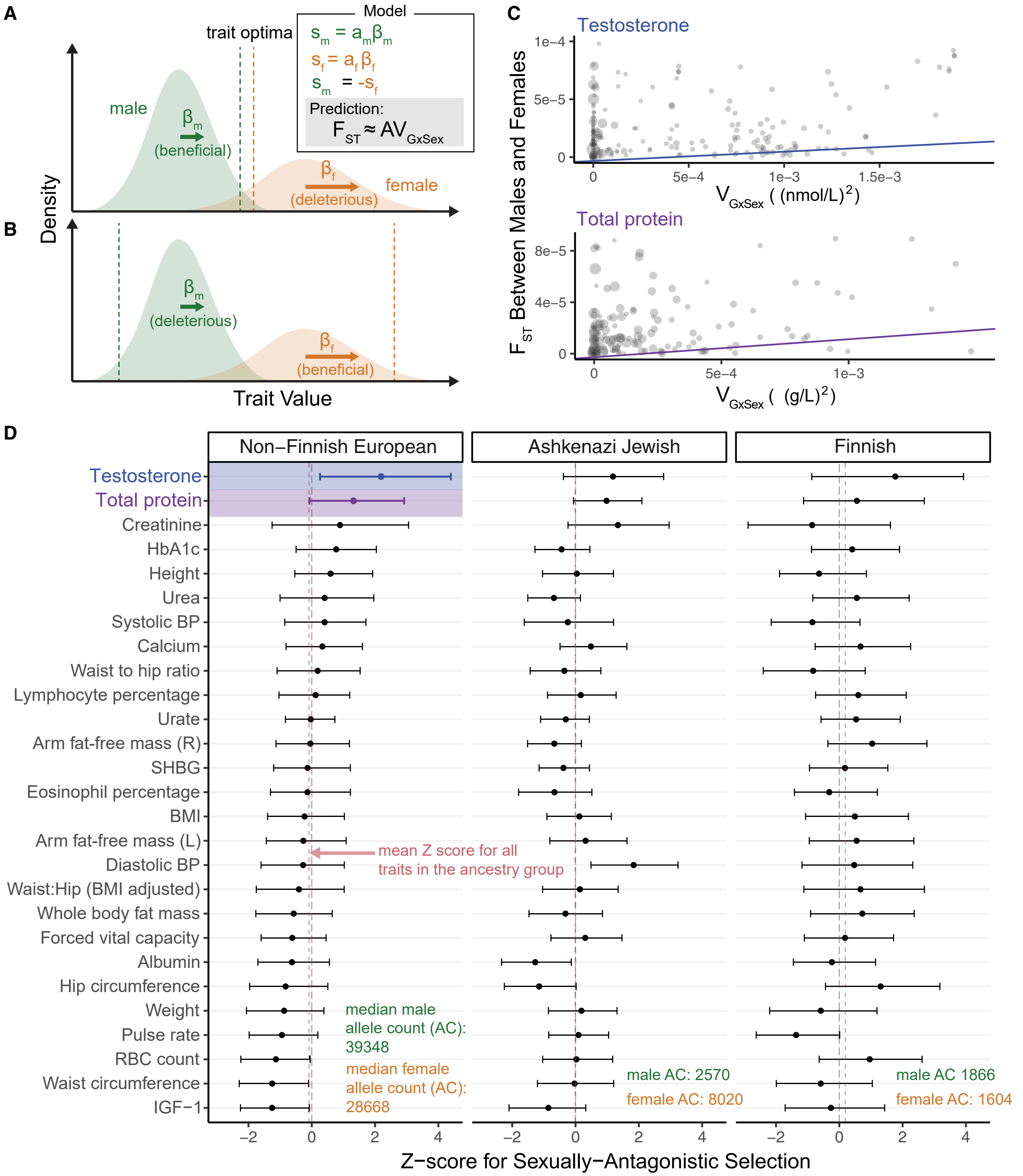

Figure 7. Testing for sexually antagonistic selection

(A and B) A model of sexually antagonistic selection. Selection coefficients, s_m and s_f, are linear with the additive effect on the trait in each sex. Sexually antagonistic selection acts so that $s_m = -s_f$. The model yields the prediction of Equation 1. While in (A), trait optima are close to each other and in (B) they are far apart, in both cases alleles will tend to be antagonistically selected.

(legend continued on next page)

With these conservative criteria considered, we only find evidence of sexually antagonistic polygenic selection on testosterone. In the non-Finnish sample, the largest of the three samples, the null hypothesis $H_0 : A = 0$ in Equation 1 is rejected ($p < 0.05$) only for testosterone (Z score = 2.2). Testosterone is among the three strongest signals in the two other samples as well, although none of the traits are statistically significant in these samples.

DISCUSSION

Departing from previous studies that sought GxSex through single loci or heritability analyses, we modeled GxSex as a mixture of polygenic relationships across the genome. Our analysis supports pervasive context dependency of genetic effects on complex traits, acting largely through amplification. Surprisingly, even for some traits such as red blood cell count, previously considered non-sex specific because of high genetic correlations between sexes and a concordance in top GWAS hits, we find evidence of substantial GxSex. The strong relationships we find between amplification, environmental variance, and phenotypic variance further point to its potential importance for sex differences.

We have shown that considering the polygenic covariance structure, including amplification signals, improves phenotypic prediction for most traits. Its incorporation in PGSs is straightforward. We therefore recommend its broad application and further building on our approach to improve clinical risk stratification and other applications of PGSs.

Our findings may seem at odds with previous reports of GxSex primarily consisting of sex-limited effects (i.e., no effect in one of the sexes) or antagonistic effects (differences in sign).[63] In the supplemental information and Table S6, we illustrate that these apparent discrepancies may be rooted in ascertainment biases. Therefore, limiting analyses to variants with outsized sex differences provides a clouded picture of polygenic GxSex.

Localization of GxSex signals can provide clues regarding the modulators underlying amplification. Here, we proposed one such modulator, testosterone, and found a correlation between testosterone levels and the magnitude of genetic effect on whole body fat mass. The opposite signs of these correlations in females and males may reflect the discrepant relationship between testosterone and these traits at the phenotypic level.

Our approach for studying GxSex in complex physiological traits can be adopted to study the moderation of polygenic effects by other environments. Starting out with sex as an environmental variable offers a methodological advantage. The study of context dependency in humans is often complicated by study participation biases, leading to a genetic ancestry structure that confounds genotype-phenotype associations,[43,64–66] reverse causality between the phenotype and environment variable, collider bias, gene-by-environment correlation, and other problems.[67–69] Focusing on sex as a case study circumvents many of these ‘‘usual suspect’’ problems; for example, problems involving the phenotype causally affecting sex are unlikely. This is an important benchmark for future studies of environmental modulation because of the methodological advantage of sex as an environmental variable and because sex is almost always measured; so insight into sex differences in genetic effects can be incorporated straightforwardly in future studies and in clinical risk prediction. Here, we showed that, for most of the traits considered, modeling polygenic GxSex (as opposed to individually estimating sex-specific effects at each site; Figure S12) yields sex-specific predictors that outperform standard additive PGSs.

Finally, we developed a model—the first to our knowledge—that considers how GxSex may fuel sexually antagonistic selection on complex traits. Over long evolutionary timescales, the two scenarios depicted in Figures 7A and 7B may lead to different predictions about the long-term maintenance of GxSex genetic variance. Regardless, in both cases, alleles that underlie GxSex may experience sexually antagonistic selection.

We found suggestive signals of sexually antagonistic selection on variation associated with testosterone levels (also see related results by Ruzicka et al.[56]). The signal for our inference of selection is systematic allele frequency differences between adult males and females, which are consistent with contemporary viability selection. The severity, age of onset, and prevalence of nearly all diseases are sexually dimorphic.[70] These signals may therefore point to a related disease that differentially affects lifespan in the two sexes, such as immune system suppression, diabetes, cancers, and hypertension.[71–74] Recently, high testosterone levels have been linked to increased rates of mortality and cancer in women but decreased rates in men.[75,76] However, the testosterone result is also consistent with other accounts, such as testosterone having opposing effects on the propensity to participate in a study in the two sexes. Further validation is therefore required to better test hypotheses of sexually antagonistic selection; for example, in studies with no recruitment biases (or at least distinct recruitment biases).

In this work, we have shown that amplification of the magnitude of polygenic effects may be important to consider as a driver of sex differences and their evolution. Our approach included the flexible modeling of genetic effect covariance among the sexes, as well as various subsequent analyses exploring the implications of these covariance structures. We hope this study can inform future work on the context specificity of genetic effects on complex traits.

Limitations of the study

Study participation in large biobanks like the UKB differs by sex,[77] and work by Pirastu et al.[60] further argued that allele frequency differences between males and females may reflect sex-specific recruitment biases. However, a recent study by Benonisdottir and Kong[43] found no evidence of sex-specific genetic associations with UKB participation, and another by

(C) Two examples of the weighted least-squares linear regression performed to estimate the strength of sexually antagonistic selection on variants associated with a trait (A in A and Equation 1). Each point shows one SNP. Size is proportional to each point's regression weight.

(D) Z scores (90% non-parametric bootstrap CI) estimated through 1,000 resampling iterations of the weighted linear regression of (B) for each trait. The two colored estimates correspond to the examples in (B) and (C).

Kasimatis et al.[53] showed that many apparent associations of autosomal genotypes and biological sex in the UKB were instead primarily due to a bioinformatic artifact: mis-hybridization of autosomal genotyping probes with sex chromosomes. Even still, subtle recruitment biases affecting male and female participation differently remains a possible caveat to our conclusions. For the analysis of natural selection, the replication of signals of selection in multiple samples may lend some credence to our inference. Nevertheless, in medical datasets based on recruitment of participants via referring physicians, recruitment biases may still plausibly be shared across studies.

Another limitation of the study is the inability to directly test the hypothesis about pervasive, joint amplification of genetic and environmental effects. While the data available to us are consistent with the hypothesis (Figures 6 and S19), they are also consistent with other possible explanations and susceptible to caveats. For example, our proxy for environmental variance includes, to an unknown extent, genetic variance, which is not well tagged by the UKB genotype array. Further study is required to robustly test this hypothesis, but it may require detailed data on environmental effects on a complex trait in females and males.

STAR★METHODS

Detailed methods are provided in the online version of this paper and include the following:

- KEY RESOURCES TABLE
- RESOURCE AVAILABILITY
  - Lead contact
  - Materials availability
  - Data and code availability
- METHOD DETAILS
  - UK Biobank sample characteristics
  - Expectations for sex-specific heritabilities with no GxSex
  - Multivariate adaptive shrinkage (mash)
  - Mixture weights for covariance structure between male and female effects
  - Choice of SNPs used to estimate male-female effect covariance
  - Simulating equal genetic effects and heterogeneous estimation noise among the sexes
  - Simulating sex-biased amplification
  - Testosterone as an amplifier
  - Model of shared amplification
  - A model of sexually antagonistic selection
  - Estimating the potential for sexually antagonistic selection on standing variation (*A*)

SUPPLEMENTAL INFORMATION

Supplemental information can be found online at https://doi.org/10.1016/j.xgen.2023.100297.

ACKNOWLEDGMENTS

We thank the Harpak Lab and Edge Lab members Ziyue Gao, Tom Juenger, Jonathan Pritchard, Molly Przeworski, Guy Sella, Jeff Spence, and Elliot Tucker-Drob for helpful comments on the manuscript. We also thank Brian Dilkes, Andrés Bendesky, and Jim Fleet for insightful discussions. We thank Abin Abraham and Tony Capra for help with implementing code from Kasimatis et al.[53] and Michelle Traglia and Lauren Weiss for useful discussions of the relationship between the results reported here and Traglia et al.[63] This work was supported by NIH GM116853-07 (to M.K.) and NIH GM137758 (to M.D.E.). This study was conducted using the UK Biobank resource under application 61666, as approved by the University of Texas at Austin institutional review board (protocol 2019-02-0125).

AUTHOR CONTRIBUTIONS

C.Z., M.J.M., and A.H. designed the experiments. C.Z. and M.J.M. performed the experiments. C.Z. and A.H. wrote the paper with assistance from all authors. J.M.C., M.D.E., and M.K. provided expertise and feedback.

DECLARATION OF INTERESTS

The authors declare no competing interests.

Received: May 16, 2022
Revised: December 15, 2022
Accepted: March 13, 2023
Published: April 6, 2023

REFERENCES

1. van Doorn, G.S. (2009). Intralocus sexual conflict. Ann. N. Y. Acad. Sci. *1168*, 52–71. https://doi.org/10.1111/j.1749-6632.2009.04573.x.
2. Arnqvist, G., and Rowe, L. (2005). Sexual Conflict (Princeton University Press). https://doi.org/10.1515/9781400850600.
3. Camus, M.F., Piper, M.D., and Reuter, M. (2019). Sex-specific transcriptomic responses to changes in the nutritional environment. Elife *8*, e47262. https://doi.org/10.7554/eLife.47262.
4. Bayer, E.A., Stecky, R.C., Neal, L., Katsamba, P.S., Ahlsen, G., Balaji, V., Hoppe, T., Shapiro, L., Oren-Suissa, M., and Hobert, O. (2020). Ubiquitin-dependent regulation of a conserved DMRT protein controls sexually dimorphic synaptic connectivity and behavior. Elife *9*, e59614. https://doi.org/10.7554/eLife.59614.
5. Baar, E.L., Carbajal, K.A., Ong, I.M., and Lamming, D.W. (2016). Sex- and tissue-specific changes in mTOR signaling with age in C57 BL/6J mice. Aging Cell *15*, 155–166. https://doi.org/10.1111/acel.12425.
6. Wat, L.W., Chao, C., Bartlett, R., Buchanan, J.L., Millington, J.W., Chih, H.J., Chowdhury, Z.S., Biswas, P., Huang, V., Shin, L.J., et al. (2020). A role for triglyceride lipase brummer in the regulation of sex differences in Drosophila fat storage and breakdown. PLoS Biol. *18*, e3000595. https://doi.org/10.1371/journal.pbio.3000595.
7. Wat, L.W., Chowdhury, Z.S., Millington, J.W., Biswas, P., and Rideout, E.J. (2021). Sex determination gene transformer regulates the male-female difference in Drosophila fat storage via the adipokinetic hormone pathway. Elife *10*, e72350. https://doi.org/10.7554/eLife.72350.
8. Karp, N.A., Mason, J., Beaudet, A.L., Benjamini, Y., Bower, L., Braun, R.E., Brown, S.D.M., Chesler, E.J., Dickinson, M.E., Flenniken, A.M., et al. (2017). Prevalence of sexual dimorphism in mammalian phenotypic traits. Nat. Commun. *8*, 15475. https://doi.org/10.1038/ncomms15475.
9. Khramtsova, E.A., Davis, L.K., and Stranger, B.E. (2019). The role of sex in the genomics of human complex traits. Nat. Rev. Genet. *20*, 173–190. https://doi.org/10.1038/s41576-018-0083-1.
10. Barson, N.J., Aykanat, T., Hindar, K., Baranski, M., Bolstad, G.H., Fiske, P., Jacq, C., Jensen, A.J., Johnston, S.E., Karlsson, S., et al. (2015). Sex-dependent dominance at a single locus maintains variation in age at maturity in salmon. Nature *528*, 405–408. https://doi.org/10.1038/nature16062.

CellPress
OPEN ACCESS

11. Kidwell, J.F., Clegg, M.T., Stewart, F.M., and Prout, T. (1977). Regions of stable equilibria for models of differential selection in the two sexes under random mating. Genetics *85*, 171–183. https://doi.org/10.1093/genetics/85.1.171.

12. Connallon, T., Cox, R.M., and Calsbeek, R. (2010). Fitness consequences of sex-specific selection. Evolution *64*, 1671–1682. https://doi.org/10.1111/j.1558-5646.2009.00934.x.

13. Harrison, P.W., Wright, A.E., Zimmer, F., Dean, R., Montgomery, S.H., Pointer, M.A., and Mank, J.E. (2015). Sexual selection drives evolution and rapid turnover of male gene expression. Proc. Natl. Acad. Sci. USA *112*, 4393–4398. https://doi.org/10.1073/pnas.1501339112.

14. Cheng, C., and Kirkpatrick, M. (2016). Sex-specific selection and sex-biased gene expression in humans and flies. PLoS Genet. *12*, e1006170. https://doi.org/10.1371/journal.pgen.1006170.

15. Schroderus, E., Jokinen, I., Koivula, M., Koskela, E., Mappes, T., Mills, S.C., Oksanen, T.A., Poikonen, T., Ketterson, A.E.E.D., and McPeek, E.M.A. (2010). Intra- and intersexual trade-offs between testosterone and immune system: implications for sexual and sexually antagonistic selection. Am. Nat. *176*, E90–E97.

16. Power, R.A., Kyaga, S., Uher, R., MacCabe, J.H., Långström, N., Landen, M., McGuffin, P., Lewis, C.M., Lichtenstein, P., and Svensson, A.C. (2013). Fecundity of patients with schizophrenia, autism, bipolar disorder, depression, anorexia nervosa, or substance abuse vs their unaffected siblings. JAMA Psychiatr. *70*, 22–30. https://doi.org/10.1001/jamapsychiatry.2013.268.

17. Mokkonen, M., and Crespi, B.J. (2015). Genomic conflicts and sexual antagonism in human health: insights from oxytocin and testosterone. Evol. Appl. *8*, 307–325. https://doi.org/10.1111/eva.12244.

18. Harper, J.A., Janicke, T., and Morrow, E.H. (2021). Systematic review reveals multiple sexually antagonistic polymorphisms affecting human disease and complex traits. Evolution *75*, 3087–3097. https://doi.org/10.1111/evo.14394.

19. Oliva, M., Muñoz-Aguirre, M., Kim-Hellmuth, S., Wucher, V., Gewirtz, A.D.H., Cotter, D.J., Parsana, P., Kasela, S., Balliu, B., Viñuela, A., et al. (2020). The impact of sex on gene expression across human tissues. Science *369*, eaba3066. https://doi.org/10.1126/science.aba3066.

20. Clayton, J.A., and Collins, F.S. (2014). Policy: NIH to balance sex in cell and animal studies. Nature *509*, 282–283. https://doi.org/10.1038/509282a.

21. Clayton, J.A. (2016). Studying both sexes: a guiding principle for biomedicine. FASEB J. *30*, 519–524. https://doi.org/10.1096/fj.15-279554.

22. (2022). Nature journals raise the bar on sex and gender reporting in research. Nature *605*, 396. https://doi.org/10.1038/d41586-022-01218-9.

23. Sinnott-Armstrong, N., Naqvi, S., Rivas, M., and Pritchard, J.K. (2021). Gwas of three molecular traits highlights core genes and pathways alongside a highly polygenic background. Elife *10*, e58615–e58635. https://doi.org/10.7554/eLife.58615.

24. Hooven, C. (2021). T: The Story of Testosterone, the Hormone that Dominates and Divides Us (Henry Holt and Co.).

25. Flynn, E., Tanigawa, Y., Rodriguez, F., Altman, R.B., Sinnott-Armstrong, N., and Rivas, M.A. (2021). Sex-specific genetic effects across biomarkers. Eur. J. Hum. Genet. *29*, 154–163. https://doi.org/10.1038/s41431-020-00712-w.

26. Bernabeu, E., Canela-Xandri, O., Rawlik, K., Talenti, A., Prendergast, J., and Tenesa, A. (2021). Sex differences in genetic architecture in the UK Biobank. Nat. Genet. *53*, 1283–1289. https://doi.org/10.1038/s41588-021-00912-0.

27. Muir, W., Nyquist, W.E., and Xu, S. (1992). Alternative partitioning of the genotype-by-environment interaction. Theor. Appl. Genet. *84*, 193–200. https://doi.org/10.1007/BF00224000.

28. Robertson, A. (1959). The sampling variance of the genetic correlation coefficient. Biometrics *15*, 469. https://doi.org/10.2307/2527750.

29. Falconer, D.S. (1952). The problem of environment and selection. Am. Nat. *86*, 293–298.

30. Fry, J.D. (1992). The mixed-model analysis of variance applied to quantitative genetics: biological meaning of the parameters. Evolution *46*, 540–550. https://doi.org/10.2307/2409870.

31. Yamada, Y. (1962). Genotype by environment interaction and genetic correlation of the same trait under different environments. Jpn. J. Genet. *37*, 498–509. https://doi.org/10.1266/jjg.37.498.

32. Brown, B.C., Asian Genetic Epidemiology Network Type 2 Diabetes Consortium; Ye, C.J., Price, A.L., and Zaitlen, N. (2016). Transethnic genetic-correlation estimates from summary statistics. Am. J. Hum. Genet. *99*, 76–88. https://doi.org/10.1016/j.ajhg.2016.05.001.

33. Galinsky, K.J., Reshef, Y.A., Finucane, H.K., Loh, P.-R., Zaitlen, N., Patterson, N.J., Brown, B.C., and Price, A.L. (2019). Estimating cross-population genetic correlations of causal effect sizes. Genet. Epidemiol. *43*, 180–188. https://doi.org/10.1002/gepi.22173.

34. Ni, G., Moser, G., Schizophrenia Working Group of the Psychiatric Genomics Consortium; Wray, N.R., and Lee, S.H. (2018). Estimation of genetic correlation via linkage disequilibrium score regression and genomic restricted maximum likelihood. Am. J. Hum. Genet. *102*, 1185–1194. https://doi.org/10.1016/j.ajhg.2018.03.021.

35. Shi, H., Mancuso, N., Spendlove, S., and Pasaniuc, B. (2017). Local genetic correlation gives insights into the shared genetic architecture of complex traits. Am. J. Hum. Genet. *101*, 737–751. https://doi.org/10.1016/j.ajhg.2017.09.022.

36. Bulik-Sullivan, B., Finucane, H.K., Anttila, V., Gusev, A., Day, F.R., Loh, P.-R., ReproGen Consortium; Psychiatric Genomics Consortium; Genetic Consortium for Anorexia Nervosa of the Wellcome Trust Case Control Consortium 3; and Duncan, L., et al. (2015). An atlas of genetic correlations across human diseases and traits. Nat. Genet. *47*, 1236–1241. https://doi.org/10.1038/ng.3406.

37. DiMarco, M., Zhao, H., Boulicault, M., and Richardson, S.S. (2022). Why "sex as a biological variable" conflicts with precision medicine initiatives. Cell Rep. Med. *3*, 100550. https://doi.org/10.1016/j.xcrm.2022.100550.

38. Lumish, H.S., O'Reilly, M., and Reilly, M.P. (2020). Sex differences in genomic drivers of adipose distribution and related cardiometabolic disorders: opportunities for precision medicine. Arterioscler. Thromb. Vasc. Biol. *40*, 45–60. https://doi.org/10.1161/ATVBAHA.119.313154.

39. Boyle, E.A., Li, Y.I., and Pritchard, J.K. (2017). An expanded view of complex traits: from polygenic to omnigenic. Cell *169*, 1177–1186. https://doi.org/10.1016/j.cell.2017.05.038.

40. Shi, H., Kichaev, G., and Pasaniuc, B. (2016). Contrasting the genetic architecture of 30 complex traits from summary association data. Am. J. Hum. Genet. *99*, 139–153. https://doi.org/10.1016/j.ajhg.2016.05.013.

41. Sella, G., and Barton, N.H. (2019). Thinking about the evolution of complex traits in the era of genome-wide association studies. Annu. Rev. Genomics Hum. Genet. *20*, 461–493. https://doi.org/10.1146/annurev-genom-083115-022316.

42. Bulik-Sullivan, B.K., Loh, P.-R., Finucane, H.K., Ripke, S., Yang, J., Schizophrenia Working Group of the Psychiatric Genomics Consortium; Patterson, N., Daly, M.J., Price, A.L., and Neale, B.M. (2015). LD Score regression distinguishes confounding from polygenicity in genome-wide association studies. Nat. Genet. *47*, 291–295. https://doi.org/10.1038/ng.3211.

43. Benonisdottir, S., and Kong, A. (2022). The genetics of participation: method and analysis. Preprint at bioRxiv. https://doi.org/10.1101/2022.02.11.480067.

44. Lynch, M., and Walsh, B. (1998). Genetics and Analysis of Quantitative Traits, 1st ed. (Sinauer Associates).

45. Urbut, S.M., Wang, G., Carbonetto, P., and Stephens, M. (2019). Flexible statistical methods for estimating and testing effects in genomic studies with multiple conditions. Nat. Genet. *51*, 187–195. https://doi.org/10.1038/s41588-018-0268-8.

46. Pasquali, R. (2006). Obesity and androgens: facts and perspectives. Fertil. Steril. *85*, 1319–1340. https://doi.org/10.1016/j.fertnstert.2005.10.054.

47. Domingue, B.W., Kanopka, K., Mallard, T.T., Trejo, S., and Tucker-Drob, E.M. (2022). Modeling interaction and dispersion effects in the analysis of gene-by-environment interaction. Behav. Genet. *52*, 56–64. https://doi.org/10.1007/s10519-021-10090-8.

48. Liu, D., Sartor, M.A., Nader, G.A., Gutmann, L., Treutelaar, M.K., Pistilli, E.E., Iglayreger, H.B., Burant, C.F., Hoffman, E.P., and Gordon, P.M. (2010). Skeletal muscle gene expression in response to resistance exercise: sex specific regulation. BMC Genom. *11*, 659. https://doi.org/10.1186/1471-2164-11-659.

49. Lutz, S.Z., Wagner, R., Fritsche, L., Peter, A., Rettig, I., Willmann, C., Fehlert, E., Martus, P., Todenhöfer, T., Stefan, N., et al. (2019). Sex-specific associations of testosterone with metabolic traits. Front. Endocrinol. *10*, 90. https://doi.org/10.3389/fendo.2019.00090.

50. Kraemer, W.J., Ratamess, N.A., and Nindl, B.C. (2017). Recovery responses of testosterone, growth hormone, and IGF-1 after resistance exercise. J. Appl. Physiol. *122*, 549–558. https://doi.org/10.1152/japplphysiol.00599.2016.

51. Roberts, B.M., Nuckols, G., and Krieger, J.W. (2020). Sex differences in resistance training: a systematic review and meta-analysis. J. Strength Cond. Res. *34*, 1448–1460. https://doi.org/10.1519/JSC.0000000000003521.

52. Zajitschek, S.R., Zajitschek, F., Bonduriansky, R., Brooks, R.C., Cornwell, W., Falster, D.S., Lagisz, M., Mason, J., Senior, A.M., Noble, D.W., and Nakagawa, S. (2020). Sexual dimorphism in trait variability and its eco-evolutionary and statistical implications. Elife *9*, e63170. https://doi.org/10.7554/eLife.63170.

53. Kasimatis, K.R., Abraham, A., Ralph, P.L., Kern, A.D., Capra, J.A., and Phillips, P.C. (2021). Evaluating human autosomal loci for sexually antagonistic viability selection in two large biobanks. Genetics *217*, 1–10. https://doi.org/10.1093/genetics/iyaa015.

54. Ryan, M. (2020). The Genetics of Political Behavior, 1st ed. (Routledge).

55. Ruzicka, F., Holman, L., and Connallon, T. (2021). Polygenic signals of sexually antagonistic selection in contemporary human genomes. Preprint at bioRxiv. https://doi.org/10.1101/2021.09.20.461171.

56. Ruzicka, F., Dutoit, L., Czuppon, P., Jordan, C.Y., Li, X.Y., Olito, C., Runemark, A., Svensson, E.I., Yazdi, H.P., and Connallon, T. (2020). The search for sexually antagonistic genes: practical insights from studies of local adaptation and statistical genomics. Evol. Lett. *4*, 398–415. https://doi.org/10.1002/evl3.192.

57. Wright, S. (1951). The genetical structure of populations. Ann. Eugen. *15*, 323–354. https://doi.org/10.1111/j.1469-1809.1949.tb02451.x.

58. Weir, B.S., and Cockerham, C.C. (1984). Estimating F-statistics for the analysis of population structure. Evolution *38*, 1358–1370. https://doi.org/10.2307/2408641.

59. Karczewski, K.J., Francioli, L.C., Tiao, G., Cummings, B.B., Alföldi, J., Wang, Q., Collins, R.L., Laricchia, K.M., Ganna, A., Birnbaum, D.P., et al. (2020). The mutational constraint spectrum quantified from variation in 141,456 humans. Nature *581*, 434–443. https://doi.org/10.1038/s41586-020-2308-7.

60. Pirastu, N., Cordioli, M., Nandakumar, P., Mignogna, G., Abdellaoui, A., Hollis, B., Kanai, M., Rajagopal, V.M., Parolo, P.D.B., Baya, N., et al. (2021). Genetic analyses identify widespread sex-differential participation bias. Nat. Genet. *53*, 663–671. https://doi.org/10.1038/s41588-021-00846-7.

61. Bissegger, M., Laurentino, T.G., Roesti, M., and Berner, D. (2020). Widespread intersex differentiation across the stickleback genome – the signature of sexually antagonistic selection? Mol. Ecol. *29*, 262–271. https://doi.org/10.1111/mec.15255.

62. Privé, F., Aschard, H., Carmi, S., Folkersen, L., Hoggart, C., O'Reilly, P.F., and Vilhjálmsson, B.J. (2022). Portability of 245 polygenic scores when derived from the UK Biobank and applied to 9 ancestry groups from the same cohort. Am. J. Hum. Genet. *109*, 12–23. https://doi.org/10.1016/j.ajhg.2021.11.008.

63. Traglia, M., Bout, M., and Weiss, L.A. (2022). Sex-heterogeneous SNPs disproportionately influence gene expression and health. PLoS Genet. *18*, e1010147. https://doi.org/10.1371/journal.pgen.1010147.

64. Berg, J.J., Harpak, A., Sinnott-Armstrong, N., Joergensen, A.M., Mostafavi, H., Field, Y., Boyle, E.A., Zhang, X., Racimo, F., Pritchard, J.K., and Coop, G. (2019). Reduced signal for polygenic adaptation of height in UK Biobank. Elife *8*, e39725. https://doi.org/10.7554/eLife.39725.

65. Sohail, M., Maier, R.M., Ganna, A., Bloemendal, A., Martin, A.R., Turchin, M.C., Chiang, C.W., Hirschhorn, J., Daly, M.J., Patterson, N., et al. (2019). Polygenic adaptation on height is overestimated due to uncorrected stratification in genome-wide association studies. Elife *8*, e39702. https://doi.org/10.7554/eLife.39702.

66. Young, A.I., Benonisdottir, S., Przeworski, M., and Kong, A. (2019). Deconstructing the sources of genotype-phenotype associations in humans. Science *365*, 1396–1400. https://doi.org/10.1126/science.aax3710.

67. Coop, G., and Przeworski, M. (2022). Lottery, luck, or legacy. A review of "The Genetic Lottery: why DNA matters for social equality". Evolution *76*, 846–853. https://doi.org/10.1111/evo.14449.

68. Mills, M.C., and Tropf, F.C. (2020). Sociology, genetics, and the coming of age of sociogenomics. Annu. Rev. Sociol. *46*, 553–581. https://doi.org/10.1146/annurev-soc-121919-054756.

69. Coop G. (2019). Reading Tea Leaves? Polygenic Scores and Differences in Traits Among Groups. https://doi.org/10.48550/arXiv.1909.00892.

70. Ober, C., Loisel, D.A., and Gilad, Y. (2008). Sex-specific genetic architecture of human disease. Nat. Rev. Genet. *9*, 911–922. https://doi.org/10.1038/nrg2415.

71. Khan, S.S., Ning, H., Wilkins, J.T., Allen, N., Carnethon, M., Berry, J.D., Sweis, R.N., and Lloyd-Jones, D.M. (2018). Association of body mass index with lifetime risk of cardiovascular disease and compression of morbidity. JAMA Cardiol. *3*, 280–287. https://doi.org/10.1001/jamacardio.2018.0022.

72. Vazquez, G., Duval, S., Jacobs, D.R., and Silventoinen, K. (2007). Comparison of body mass index, waist circumference, and waist/hip ratio in predicting incident diabetes: a meta-analysis. Epidemiol. Rev. *29*, 115–128. https://doi.org/10.1093/epirev/mxm008.

73. Ning, Y., Wang, L., and Giovannucci, E.L. (2010). A quantitative analysis of body mass index and colorectal cancer: findings from 56 observational studies. Obes. Rev. *11*, 19–30. https://doi.org/10.1111/j.1467-789X.2009.00613.x.

74. Brown, C.D., Higgins, M., Donato, K.A., Rohde, F.C., Garrison, R., Obarzanek, E., Ernst, N.D., and Horan, M. (2000). Body mass index and the prevalence of hypertension and dyslipidemia. Obes. Res. *8*, 605–619. https://doi.org/10.1038/oby.2000.79.

75. Arthur, R.S., Dannenberg, A.J., and Rohan, T.E. (2021). The association of prediagnostic circulating levels of cardiometabolic markers, testosterone and sex hormone-binding globulin with risk of breast cancer among normal weight postmenopausal women in the UK Biobank. Int. J. Cancer *149*, 42–57. https://doi.org/10.1002/ijc.33508.

76. Wang, J., Fan, X., Yang, M., Song, M., Wang, K., Giovannucci, E., Ma, H., Jin, G., Hu, Z., Shen, H., and Hang, D. (2021). Sex-specific associations of circulating testosterone levels with all-cause and cause-specific mortality. Eur. J. Endocrinol. *184*, 723–732. https://doi.org/10.1530/EJE-20-1253.

77. Fry, A., Littlejohns, T.J., Sudlow, C., Doherty, N., Adamska, L., Sprosen, T., Collins, R., and Allen, N.E. (2017). Comparison of sociodemographic and health-related characteristics of UK biobank participants with those of the general population. Am. J. Epidemiol. *186*, 1026–1034. https://doi.org/10.1093/aje/kwx246.

78. Bycroft, C., Freeman, C., Petkova, D., Band, G., Elliott, L.T., Sharp, K., Motyer, A., Vukcevic, D., Delaneau, O., O'Connell, J., et al. (2018). The UK Biobank resource with deep phenotyping and genomic data. Nature *562*, 203–209. https://doi.org/10.1038/s41586-018-0579-z.

79. Berisa, T., and Pickrell, J.K. (2016). Approximately independent linkage disequilibrium blocks in human populations. Bioinformatics *32*, 283–285. https://doi.org/10.1093/bioinformatics/btv546.

80. Haworth, S., Mitchell, R., Corbin, L., Wade, K.H., Dudding, T., Budu-Aggrey, A., Carslake, D., Hemani, G., Paternoster, L., Smith, G.D., et al. (2019). Apparent latent structure within the UK Biobank sample has implications for epidemiological analysis. Nat. Commun. *10*, 333. https://doi.org/10.1038/s41467-018-08219-1.

81. Stephens, M. (2017). False discovery rates: a new deal. Biostatistics *18*, 275–294. https://doi.org/10.1093/biostatistics/kxw041.

82. Sudlow, C., Gallacher, J., Allen, N., Beral, V., Burton, P., Danesh, J., Downey, P., Elliott, P., Green, J., Landray, M., et al. (2015). UK biobank: an open access resource for identifying the causes of a wide range of complex diseases of middle and old age. PLoS Med. *12*, e1001779. https://doi.org/10.1371/journal.pmed.1001779.

83. Davies, N.M., Holmes, M. v, and Davey Smith, G. (2018). Reading Mendelian randomisation studies: a guide, glossary, and checklist for clinicians. BMJ *362*, k601. https://doi.org/10.1136/bmj.k601.

84. Smith, G.D., and Ebrahim, S. (2003). 'Mendelian randomization': can genetic epidemiology contribute to understanding environmental determinants of disease? Int. J. Epidemiol. *32*, 1–22. https://doi.org/10.1093/ije/dyg070.

85. Gillespie, J.H. (2004). Population Genetics: A Concise Guide, 2nd ed. (Johns Hopkins University Press).

86. Wang, Y., Guo, J., Ni, G., Yang, J., Visscher, P.M., and Yengo, L. (2020). Theoretical and empirical quantification of the accuracy of polygenic scores in ancestry divergent populations. Nat. Commun. *11*, 3865. https://doi.org/10.1038/s41467-020-17719-y.

STAR★METHODS

KEY RESOURCES TABLE

REAGENT or RESOURCE	SOURCE	IDENTIFIER
Deposited data		
Genotype and phenotype files and minor allele frequencies from UK Biobank	Bycroft et al., 2018[78]	https://www.ukbiobank.ac.uk/
1000 Genomes phase 3 (build 37)	Auton et al., 2015	https://www.cog-genomics.org/plink/2.0/resources#phase3_1kg
Sex-specific summary statistics	This paper	https://doi.org/10.5281/zenodo.7222725
Both-sex summary statistics	This paper	https://doi.org/10.5281/zenodo.7508246
LD Scores and weights	Bulik-Sullivan et al., 2015[36]	https://alkesgroup.broadinstitute.org/LDSCORE/
LD blocks	Berisa and Pickrell, 2016[79]	https://doi.org/10.1093/bioinformatics/btv546
Allele frequency data from gnomAD v3.1.2	Karczewski et al., 2020[59]	https://gnomad.broadinstitute.org/
Software and algorithms		
R	R Core Team	https://www.R-project.org
plink v1.90 beta	Purcell and Chang, 2021	https://www.cog-genomics.org/plink/
Plink v2.00 alpha	Purcell and Chang, 2020	https://www.cog-genomics.org/plink/2.0/
LD Score Regression v1.0.1	Bulik-Sullivan et al. 2015[42]	https://github.com/bulik/ldsc
Ensembl command line variant effect predictor (VEP) v106	McLaren et al., 2016	https://github.com/Ensembl/ensembl-vep.git
mashr: Multivariate Adaptive Shrinkage in R	Urbut et al., 2019[45]	https://github.com/stephenslab/mashr
VCFTools	Danecek et al., 2011	https://vcftools.github.io/downloads.html
BLAST	Camacho et al., 2009	https://blast.ncbi.nlm.nih.gov/Blast.cgi
LiftOver	Kent et al., 2002	https://genome.ucsc.edu/cgi-bin/hgLiftOver
BLAT	Kent, 2002	http://genome.ucsc.edu/cgi-bin/hgBlat
Custom code	This study	https://doi.org/10.5281/zenodo.7765067

RESOURCE AVAILABILITY

Lead contact

Further information and requests for resources should be directed to and will be fulfilled by the lead contact, Arbel Harpak (arbelharpak@utexas.edu).

Materials availability

This study did not generate new unique reagents.

Data and code availability

This study used genotype and phenotype data from the UK Biobank https://www.ukbiobank.ac.uk/.

Sex-specific and additive GWAS summary statistics are available at Zenodo: https://doi.org/10.5281/zenodo.7222725 and https://doi.org/10.5281/zenodo.7508246 respectively, and are publicly available as of the data of publication. DOIs are listed in the key resources table.

All original code has been deposited at https://github.com/harpak-lab/amplification_gxsex and Zenodo: https://doi.org/10.5281/zenodo.7765067, and is publicly available as of the date of publication. DOIs are listed in the key resources table.

Any additional information required to reanalyze the data reported in this paper is available from the lead contact upon request.

METHOD DETAILS

UK Biobank sample characteristics

The UK Biobank is an extensive database that contains a wide variety of phenotypic and genotypic information of around half a million participants aged 40-69 at recruitment.[78]

In this study, we considered 337,111 individuals who passed quality control (QC) checks, which included the removal of samples identified by the UK Biobank with sex chromosome aneuploidy or self-reported sex differing from sex determined from genotyping analysis. We excluded related individuals (3rd-degree relatives or closer) as identified by the UK Biobank in data field 22020. To reduce potential population structure confounding, we further limited our sample to individuals identified by the UK Biobank as "White British" in data field 22006. These are individuals who both self-identified as White and as British and were additionally very tightly clustered in the genetic principal component space.[78,80] Individuals who had withdrawn from the UK Biobank by the time of this study were removed. For each phenotype, we also removed individuals who had missing data for the specified phenotype. These procedures left us with between 255,426 to 336,551 individuals in the analysis for each trait.

Expectations for sex-specific heritabilities with no GxSex

In the section "The limited scope of analyzing GxSex via heritability differences and genetic correlations," we report our observation that, for most traits examined, sex-specific heritabilities (i.e., estimated independently from sex-stratified GWAS) were both higher than the heritability in the combined sample. Here, we explain why this observation is inconsistent with a simple model in which genetic effects are the same across the sexes.

Under a simple additive model of variance in a trait Y within each sex Z,

$$Var[Y|Z] = Var[G|Z] + Var[E|Z], \quad \text{(Equation 2)}$$

where Y, G, E represent the trait value, additive effect, and environmental effect (including all non-genetic context aside from sex), respectively. Under this model, the sex-specific heritability h_z^2 is

$$h_z^2 = \frac{Var[G|Z]}{Var[G|Z] + Var[E|Z]}. \quad \text{(Equation 3)}$$

Therefore, sex differences in heritability are either due to sex differences in genetic variance, in environmental variance, or both. If genetic effects are equal, differences in environmental variance alone could cause heritability differences (Figure 1C, first model). But as we show below, the heritability in the combined sample cannot be smaller than both sex-specific heritabilities.

We assume as before that allele frequencies are highly similar between males and females. Since genetic effects are equal, this implies

$$Var[G|Z = m] \approx Var[G|Z = f].$$

For the environmental variance, we have that

$$Var[E] = \mathbb{E}_Z[Var[E|Z]] + Var_Z[\mathbb{E}[E|Z]] = \mathbb{E}_Z[Var[E|Z]] + 0 = \mathbb{P}(Z = m)Var[E|Z = m] + \mathbb{P}(Z = f)Var[E|Z = f] \leq \max_{z \in \{m,f\}} Var[E|Z = z]. \quad \text{(Equation 4)}$$

The first equality follows from the law of total variance. In the second equality, we have assumed that there are no mean sex differences in the environmental effects (or, in practice in our analysis and as routine in other analyses, that mean phenotypic sex differences have been subtracted out), giving

$$\mathbb{E}[E|Z = m] = \mathbb{E}[E|Z = f] = \mathbb{E}[E].$$

Equation 4 shows that the combined environmental variance cannot be greater than the larger of the two sex-specific environmental variances. It follows that if the genetic variance is equal in both sexes, then the heritability in the combined sample cannot be smaller than both of the sex-specific heritabilities,

$$h^2 = \frac{Var[G]}{Var[G] + Var[E]} \geq \frac{Var[G]}{Var[G] + \max_{z \in \{m,f\}} Var[E|Z]} = \min_{z \in \{m,f\}} h_Z^2. \quad \text{(Equation 5)}$$

Multivariate adaptive shrinkage (mash)

We used multivariate adaptive shrinkage (*mash*) to examine correlation and differences in magnitude of SNP effects between males and females.[45] *mash* is an adaptive shrinkage method[81] that improves upon previous methods of estimating and comparing effects across multiple conditions by flexibly allowing for a mixture of effect covariance patterns between conditions and requiring only summary statistics from each condition (including a point estimate of the effect and corresponding standard error for each SNP and condition). The method adapts to patterns of sparsity, sharing, and correlation among the conditions to compute improved effect estimates.

In this study, we set two conditions, male and female, and provided effect estimates and corresponding standard errors from our male-specific and female-specific GWAS. *mash* learns from the data by estimating mixture proportions of various predefined covariance matrices representing different patterns in effects. Using maximum likelihood, mash assigns low weights to matrices that capture fewer patterns in the data, and higher weights to those that capture more.

Mixture weights for covariance structure between male and female effects

To interpret patterns of SNP effects between males and females, we inputted 66 hypothesis-based covariance matrices (Figure S2) spanning a range of correlations and relative magnitudes of effects between males and females. We used a random subset of all SNPs for mash to learn the covariance mixture weights. In order for the random subset to contain approximately independent SNPs and capture the weight of SNPs with no effect (Figure S2), we created a subset of SNPs for each trait by taking a random SNP from each of 1703 approximately independent LD blocks estimated for Europeans.[79] *mash* can also generate data-driven covariance matrices that capture SNP effects in the data, but we did not use this feature since the data-driven matrices had negligible differences from our hypothesized matrices (in terms of *l* 2 norm) and were less interpretable.

For each trait, we repeat this weight-learning step 100 times, sampling the SNPs from the 1703 LD blocks without replacement to fit the mash model and generate mixture proportions. We then take the average proportion for each covariance matrix as an estimate of its weight, effectively treating each of the 100 samples as i.i.d. draws.

Choice of SNPs used to estimate male-female effect covariance

We examined the effect of using a random subset taken from different p-value thresholds [1, 5e-2, 1e-5, 5e-8] while selecting from LD blocks. By doing so, we can examine differences in the distribution of weights across the p-value thresholds. We performed this test on height, BMI, testosterone, and BMI-adjusted waist:hip ratio. For each trait, weight placed on the no-effect matrix decreased as we reduced the p-value threshold (Figure S4A). Patterns of weights for non-null effect matrices varied across the traits (Figures S4B and S4C). Since *mash* considers the proportion of null effects and sex-specific, SNP-specific noise; together with the fact that for complex traits, less significant associations may still reflect valuable signal, we decided on using the whole set of SNPs to sample from when estimating mixture proportions.

Simulating equal genetic effects and heterogeneous estimation noise among the sexes

To ensure that *mash* was not mistaking sex differences in estimation noise (e.g. via differences in the extent of environmental variance) to be differences in the magnitude of genetic effects, we performed a simulation study. In short, samples of males and females were generated under the model given by Equation 2. Genetic effects were set as equal, but the environmental variance differed among the sexes. We then perform a GWAS on both samples and input the simulated GWAS results into *mash*, and test whether the estimated mixture weights spuriously suggest the presence of GxSex. We performed this simulation on a grid of parameters, including heritabilities in males set to either 5% or 50%, female to male environmental variance ratio of 1, 1.5 or 5; and 100, 1,000 or 10,000 causal SNPs.

First, we created a sample of 300K individuals with randomly assigned sex. We then sampled genotypes for all individuals consisting of 20K SNPs by sampling from the observed distribution of allele frequencies from UK Biobank's imputed data,[82] assuming linkage equilibrium. From the 20K SNPs, we portioned out the predetermined number of causal SNPs and assigned effect sizes by sampling from a Standard Normal distribution. We set the environmental variance for males using the equation

$$Var[E|Z=m] := \frac{Var[G|Z=m]\left(1-h_m^2\right)}{h_m^2} = \frac{\left(\sum_{i=0}\beta_i^2 2p_i(1-p_i)\right)\left(1-h_m^2\right)}{h_m^2} \qquad \text{(Equation 6)}$$

where $Var[E\|Z=m]$ is the simulated environmental variance for males, $G|Z=m$ is the genetic effect in a male, h_m^2 is the heritability in males and β_i and p_i are the effect size and allele frequency at site i, which are equal for males and females. We multiplied $Var[E\|Z=m]$ by the predetermined environmental variance ratio to obtain the environmental variance for females $Var[E\|Z=f]$. Afterwards, for each individual j with sex z_j, we sampled the environmental effect E_j as

$$E_j \sim N(0, Var[E|Z=z_j].$$

Phenotypes were then set using the following additive model,

$$y_j = \sum_{i=0}\beta_i x_{ij} + E_j \qquad \text{(Equation 7)}$$

where y_j is the phenotypic value for individual j and x_{ij} is the number of effect allele copies at the ith causal SNP for the jth individual. With the phenotype, genotype and environmental effect set, we obtained the estimated effect sizes, $\{\hat{\beta}_i\}$, using least squares simple linear regression for all 20K SNPs and used the estimated effect sizes and corresponding standard errors as input into *mash*.

For nearly all parameters, out of the weights on matrices other than the null matrix, the vast majority was placed on the matrix for perfect correlation, equal magnitude (Figure S5). As the number of causal SNPs increased, the weight on the no-effect covariance matrix decreased accordingly. These results suggest that *mash* was not grossly mistaking differences in environmental variance as amplification.

Simulating sex-biased amplification

To evaluate whether *mash* accurately captures sex-biased amplification of genetic effects (a measure we have used in the x-axis of Figures 4A and 4B), we followed the same simulation procedure described in the Section "Simulating equal genetic effects and heterogeneous estimation noise among the sexes". However, instead of using equal genetic effects in males and females, we sampled genetic effects from pre-specified covariance matrices (Figure S6 left-hand panel). We set the female to male environmental variance ratio as 1.2 and the heritability as 0.5. We generated data from (A) a model in which all genetic effects are sampled from a matrix where male and female effects are equal, (B) a model in which 86% of the genetic effects are sampled from a matrix where effects between the sexes are equal, and 14% of the effects are sampled from a matrix where the female effect size magnitude is 4 times that of males, and (C) a model in which 86% of effects are sampled from a matrix where effects between sexes are equal, and 14% of effects are sampled from a matrix of only female-specific effects. After simulating sex-specific GWAS on the three models, we input the results into *mash* to estimate mixture weights. We repeated this simulation procedure 100 times for each model.

For model (A), the equal effect matrix received 78% of the weight, and the difference between male-larger and female-larger magnitude was 1% (Figure S6). For model (B), 67% of the weight was placed on the matrix for equal effects. The weight difference between male-larger and female-larger magnitude was 13%. In model (C), 69% of the weight was on the matrix for equal effects, and the difference between male-larger and female-larger magnitude was 16%. These simulation results therefore suggest some overestimation of the proportion of SNPs with magnitude differences. However, the measure of "sex-biased amplification" matched that of the pre-specified generative models up to an error of 2%. Therefore, the simulations suggest that "sex-biased amplification" is measured accurately in our estimation procedure.

Testosterone as an amplifier

We tested a model of testosterone as a modulator of magnitude differences in males and females. We first split individuals by sex and for each sex, created 10 bins of testosterone levels. We adjusted one of the 10 bins to have testosterone levels overlap between males and females. The overlapping testosterone bin was based on fewer individuals (~800) compared to the other bins (~2200). For each trait, each of the sexes, and within each bin, we performed a simple linear regression of trait values to the PGS for the trait (using a PGS based on both-sex summary statistics (supplemental information)). We interpret the estimated coefficient for the effect of the PGS as a proxy for the magnitude of polygenic effect. Finally, we summarized the relationship between testosterone level and magnitude of polygenic effect across bins using the Pearson correlation between the two.

To mitigate the possible effects of confounding (of testosterone and magnitude of polygenic effect) or reverse causation (the magnitude of polygenic effect on the focal trait causally affecting testosterone levels) we employed a version of Mendelian Randomization[83,84] of the same analysis (Figure S15). Namely, we replaced testosterone levels of each individual with their PGS for testosterone. Here, given the near-zero genetic correlation between males and females, we used our sex-specific PGS for each sex; otherwise, the analysis is unchanged.

We also examined whether participants' age may have confounded the relationship between testosterone and polygenic effect. In this analysis, instead of using the polygenic effect as the response variable across bins, we used the polygenic effect residualized for mean age in the bin and examined the effect of an individual's polygenic score on the residual (Figure S16).

Model of shared amplification

Here, we suggest a null model in which amplification is shared between genetic and environmental effects. We then suggest a prediction that the model yields and explain how we tested this prediction across traits (Figure 6).

If an amplifier is shared, it may be modeled as having the same scalar multiplier effect on genetic and environmental effects. Consider the within-sex additive model of Equation 1 in the section "The limited scope of analyzing GxSex via heritability differences and genetic correlations" above. For a phenotype value Y_z in sex $z \in \{m, f\}$

$$Y_z = c + G_z + E_z, \tag{Equation 8}$$

Where c is a constant, E_z is the environmental effect and

$$G_z = \sum_{\text{site } i} x_i \beta_i^z \tag{Equation 9}$$

is the polygenic effect where β_i^z is the effect of an allele at site i (say the minor allele) in sex Z and x_i is the number of copies of the allele. We assume here for simplicity that male genetic effects relate to female effects solely through a shared polygenic amplification constant, α,

$$\beta_i^m = \alpha \beta_i^f \;\forall i; \quad \alpha > 0. \tag{Equation 10}$$

Allele frequencies are once again assumed to be close to equal between males and females, since due to random segregation of alleles during meiosis, genotype frequencies at autosomal sites are independent of sex; and further assuming no substantial interaction between genotype and sex affecting participation in UKB.[43] Consequently, differences in polygenic effect distributions between males and females are solely based on GxSex, and thus:

$$Var[G_m] = \alpha^2 Var[G_f]. \qquad \text{(Equation 11)}$$

The model we would like to test is one where the amplification of environmental effects can also be simplified to the same scalar multiplier,

$$E_m = \alpha E_f, \text{and } Var[E_m] = \alpha^2 Var[E_f]. \qquad \text{(Equation 12)}$$

Hence, with equal amplification,

$$\frac{Var[G_m]}{Var[G_f]} = \frac{Var[E_m]}{Var[E_f]} \qquad \text{(Equation 13)}$$

To test the model of shared amplification between environmental and polygenic effects (Equation 8) we obtained the genetic and environmental variance for males and females based on the following relationships,

$$Var[G_z] = h^2 Var[Y_z] \qquad \text{(Equation 14)}$$

and

$$Var[E_z] = (1 - h^2) Var[Y_z], \qquad \text{(Equation 15)}$$

where $Var[G_z]$, $Var[E_z]$, and $Var[G_z]$ are the additive genetic, environmental, and phenotype variances, respectively. Estimates of the sex-specific heritabilities, h_z^2, were obtained from previous estimates using LD Score Regression (supplemental information).

Representing male genetic or environmental variance as x, and the corresponding female variance as y, we derived standard errors for the ratio of male to female variance using the 2nd-order Taylor approximation for the standard error of a ratio of estimators of x and y,

$$SE\left[\frac{\hat{x}}{\hat{y}}\right] = \sqrt{Var\left[\frac{\hat{x}}{\hat{y}}\right]} \cong \frac{E[\hat{x}]}{E[\hat{y}]}\sqrt{\frac{Var[\hat{x}]}{E[\hat{x}]^2} + \frac{Var[\hat{y}]}{E[\hat{y}]^2} - \frac{2Cov[\hat{x},\hat{y}]}{E[\hat{x}]E[\hat{y}]}} \approx \frac{\hat{x}}{\hat{y}}\sqrt{\frac{SE[\hat{x}]^2}{\hat{x}^2} + \frac{SE[\hat{y}]^2}{\hat{y}^2}} \qquad \text{(Equation 16)}$$

assuming independence between $\hat{x}$ and $\hat{y}$ since they are statistics of independent sampling distributions (independent samples of males and females). The standard errors of the genetic and environmental variance were estimated using the law of total variance for a product of two random variables. For $\hat{a}$ and $\hat{b}$, unbiased estimators of the two parameters a and b, respectively, we get

$$SE[\hat{a}\hat{b}] = \sqrt{SE[\hat{a}]^2 SE[\hat{b}]^2 + E[\hat{a}]^2 SE[\hat{b}]^2 + E[\hat{b}]^2 SE[\hat{a}]^2}.$$

Plugging in the point estimate $\hat{a}$ for $E[\hat{a}] = a$ and the point estimate $\hat{b}$ for $E[\hat{b}] = b$,

$$\widehat{SE}[\hat{a}\hat{b}] = \sqrt{SE[\hat{a}]^2 SE[\hat{b}]^2 + \hat{a}^2 SE[\hat{b}]^2 + \hat{b}^2 SE[\hat{a}]^2}. \qquad \text{(Equation 17)}$$

In this case, a represents the phenotypic variance for a sex, $Var[Y_z]$, and b represents either h_z^2 for estimation of genetic variance or $(1 - h_z^2)$ for estimation of environmental variance. Lastly, to obtain the standard error of the phenotypic variance, we used 100 bootstrapped samples $Var[Y_z]_i$ of estimates of the phenotypic variance in sex z,

$$\widehat{SE}[\hat{a}] = \sqrt{\frac{\sum_{i=1}^{100}\left(Var[Y_z]_i - \overline{Var[Y_z]_j}\right)^2}{100 - 1}}$$

Finally, for each trait, we estimated $\tilde{Z}$, the ratio of the two male-female ratios (environmental and genetic, y and x axes in Figure 6, respectively), and its standard error, $SE[\tilde{Z}]$, using the same method as in Equation 16. Under the null hypothesis of equal environmental and genetic amplification (Equation 8),

$$H_0 : E[Z] = 0, \qquad \text{(Equation 18)}$$

where

$$Z = \frac{\tilde{Z} - 1}{SE[\tilde{Z}]}.$$

In Figure 6, we approximated 90% confidence intervals on Z by treating it as a Z score, i.e., further treating Z as a Standard Normal.

A model of sexually antagonistic selection

We developed a model relating sex differences in additive effects on a trait at a biallelic locus (β_m and β_f) and divergence in allele frequencies. Our model resembles that of Cheng and Kirkpatrick[14] who developed a similar model relating allele-frequency differences and sex bias in gene expression. In short, we modeled sexually antagonistic, post-conception viability selection on a focal complex trait. We assumed allele frequencies in adult males, p_m, and adult females, p_f, are at equilibrium, i.e. do not change in consecutive generations. Under these conditions, we derive the relationship

$$F_{ST} \approx AV_{GxSex},$$

where F_{ST}[57] is the fixation index with respect to the male and female subpopulations, i.e., the proportion of heterozygosity in the population that is due to allelic divergence between the sexes. V_{GxSex} is defined as

$$V_{GxSex} := 2p(1-p)(\beta_m - \beta_f)^2, \quad \text{(Equation 19)}$$

where p is the allele frequency in zygotes. A is a parameter that, importantly, is shared across all variants affecting the trait and can be thought of as the intensity of sexually antagonistic selection acting on genetic variation for the trait in question.

In our model, allele frequencies at the autosomal locus are assumed to be equal in males and female zygotes. F_{ST} at adulthood takes the form

$$F_{ST} := \frac{Var_z[p_z]}{\overline{p}(1-\overline{p})} = \frac{E[p_z^2] - \overline{p}^2}{\overline{p}(1-\overline{p})} = \frac{p_m^2 + p_f^2 - \left(\frac{p_m+p_f}{2}\right)^2}{\overline{p}(1-\overline{p})} = \frac{(p_m - p_f)^2}{4\overline{p}(1-\overline{p})}, \quad \text{(Equation 20)}$$

where

$$\overline{p} = \frac{p_m + p_f}{2}.$$

If we further assume a near-1:1 sex ratio such that $\overline{p} \approx p$,

$$F_{ST} \approx \frac{(p_m - p_f)^2}{4p(1-p)}. \quad \text{(Equation 21)}$$

Sexually antagonistic selection acting on viability will cause divergence in allele frequencies between adult males and females. We write the relative viabilities of the homozygote for the reference allele, the heterozygote and the homozygote for the effect allele as $1::1+d_zS_z::1+S_z$ for each sex $z \in \{m,f\}$. The selection coefficient S_z and dominance coefficient d_z can be frequency-dependent, in which case these coefficients take their values at equilibrium. We can write the additive selection coefficient of the effect allele as

$$s_z = [p + (1-2p)d_z]S_z. \quad \text{(Equation 22)}$$

Assuming that zygotes are at Hardy-Weinberg equilibrium, the allele frequency in each sex at adulthood is

$$p_z \approx p + p(1-p)s_z, \quad \text{(Equation 23)}$$

where we neglected terms of order s_z^2[85]. Plugging Equation 23 into Equation 21, the divergence between males and females post-selection is

$$F_{ST} \approx \frac{1}{4}p(1-p)(s_m - s_f)^2. \quad \text{(Equation 24)}$$

We model the strength of viability selection acting on males and females as linear with the additive effect on a focal trait in each sex,

$$s_z = a_z\beta_z, \quad \text{(Equation 25)}$$

and recalling the simplifying assumption that allele frequencies are at equilibrium under sexually antagonistic viability selection at the locus, such that selection favoring an allele in one sex is balanced by selection against that allele in the other sex,

$$s_f = -s_m. \quad \text{(Equation 26)}$$

If $\beta_m = \beta_f$, then Equation 24 simplifies to

$$F_{ST} \approx p(1-p)(a_f\beta_f)^2 = \frac{a_f^2}{2}V_G. \quad \text{(Equation 27)}$$

where

$$V_G = 2p(1-p)\beta_f^2. \quad \text{(Equation 28)}$$

is the additive genetic variance. However, when β_m does not strictly equal β_f, Equation 25, 26 together imply

$$\beta_m + \beta_f = \frac{\beta_m + \beta_f}{\beta_m - \beta_f}(\beta_m - \beta_f) = \frac{\frac{s_m}{a_m} - \frac{s_m}{a_f}}{\frac{s_m}{a_m} + \frac{s_m}{a_f}}(\beta_m - \beta_f) = \frac{a_f - a_m}{a_f + a_m}(\beta_m - \beta_f). \quad \text{(Equation 29)}$$

Finally, using Equation 25,

$$s_m - s_f = a_m\beta_m - a_f\beta_f = \frac{1}{2}[(a_m + a_f)(\beta_m - \beta_f) + (a_m - a_f)(\beta_m + \beta_f)], \quad \text{(Equation 30)}$$

which together with Equation 29 gives

$$s_m - s_f = \frac{1}{2}\left[(a_m + a_f) + \frac{(a_m - a_f)(a_f - a_m)}{a_f + a_m}\right](\beta_m - \beta_f) = \frac{2a_m a_f}{a_m + a_f}(\beta_m - \beta_f). \quad \text{(Equation 31)}$$

We denote the heritability due to GxSex at the locus as $V_{GxSex} := 2p(1-p)(\beta_m - \beta_f)^2$ and the parameter relating this contribution to the differentiation in allele frequencies as

$$A: = 2\left(\frac{a_m a_f}{a_m + a_f}\right)^2, \quad \text{(Equation 32)}$$

and plugging Equation 31 into Equation 24, we get

$$F_{ST} \approx AV_{GxSex}. \quad \text{(Equation 33)}$$

as given by Equation 1 in the section "sexually antagonistic selection."

Estimating the potential for sexually antagonistic selection on standing variation (A)

For each trait and gnomAD subsample (supplemental information), we estimated A using weighted least squares linear regression of our estimate of F_{ST} ($\widehat{F_{ST}}$) to our estimate of V_{GxSex} ($\widehat{V}_{GxSex}$), with weight w inversely proportional to our site-specific estimate of noise in the estimate of V_{GxSex},

$$w = \frac{1}{\widehat{Var[\widehat{V}_{GxSex}]}}. \quad \text{(Equation 34)}$$

To simplify the estimation of $Var[\widehat{V}_{GxSex}]$, we treated the allele frequency p as perfectly estimated, and as independent of the allele frequency in the GWAS sample—as different data are used in the GWAS (UK Biobank) and in the allele frequency estimation (gnomAD). Under these assumptions,

$$Var[\widehat{V}_{GxSex}] = Var\left[2p(1-p)\widehat{D}^2\right] = [2p(1-p)]^2 Var\left[(\widehat{\beta}_m - \widehat{\beta}_f)^2\right], \quad \text{(Equation 35)}$$

and thus the task at hand is estimating $Var[(\widehat{\beta}_m - \widehat{\beta}_f)^2]$. Using the law of total variance,

$$Var\left[(\widehat{\beta}_m - \widehat{\beta}_f)^2\right] = Var_{\widehat{\beta}_f}\left[E_{\widehat{\beta}_m}\left[(\widehat{\beta}_m - \widehat{\beta}_f)^2 \middle| \widehat{\beta}_f\right]\right] + E_{\widehat{\beta}_f}\left[Var_{\widehat{\beta}_m}\left[(\widehat{\beta}_m - \widehat{\beta}_f)^2 \middle| \widehat{\beta}_f\right]\right]. \quad \text{(Equation 36)}$$

We begin with the argument of the first term,

$$E_{\widehat{\beta}_m}\left[(\widehat{\beta}_m - \widehat{\beta}_f)^2 \middle| \widehat{\beta}_f\right] = E_{\widehat{\beta}_m}\left[\widehat{\beta}_m^2 - 2\widehat{\beta}_m\widehat{\beta}_f + \widehat{\beta}_f^2 \middle| \widehat{\beta}_f\right] = \mu_m^2 + \sigma_m^2 - 2\mu_m\widehat{\beta}_f + \widehat{\beta}_f^2, \quad \text{(Equation 37)}$$

where we denote

$$\mu_z = E[\widehat{\beta}_z];\ \sigma_z^2 = Var[\widehat{\beta}_z] \quad \text{(Equation 38)}$$

for each sex $z \in \{m, f\}$. Plugging Equation 37 into the first term of Equation 36,

$$\begin{aligned} Var_{\widehat{\beta}_f}\left[E_{\widehat{\beta}_m}\left[(\widehat{\beta}_m - \widehat{\beta}_f)^2 \middle| \widehat{\beta}_f\right]\right] &= Var_{\widehat{\beta}_f}\left[\mu_m^2 + \sigma_m^2\right] + Var_{\widehat{\beta}_f}\left[\widehat{\beta}_f^2 - 2\mu_m\widehat{\beta}_f\right] \\ &= 0 + Var_{\widehat{\beta}_f}\left[\widehat{\beta}_f^2 - 2\mu_m\widehat{\beta}_f\right] = Var_{\widehat{\beta}_f}\left[\widehat{\beta}_f^2\right] + 4Var_{\widehat{\beta}_f}\left[\mu_m\widehat{\beta}_f\right] - 4\mu_m Cov_{\widehat{\beta}_f}\left[\widehat{\beta}_f^2, \widehat{\beta}_f\right], \end{aligned} \quad \text{(Equation 39)}$$

where the first and second step follow from the fact that $\mu_m^2 + \sigma_m^2$ is a constant. We can take note of the fact that $\widehat{\beta}_z$ is Normally distributed around β_z, and in particular that it has no skewness. Therefore,

$$Cov_{\widehat{\beta}_z}\left[\widehat{\beta}_z^2, \widehat{\beta}_z\right] = E\left[\widehat{\beta}_z^3\right] - E[\widehat{\beta}_z]E\left[\widehat{\beta}_z^2\right] = (\mu_z^3 + 3\mu_z\sigma_z^2 + \gamma_z\sigma_z^3) - \mu_z(\mu_z^2 + \sigma_z^2) = 2\mu_z\sigma_z^2, \quad \text{(Equation 40)}$$

where $\gamma_z = 0$ is the skewness of $\widehat{\beta}_z$. We can also note that

$$Var_{\widehat{\beta}_z}\left[\widehat{\beta}_z^2\right] = Var_{\widehat{\beta}_z}\left[(\sigma_z b_z + \mu_z)^2\right], \quad \text{(Equation 41)}$$

where we defined

$$b_z = \frac{\widehat{\beta}_z - \mu_z}{\sigma_z},$$

and therefore b_z is a Standard Normal and therefore b_z^2 is Chi-squared with one degree of freedom. Equation 41 now gives

$$\begin{aligned} Var_{\widehat{\beta}_z}\left[\widehat{\beta}_z^2\right] &= Var_{\widehat{\beta}_z}\left[\sigma_z^2 b_z^2 + 2\sigma_z\mu_z b_z\right] \\ &= Var_{\widehat{\beta}_z}\left[\sigma_z^2 b_z^2\right] + Var[2\sigma_z\mu_z b_z] + Cov\left[\sigma_z^2 b_z^2, 2\sigma_z\mu_z b_z\right] \\ &= Var\left[b_z^2\right]\sigma_z^4 + 4Var[b_z]\mu_z^2\sigma_z^2 + 0 = 2\sigma_z^4 + 4\mu_z^2\sigma_z^2. \end{aligned} \quad \text{(Equation 42)}$$

Plugging Equations 40 and 42 into Equation 39, we find

$$Var_{\widehat{\beta}_f}\left[E_{\widehat{\beta}_m}\left[(\widehat{\beta}_m - \widehat{\beta}_f)^2\middle|\widehat{\beta}_f\right]\right] = 2\sigma_f^4 + 4\mu_f^2\sigma_f^2 + 4\mu_m^2\sigma_f^2 - 8\mu_m\mu_f\sigma_f^2. \quad \text{(Equation 43)}$$

We now turn to the second term of Equation 36. First,

$$\begin{aligned} Var_{\widehat{\beta}_m}\left[(\widehat{\beta}_m - \widehat{\beta}_f)^2\middle|\widehat{\beta}_f\right] &= Var\left[\widehat{\beta}_m^2 + 2\widehat{\beta}_m\widehat{\beta}_f\middle|\widehat{\beta}_f\right] \\ &= Var\left[\widehat{\beta}_m^2\right] + 4\sigma_m^2\widehat{\beta}_f^2 - 4\widehat{\beta}_f Cov\left[\widehat{\beta}_m, \widehat{\beta}_m^2\right]. \end{aligned} \quad \text{(Equation 44)}$$

Equations 40 and 42 again give us

$$Var_{\widehat{\beta}_m}\left[(\widehat{\beta}_m - \widehat{\beta}_f)^2\middle|\widehat{\beta}_f\right] = 2\sigma_m^4 + 4\mu_m^2\sigma_m^2 + 4\sigma_m^2\widehat{\beta}_f^2 - 8\mu_m\sigma_m^2\widehat{\beta}_f, \quad \text{(Equation 45)}$$

which then gives

$$E_{\widehat{\beta}_f}\left[Var_{\widehat{\beta}_m}\left[(\widehat{\beta}_m - \widehat{\beta}_f)^2\middle|\widehat{\beta}_f\right]\right] = 2\sigma_m^4 + 4\mu_m^2\sigma_m^2 + 4\sigma_m^2\left(\sigma_f^2 + \mu_f^2\right) - 8\mu_m\mu_f\sigma_m^2. \quad \text{(Equation 46)}$$

Plugging Equations 43 and 46 into Equation 36, we obtain

$$\begin{aligned} &Var\left[(\widehat{\beta}_m - \widehat{\beta}_f)^2\right] = \\ &= 2\left(\sigma_m^4 + \sigma_f^4\right) + 4\sigma_m^2\sigma_f^2 + 4\left(\mu_m^2\sigma_m^2 + \mu_f^2\sigma_f^2\right) + 4\left(\sigma_m^2\mu_f^2 + \sigma_f^2\mu_m^2\right) - 8\mu_m\mu_f\left(\sigma_m^2 + \sigma_f^2\right). \end{aligned} \quad \text{(Equation 47)}$$

Finally, we estimate μ_z with the GWAS-derived point estimate of the effect $\widehat{\beta}_z$ and σ_z with its standard error, $\widehat{\sigma}_z = [\widehat{\beta}_z]$. Plugging back into Equation 35, we obtain

$$Var\left[\widehat{V}_{GxSex}\right] = [2p(1-p)]^2\left[2\left(\widehat{\sigma}_m^4 + \widehat{\sigma}_f^4\right) + 4\widehat{\sigma}_m^2\widehat{\sigma}_f^2 + 4\left(\widehat{\beta}_m^2\sigma_m^2 + \widehat{\beta}_f^2\sigma_f^2\right) + 4\left(\widehat{\sigma}_m^2\widehat{\beta}_f^2 + \widehat{\sigma}_f^2\widehat{\beta}_m^2\right) - 8\widehat{\beta}_m\widehat{\beta}_f\left(\widehat{\sigma}_m^2 + \widehat{\sigma}_f^2\right)\right]. \quad \text{(Equation 48)}$$

Using Equation 33, we estimate F_{st} with the estimator

$$\widehat{F_{st}} = \frac{n_{st}}{d_{st}}, \quad \text{(Equation 49)}$$

where

$$n_{st} = (\widehat{p_m} - \widehat{p}_f)^2 - SE(\widehat{p_m})^2 - SE(\widehat{p}_f)^2, \quad \text{(Equation 50)}$$

$$d_{st} = 4\widehat{p}(1-\widehat{p}) - SE(\widehat{p_m})^2 - SE(\widehat{p}_f)^2,$$

and noting that

$$E\left[\widehat{F_{st}}\right] \approx \frac{E[n_{st}]}{E[d_{st}]} = \frac{(p_m - p_f)^2 - Var(p_m) + Var(p_f) + E\left\{SE(\widehat{p_m})^2\right] + E\left[SE(\widehat{p}_f)^2\right]}{4p(1-p) + Var(p_m)^2 + Var(p_f)^2 - E\left\{SE(\widehat{p_m})^2\right] - E\left[SE(\widehat{p}_f)^2\right]} = F_{st}, \quad \text{(Equation 51)}$$

where in the first equality we approximated the expectation of a ratio with the ratio of expectations. Therefore, Equation 49 provides an approximately unbiased estimator of F_{st} despite the absence of genotype frequencies.

To perform this estimation of A on the GWAS and F_{st} data, we used paired v and V_{GxSex} points for all sites which passed all previous stages of filtering. Weights were set by Equation 34 and follow Equation 48 where $\widehat{\beta}_m$ and $\widehat{\beta}_f$ are the GWAS effect estimates as above, and $\widehat{\sigma}_m$ and $\widehat{\sigma}_f$ are the GWAS standard errors (SE) estimates for the effect size of each site per trait.

To minimize the possibility of LD between sites used in the analysis as much as possible, we used the approximately independent LD blocks in Europeans[79] as in Section "Mixture weights for covariance structure between male and female effects". Namely, we subdivided the genome into 1703 approximately independent LD blocks as before. We iterated over the 1703 blocks and sampling one site per block in a given iteration, using a sample of (up to) 1703 post-filtering sites to perform the weighted linear regression of F_{ST} on $V_{G\times Sex}$. The slope of this regression was used as an estimate of A. We perform this estimation procedure 1,000 times and take an average of Z scores (slope point estimates divided by their SE) as the final estimate of A. In each replicate, we sample with replacement m LD blocks from the m LD blocks which had at least one site within them post-filtering (supplemental information); we then sample one site per resampled block. In Figure 7D, each point is the mean of the 1,000 samples of one site per LD block and 90% confidence intervals show the range between the 5th and 95th percentile of 10,000 bootstrap re-samplings of 1,000 samples, calculating a new mean for each bootstrap.

In the main text, we focus on the results performed this estimation for Ashkenazi Jewish, Finnish, and Non-Finnish European populations as the other ancestry group-stratified subsamples in *gnomAD* are further diverged from the UKB White British sample and therefore our GWAS estimates are expected to be less portable.[62,86] We also performed a similar analysis using UKB data to measure differentiation in allele frequencies between males and females, rather than an independent dataset (*gnomAD*) as in the main text. Since individual level data was available in this case, we replaced F_{st} with L_{ST}, a measure developed by Ruzicka et al.[56] L_{st} can be thought of as site-specific F_{st} controlled for major axes of population structure differentiating males and females (Figure S20).

Cell Genomics, Volume *3*

Supplemental information

Amplification is the primary mode of gene-by-sex interaction in complex human traits

Carrie Zhu, Matthew J. Ming, Jared M. Cole, Michael D. Edge, Mark Kirkpatrick, and Arbel Harpak

Supplementary Materials for: Amplification is the Primary Mode of Gene-by-Sex Interaction in Complex Human Traits

Carrie Zhu[1,2], Matthew J. Ming[1,2], Jared M. Cole[1,2], Michael D. Edge[3], Mark Kirkpatrick[2], Arbel Harpak[1,2,+]
[1] Department of Population Health, The University of Texas at Austin, Austin, TX
[2] Department of Integrative Biology, The University of Texas at Austin, Austin, TX
[3] Department of Quantitative and Computational Biology, University of Southern California, Los Angeles, CA
[+] Lead Contact: arbelharpak@utexas.edu

Table of Contents

Supplemental Methods 4
- Text S1: Genotype data 4
- Text S2: Phenotype data 4
- Text S3: GWAS 4
- Text S4: Miami plots 4
- Text S5: Heritability and genetic correlation estimation using LD Score Regression 5
- Text S6: Qualitative differences between our conclusions and approaches based on independent analysis of individual sex-heterogenous SNPs 5
- Text S7: Posterior estimates of sex-specific effect sizes 6
- Text S8: Improved utility of sex-specific models for complex phenotype prediction. 7
- Text S9: Data Filtering for Sexually-Antagonistic Selection Analysis. 9
- Text S10: Estimating Male-Female F_{ST} and V_{GxSex} 10
- Text S11: Competing models for sex differences in trait variance 11

Supplementary Figures 13
- Figure S1. Sex-specific Miami plots, related to Figure 3 13
- Figure S2. All hypothesis covariance matrices, related to Figure 3 14
- Figure S3 Inferring polygenic covariance structure, related to Figure 3 15
- Figure S4 Testing p-value thresholds for input into *mash*, related to Figure 3 17
- Figure S5: Simulation of sex-invariant genetic effects but with variable environmental variance, related to Figure 3 19
- Figure S6: Simulation of covariance structure, related to Figure 3 21
- Figure S7: Proportion of non-trivial weights on hypothesis matrices, related to Figure 3 and Figure 4 22
- Figure S8: Across traits, phenotypic variance sex ratio correlates with the phenotypic mean sex ratio, related to Figure 4 23
- Figure S9: Process of generating posterior estimates from *mash*, related to Figure 3 24

Figure S10: Process of comparing models for complex phenotype prediction, related to Figure 3 25
Figure S11: Evaluating evidence for systematic amplification, related to Figure 2 27
Figure S12: The predictive utility of G-by-Sex aware polygenic scores, related to Figure 3 29
Figure S13: Genetic effect and testosterone levels – using additive model PGS, related to Figure 5 31
Figure S14: Genetic effect and testosterone levels – using sex-specific model PGS, related to Figure 5 33
Figure S15: Genetic effect and PGS for testosterone, related to Figure 5 34
Figure S16: Genetic effect residualized for age and testosterone levels, related to Figure 5 36
Figure S17: Example of shared polygenic and environmental amplification in muscle, related to Figure 6 37
Figure S18: Competing models for sex differences in trait variance, related to Figure 6 38
Figure S19: Z-scores for strength of sexually-antagonistic selection, related to Figure 7 40
Figure S20: Z-scores using UK Biobank data, related to Figure 7 41
Supplementary Tables 42
Table S1: Filtering site with small sample sizes, related to Figure 7 42
Table S2. Filtering monoallelic sites, related to Figure 7 43
Table S3: Filtering sites that were not overlapping between the gnomAD dataset and the GWAS dataset, related to Figure 7 44
Table S4: Filtering by p-value threshold, related to Figure 7 45
Table S5: Identification of SNPs susceptible to mis-mapping to sex chromosomes— Kasimatis et al., related to Figure 7 46
Table S6: Characterizing GxSex based on independent analysis of individual sex-heterogenous SNPs, related to Figure 3 47
Supplementary Data 48
Data S1-S27: Mixture weights on hypothesis covariance matrices, related to Figure 3 48
Data S1: Albumin 49
Data S2: Arm fat-free mass (L) 50
Data S3: Arm fat-free mass (R) 51
Data S4: BMI 52
Data S5: Calcium 53
Data S6: Creatinine 54
Data S7: Diastolic blood pressure 55
Data S8: Eosinophil percentage 56
Data S9: Forced vital capacity 57

Data S10: HbA1c 58
Data S11: Height 59
Data S12: Hip circumference 60
Data S13: IGF-1 61
Data S14: Lymphocyte percentage 62
Data S15: Total protein 63
Data S16: Pulse rate 64
Data S17: Red blood cell count 65
Data S18: SHBG 66
Data S19: Systolic blood pressure 67
Data S20: Testosterone 68
Data S21: Urate 69
Data S22: Urea 70
Data S23: Waist circumference 71
Data S24: Waist to hip ratio 72
Data S25: Weight 73
Data S26: Whole body fat mass 74
Data S27: Waist:hip (BMI adjusted) 75
References 76

Supplemental Methods

Text S1: Genotype data

We focused on autosomal bi-allelic SNPs with an INFO score greater than 0.8. Using *plink 2.0 alpha*[1–6], we further retained variants with calling rate > 0.95, Hardy-Weinberg equilibrium test p-value > 10-9, minor allele frequency (MAF) > 0.001 and MAF < 0.999, following a QC procedure used by the Neale Lab on version 2 of UK Biobank[7]. These were computed on the aforementioned quality-controlled sample set (**Methods "UK Biobank sample characteristics"**) and resulted in 9,607,691 SNPs.

Text S2: Phenotype data

Our analysis consisted of 27 continuous traits for their relatively high SNP heritability estimates, based on LD Score regression[7,8]. BMI-adjusted waist:hip ratio (WHR) was calculated by regressing WHR on BMI and obtaining the residuals, as obtained using the following commands in R:

```
model <- lm(<WHR>~<BMI>, data=<dataframe>)
residuals <- summary(model)$residuals
```

Text S3: GWAS

We performed all GWAS using *plink 2.0 alpha*, adjusting for birth year, sex, and the first 10 principal components (PC) provided by the UK Biobank as covariates. Covariates were standardized to mean 0, variance 1 (using the flag --covar-variance-standardize). We generated sex-specific GWAS summary statistics for each trait by separating the sample by males and females and applying the same regression model for each sex independently. Any variants with missing values in the summary statistics were removed from further analysis.

Text S4: Miami plots

We used the *Ensembl Variant Effect Predictor (VEP)*[9] based on the GRCh38 genome build to annotate SNPs with p-value < 5×10^{-8} in Miami plots (**Figs S1**). Using the --nearest flag, we retrieved the gene with the closest protein-coding transcription start site within 5,000 bp up- and down-stream each SNP.

Text S5: Heritability and genetic correlation estimation using LD Score Regression

We estimated the SNP heritability of each trait for both-sex, female-specific, and male-specific GWAS, as well as the genetic correlation between sexes using LD Score Regression[8,10]. Since our GWAS summary statistics were based on "White British" individuals of primarily European ancestry, we used the precalculated LD scores computed by Bulik-Sullivan et al.[8].

Text S6: Qualitative differences between our conclusions and approaches based on independent analysis of individual sex-heterogenous SNPs

A common approach for detecting and characterizing GxSex based on sex-stratified GWAS data is to test the hypothesis of sex differences in genetic effects at each site independently. As an example, Traglia et al.[11] meta-analyzed sex-stratified GWAS from various sources, many of which standardized effects within-sex. Traglia et al. defined sex-heterogeneous ("sex-het") SNPs as ones where a t-test testing a null hypothesis of equal effects in males and females was significant at a level of 0.05. They then characterized the pervasiveness of different modes of GxSex focusing on this subset. In particular, they categorized sex-het SNPs where the marginal association p-value was significant at a level of 0.05 as "having an effect in only one sex". They then categorized the remaining sex-het SNPs as having opposite signs of effects in the two sexes or the same sign and different magnitudes. With these categorizations at hand, they argued that the vast majority of sex-het SNPs have an effect in only one sex.

In this study, we analyzed the polygenic covariance of genetic effects genome-wide, rather than focusing on significant individual SNPs. We proposed that sex differences were largely due to differences in magnitude rather than opposite or sex-private effects. Here, we show that the seemingly discrepant arguments may be a direct result of the different analysis approaches. We show that when we generate data from a generative model adhering to pervasive amplification and use Traglia's et al.'s approach to characterize GxSex based on the simulated data, their classification suggests the same qualitative result—that GxSex manifests primarily via effects that are private to one of the sexes.

We performed a simulation study following a procedure similar to that in the section "**Simulating equal genetic effects and heterogenous estimation noise among sexes**" of the main text. Here, however, we sample genetic effects using the covariance mixtures estimated for five traits – height, BMI, creatinine, IGF-1 and systolic blood pressure (**Data S11, 4, 6, 13, and 19**

respectively). We set the female to male environmental variance ratio as 1.2 and the heritability as 0.05. We simulated genotypes and phenotypic values, and then standardized phenotypic values within-sex and performed a sex-stratified GWAS. Using the effect estimates and corresponding standard errors for males and females, we calculated a t-statistic for each SNP, where

$$t = \frac{\hat{\beta}_{female} - \hat{\beta}_{male}}{\sqrt{SE_{female}^2 + SE_{male}^2}},$$

and identified SNPs with p-value < 0.05 as sex-het SNPs. We further categorized sex-het SNPs as having an effect in only one sex if the effect was only significant in one sex (t-test p-value < 0.05). For SNPs where the sex-specific effect was significant for both sexes or for neither, the SNPs were categorized based on if the effects were of opposite or same sign.

For data generated based on the covariance structures, we indeed find that the classification of sex-het SNPs qualitatively mirrors that of Traglia et al., with the majority of the sex het SNPs categorized as having an effect in just one sex (**Table S6**). Importantly, this is despite having simulated traits with pervasive differences in the magnitude of effects. We therefore conclude that such an approach, including the within-sex standardization of effect sizes (which heavily dilutes signals of pervasive magnitude differences), the ascertainment bias resulting from a focus on individually-significant sex-heterogenous SNPs, and the use of the above-described classification result in mis-characterization of the mode of GxSex, can lead to the mischaracterization of GxSex in complex traits.

Text S7: Posterior estimates of sex-specific effect sizes

mash can also apply adaptive shrinkage after learning patterns in effect sizes to improve marginal point estimates—estimated using the posterior mode—and measures of significance. These posterior estimates could reduce noise by shrinking effects towards zero and possibly reveal greater or lesser variation in effect sizes between males and females. Therefore, using the average of the fitted mixture model over 100 repetitions (**Methods "Mixture weights for covariance structure between male and female effects"**), we computed posterior estimates for each trait be used in further analysis (**Text S8**). *mash* calculates a local false sign rate (lfsr) for each effect, which is similar to a local false discovery rate and is defined as the cumulative density of the posterior distribution of values with a sign that differs from that of the posterior

mean. We mapped lfsr to “pseudo p-values” by ranking the SNPs according to lfsr values, and then ascribing a p-value of the same rank (**Fig. S10**).

Text S8: Improved utility of sex-specific models for complex phenotype prediction.

The pervasiveness of GxSex that we infer, alongside the mixture of covariance relationships across the genome for each trait, may mean that GxSex is important to consider in phenotypic prediction. To test this possibility, we compared the prediction accuracy of four polygenic scores (PGS) for various traits, together with covariates (**Fig. S12**):

1. An additive PGS, assuming no GxSex, based on both-sex GWAS summary statistics
2. An additive PGS, standardized by sex, based on both-sex GWAS summary statistics
3. A sex-specific, but polygenic covariance-naïve PGS, based on stratified GWAS, fit independently for each sex.
4. A sex-specific, covariance-aware PGS based on posterior sex-specific effect estimates (**Text S7**)

The estimation, prediction and evaluation pipeline are illustrated in **Fig. S10**, and, for models (a) and (b), parallels the procedure described by Choi[11].

First, for each phenotype, we split the sample of unrelated “White British” individuals into a test set of 25K individuals randomly sampled from each sex and a training set with the remaining individuals. Second, we re-ran GWAS on the training set following the same procedure above (**Text S3**), generating both-sex, female-specific, and male-specific summary statistics. For the additive model standardized by sex, we used phenotype values standardized within-sex before performing GWAS to get both-sex summary statistics. Third, to obtain posterior estimates, we first input the effect sizes and standard errors from the male-specific and female-specific GWAS, and took the average of 100 resampling estimates of mixture proportion vectors estimated from a random subset of SNPs with p-value < 1e-5 available in each of 1703 LD blocks[12]. We used the 1e-5 p-value threshold to create the random subset to provide stronger signals and patterns for *mash* to learn from. We then feed estimated mixture proportion vectors into *mash* to perform the refined, covariance-aware estimation of effects. The output from *mash* included posterior mean and a local false sign rate (lfsr) for each SNP. We sorted SNPs by lfsr and matched it to a ranked list of p-values from the additive GWAS summary statistics to create a “pseudo p value” which we use later for thresholding.

Afterwards, we performed clumping separately for the additive both-sex, additive both-sex standardized by sex, male-specific additive, female-specific additive, male covariance-aware and female covariance aware models to produce a subset of significant SNPs that are approximately independent using *plink 1.9 beta*'s --clump command, removing SNPs with pairwise LD threshold $r^2 > 0.1$ or within 250kb. To estimate pairwise LD values, we used a sample of 187 unrelated individuals (population codes GBR and CEU) from 1000 Genomes phase 3[13].

Using the resulting subset of SNPs, we estimated PGS for the individuals in the test set by summing the number of effect alleles an individual has weighted by the allelic effect sizes. We used *plink 2.0 alpha's* --score command along with the --q-score-range flag to repeat the PGS computation over a range of p-value thresholds [1, 0.01, 1e-5, 1e-8]. Therefore, a total of 24 PGS runs were performed for each trait over the combination of six models and four p-value thresholds (**Fig. S10**).

Finally, to assess the prediction accuracy, we computed R^2 for the following,

$$y \sim birth\ year + sex + principal\ componenets + (sex \times M) + ((1 - sex) \times F)$$

$$sex: \{0(female), 1(male)\}$$

$$M: male\ PGS \qquad F: female\ PGS$$

The covariates used for the regression are the same as those used in our GWAS: sex, birth year, and the first 10 PCs of the genotype matrix (UKB data field 22009). We performed regressions both separately by sex and together with both sexes in the test set. For each of the four predictors—additive model, additive standardized by sex model, sex-specific additive model, and sex-specific polygenic covariance-aware model—we selected the p-value threshold with the greatest R^2. We also calculated the incremental R^2, which is the increment in R^2 after adding the PGS to a null model with only the covariates (**Fig. S10**).

We performed 20-fold cross validation for our PGS procedure. Each prediction was performed on a different randomly sampled 5K female and 5K male test set, with the remaining used as the training set. For each model, we averaged the greatest incremental R^2 from the p-value thresholds over the twenty folds for comparison across all models.

The sex-specific, covariance-aware PGS model outperformed the additive model (including model standardized by sex) for 20/27 traits (**Fig. S12**). The sex-specific, covariance-aware PGS model outperformed the sex-specific, covariance-naïve model for all traits. The additive model also outperformed the sex-specific covariance-naïve for all traits but testosterone.

The additive model using phenotype values standardized within sex only outperformed the base additive model for 11/27 traits.

The increase of prediction accuracy in the additive model over the sex-specific covariance-naïve model may be due the additive model being estimated on nearly twice the sample size as the sex-specific models. We chose not to equalize the sample sizes to align the comparison to what would be used in practice. However, despite the disadvantage in decreased sample size, the covariance-aware PGS model outperformed the additive model in approximately 3/4 of the total traits. Interestingly, the additive model standardized by sex did not outperform the base additive model for many traits, including those showing evident sexual dimorphism such as testosterone. These findings support the argument for characterizing GxSex using multiple covariance structures rather than broadly applying within-sex standardization, which can weaken amplification signals and sex differences in the data.

Polygenic scores used in **Methods "Testosterone as an amplifier"** and **Fig. 2** were based on a 5-fold cross validation with 50K individuals in the test set (25K males and 25K females). The polygenic scores were gathered from the first fold for each trait.

Text S9: Data Filtering for Sexually-Antagonistic Selection Analysis.

We downloaded allele frequency data from the gnomAD dataset[14]. Specifically, we used gnomAD v3.1.2, which consists of 76,156 whole genome sequences mapped to the GRCh38 reference. In order to allow this assembly to conform to the GRCh37 build used in the UK Biobank GWAS, we used the UCSC in-browser tool LiftOver[15] (https://genome.ucsc.edu/cgi- bin/hgLiftOver) to identify and convert the GRCh38 sites of interest to their corresponding GRCh37 positions. Allele count summaries are available for the total sample, as well as stratified by sex chromosomes karyotype and genetic ancestry groupings: African/African American samples (abbreviated "afr" in the gnomAD files); Amish ("ami"); Latino/Admixed American ("amr"); Ashkenazi Jewish ("asj"); East Asian ("eas"); Finnish ("fin"); Non-Finnish European ("nfe"); Middle Eastern ("mid"); South Asian ("sas"); and samples not assigned to any population are designated Other ("oth"). Aneuploid individuals (e.g., X or XXY) are not included in the dataset. For the purposes of this study, we again refer to XX as female and XY as male. Total numbers of individuals sampled can be found on the gnomAD website's help page (https://gnomad.broadinstitute.org/help).[14]

We downloaded gnomAD VCF files from the gnomAD browser for all autosomes and used VCFTools[16] to parse the file. We filtered the data to exclude insertions or deletions, and only kept

bi-allelic SNPs. We further removed missing data (3,698 sites). These filtering steps resulted in 2,285,169 remaining sites. In an effort to avoid confounding results that could arise from population substructure, we split the data into the different ancestry groups labeled by gnomAD and worked with the data in each subpopulation separately from this point forward. We removed sites with less than 1,000 alleles in each ancestry group independently. The number of sites we removed at this step depended on the sample size of each group (**Table S1**). This step resulted in the complete removal of the Amish and Middle Eastern subsamples because their low sample sizes.

Finally, we filtered out sites where difference in male and female allele counts may be partly or fully driven by the mismapping of autosomal reads to sex chromosomes or vice-versa[17,18]. We follow a similar approach to Kasimatis et al. to identify such sites[18]. In particular, for every SNP, we extract the 301bp sequence surrounding the SNP, with the SNP's position at the center, from the GRCh37 genome assembly[19]. We further shorten the 301bp sequence into three 150bp-long subsequences with the SNP's position at the center, start or end of the sequence. We then use Mega-BLAST through NCBI's command-line BLAST tool[20] to search for regions of high sequence homology to either of the three subsequences. If any of the three was found to have a 90% or greater sequence identity to a sequence on a sex chromosome, we filtered out the site. This filtering was performed agnostic of ancestry.

We qualitatively compared our list of filtered SNPs to Kasimatis et al.[18]. We based our comparison on sites considered in both works. In particular, we limited the comparison to the genotype array sites, as they did not consider UKB imputed genotypes, and further only to sites we had not removed in a previous filtering step. In terms of parameters, our 90% homology threshold is the same as Kasimatis et al., but we diverge from their algorithm in testing three different sequences per SNP; and also in using shorter sequences (150bp) to model for the mapping of short reads rather than the hybridization of probes. In general, our approach tended to identify more sites than Kasimatis et al. as invalid for analysis (**Table S5**).

Text S10: Estimating Male-Female F_{ST} and V_{GxSex}

We estimated Male-Female F_{ST} for every site remaining in our dataset based on the new estimator we propose in **Eq. 51,** based on the sample allele frequencies. We removed sites for which there were no alternative allele calls for either males or females. This step resulted in a significant

reduction in sample size by one or two orders of magnitude, depending on the ancestry (**Table S2**). We further removed sites that were not found in both the gnomAD dataset and the UKB GWAS dataset. The number of sites removed through this step varied greatly across ancestry groups (**Table S3**).

We used the point estimates and the standard errors of the sex-stratified GWAS (**Table S6)** for 27 physiological or physical traits. We further filtered sites by GWAS p-value. We used four different p-value thresholds at 10^{-3}, 10^{-5}, 10^{-8} and 1 (i.e., all SNPs; see **Table S4** for the number of sites remaining for each p-value threshold). In the main text, we focus on the 10^{-5} threshold, reasoning that it strikes a reasonable, albeit arbitrary, middle ground between sample size and noise. Results for other p-value thresholds are shown in **Fig. S19**.

Finally, we obtained an estimate of V_{GxSex} using **Eq. 19**, where β_m^2 and β_f^2 were estimated using the squared GWAS effect estimates, and p is the total alternate allele frequency as above. For detail on the estimation of the sampling error of V_{GxSex}, see "**Estimating the potential for sexually-antagonistic selection on standing variation (A)**" in the main text.

Text S11: Competing models for sex differences in trait variance

In the main text section "**Are polygenic and environmental effects jointly amplified**", we show that under a model where amplification is pervasive and shared between environmental and genetic effects, we expect the male-female ratio of environmental variance to equal the male-female ratio of genetic variance. Here, we compare the expectation under the pervasive, joint amplification model with expectations under two other longstanding models.

As recently discussed by Zajitschek et al.[21] , the "estrus-mediated variability" hypothesis predicts that females will display higher trait variability in traits affected by the estrous cycle. If we interpret estrus-mediated effects as environmental, this hypothesis suggests equal genetic variance but larger environmental variance in females (**Fig. S18A**; orange line in **Fig. S18B**).

The "greater male variability" hypothesis predicts that males will display higher trait variability. There are multiple rationales offered for this hypothesis, such as stronger sexual selection on males that leads to higher variability via group selection[22–24]. A more widely applicable rationale for this prediction is that mammalian males, as the heterogametic sex, will experience more variable X chromosome effects, whereas these effects will be "averaged out" through heterogeneous X inactivation in females[23]. This hypothesis therefore predicts greater genetic

variance in males and equal environmental variance between males and females (**Fig. S18A**; green line in **Fig. S18B**.

Supplementary Figures

Figure S1. Sex-specific Miami plots, related to Figure 3

“Miami plots”, contrasting statistical significance of marginal effects in a female-specific GWAS (top) and a male-specific GWAS (bottom), for each of the 27 traits analyzed. Data points are thinned out by random selection over multiple levels of p-value thresholds. For larger p-values, more points are not shown.

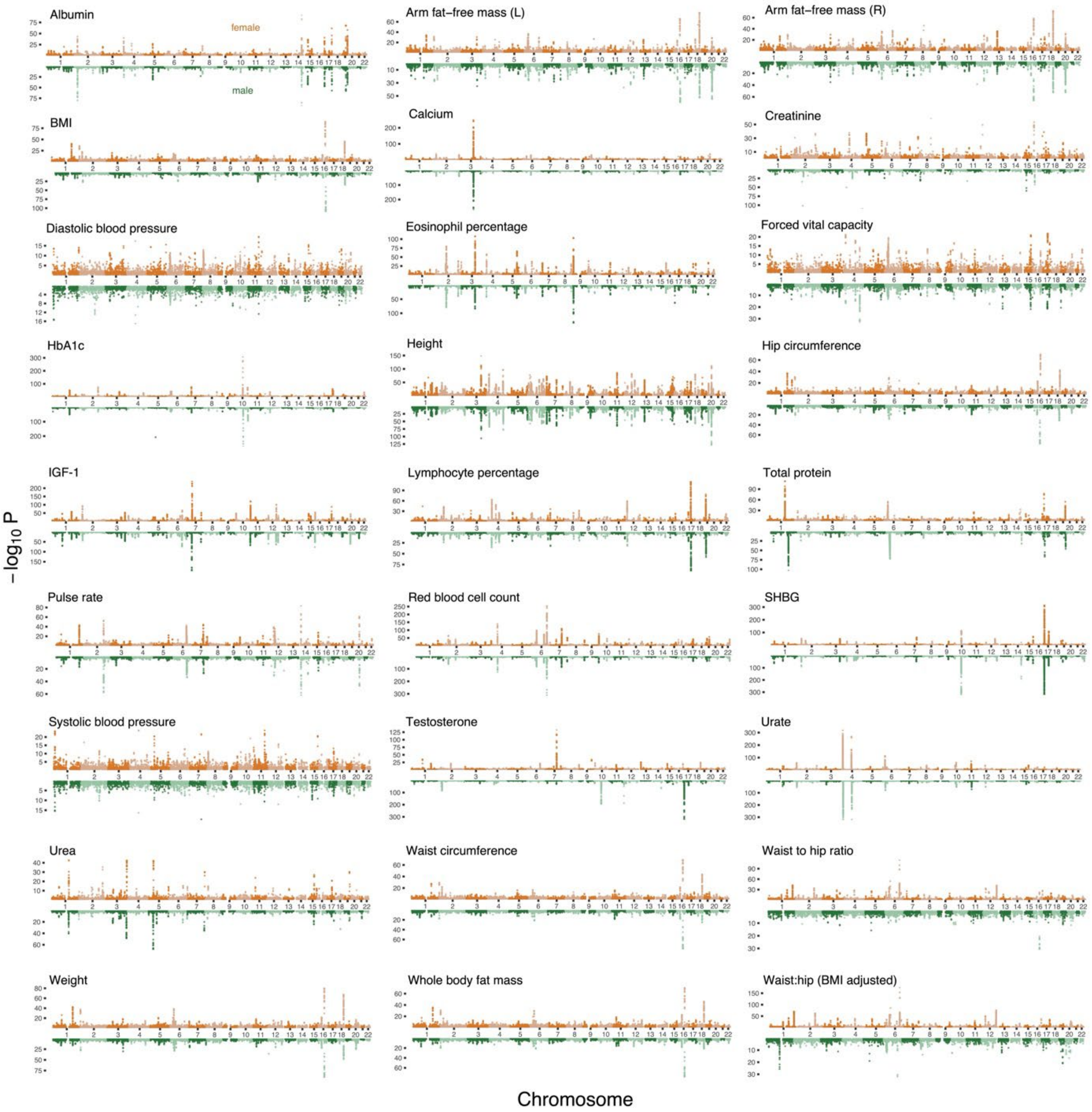

Figure S2. All hypothesis covariance matrices, related to Figure 3

All hypothesis matrices we inputted into *mash* are shown here organized by correlation and magnitude of effect size. The placement of the matrices parallels in the weights in **Data S1-27**.

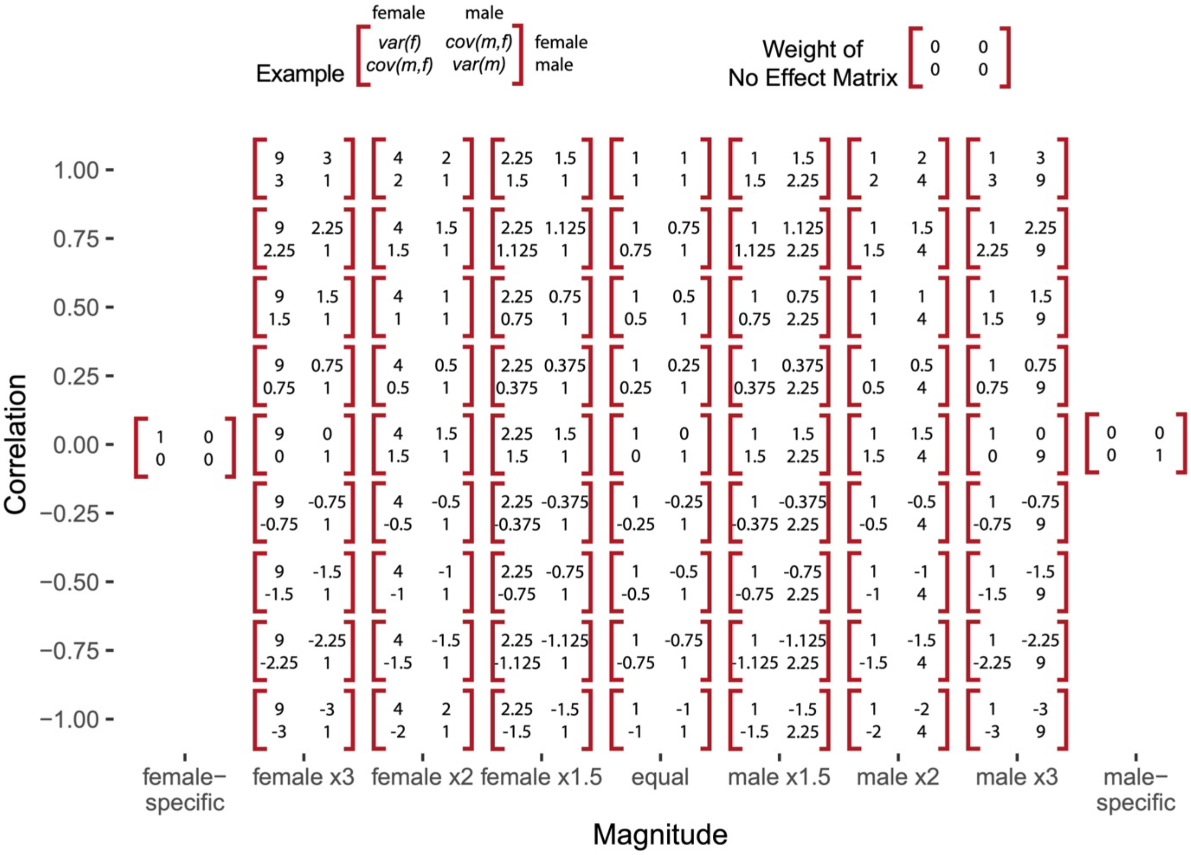

Figure S3 Inferring polygenic covariance structure, related to Figure 3

(A) Our analysis of the polygenic covariance between males and females is based on sex stratified GWAS. Shown for illustration, is a “Miami plot” for testosterone. Highly associated SNPs that are less than 5 kbp away from a transcription start site were annotated with the gene corresponding to the closest one. **(B)** We modelled the sex-stratified GWAS estimates as sampled with error from true effects arising from a mixture of pre-specified hypothesis covariance relationships between female and male genetic effects; see examples in red frames. Each box specifies a mixture weight (±SE) that we infer for one hypothesis matrix. The weight, also indicated by the shade, corresponds to the relative frequency that the specified hypothesis matrix is represented by the variants. The axes state the relative magnitude (amplification) and correlation between males and females, which jointly make up the covariance relationship. **(C)** The x and y axes are a condensed version of the x and y axes from (B) for testosterone. The weights are a proportion of the non-null weights, i.e., the weight divided by sum of all weights except for the weight on the no effect matrix, corresponding to no effects in either sex. For example, the square in purple sums over all 12 weights for matrices corresponding to larger effects on testosterone in males that are negatively correlated with effects in females, 5.1%, divided by the total weight on matrices with nonzero effects, 32%.

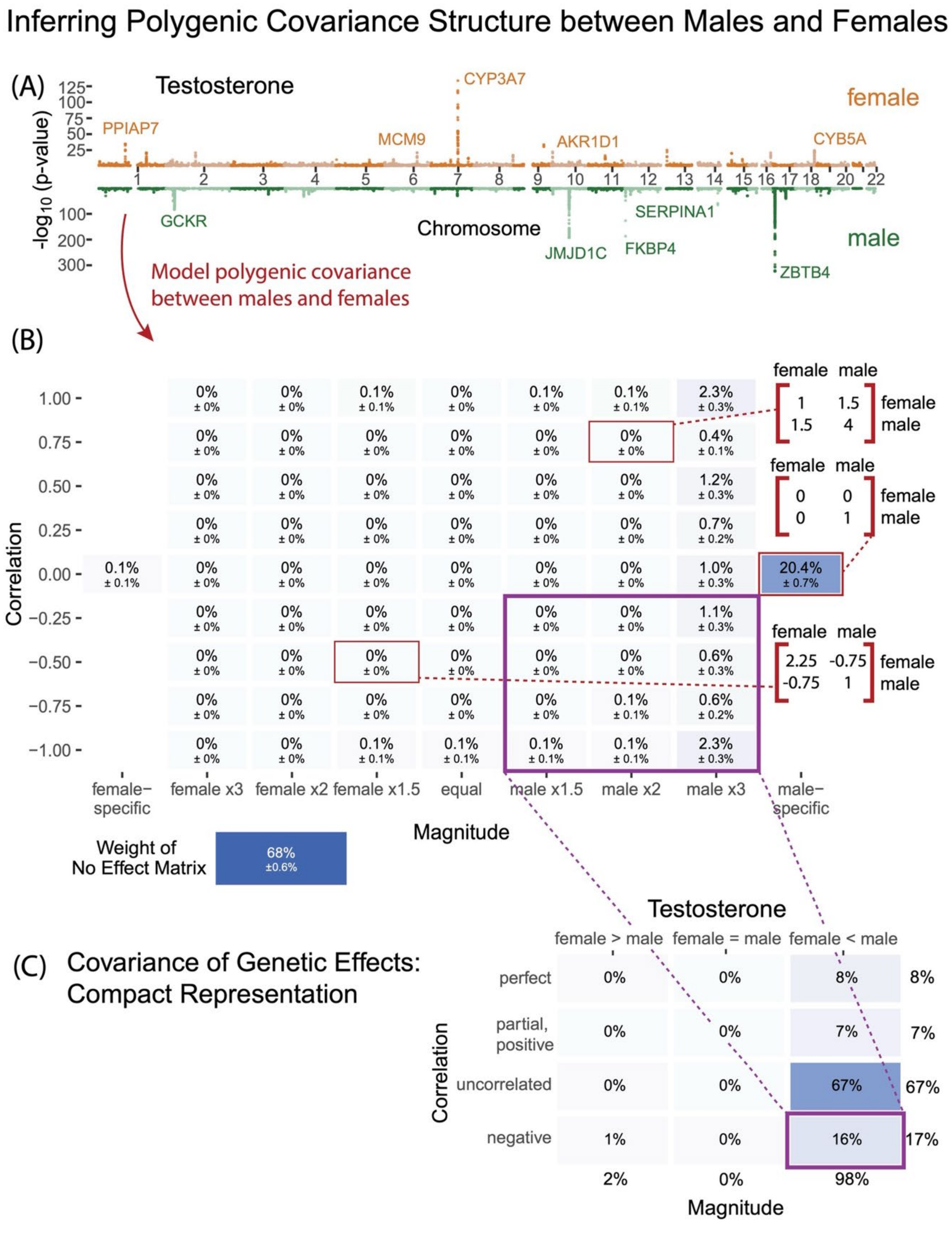
Inferring Polygenic Covariance Structure between Males and Females
(A)
Testosterone
-log10 (p-value)
female
male
Chromosome
PPIAP7
CYP3A7
MCM9
AKR1D1
CYB5A
GCKR
JMJD1C
SERPINA1
FKBP4
ZBTB4
Model polygenic covariance between males and females
(B)
Correlation
Magnitude
female−specific
female x3
female x2
female x1.5
equal
male x1.5
male x2
male x3
male−specific
20.4% ± 0.7%
Weight of No Effect Matrix
68% ±0.6%
(C) Covariance of Genetic Effects: Compact Representation
Testosterone
female > male
female = male
female < male
perfect
partial, positive
uncorrelated
negative

Figure S4 Testing p-value thresholds for input into *mash*, related to Figure 3

We tested the effect of inputting random subsets taken from various p-value thresholds [1, 5e-2, 1e-5, 5e-8] in the *mash* fitting step to estimate mixture proportions. The random subsets were drawn by sampling from the 1703 LD blocks (**Methods "Mixture weights for covariance structure between male and female effects"**). **(A)** Both depict the weight on the no-effect matrix per p-value threshold for four different traits. The left plot shows results from sampling once from each available LD block as long as there were still p-values below the specified threshold. For the right plot, we keep sampling from LD blocks until we reach a subset of 1703 SNPs, ensuring equal sample sizes across the thresholds. **(B)** The percentage of weight by correlation of effects are depicted across the thresholds. Plots in the box show results from equal sample sizes. **(C)** The percentage of weight on different types of magnitude of effects are shown for four different traits across the threshold. Plots in boxes also show results from equal sample sizes.

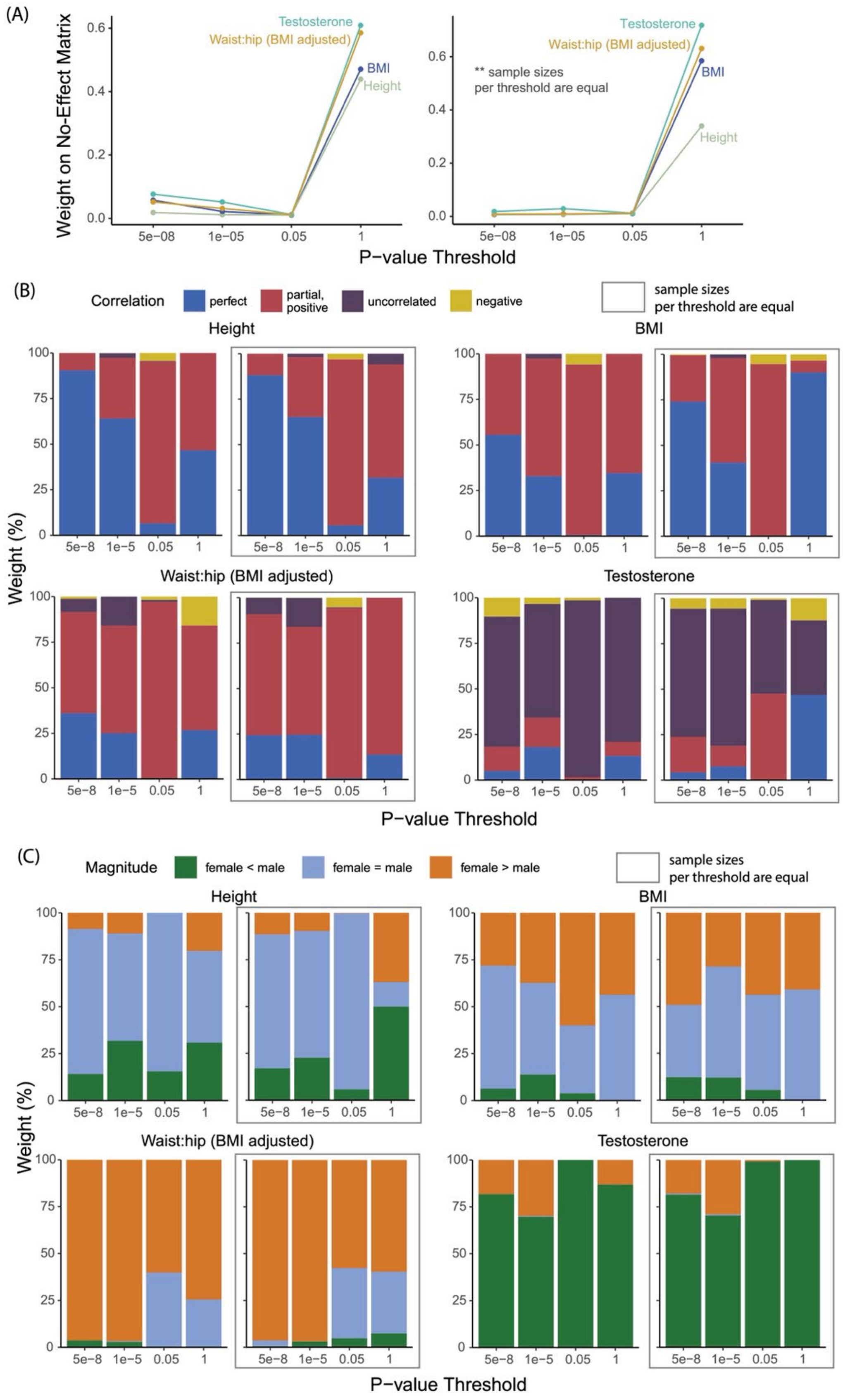
(A)
Weight on No-Effect Matrix
Testosterone
Waist:hip (BMI adjusted)
BMI
Height
** sample sizes per threshold are equal
5e−08 1e−05 0.05 1
P−value Threshold
(B)
Correlation
perfect
partial, positive
uncorrelated
negative
sample sizes per threshold are equal
Height
BMI
Waist:hip (BMI adjusted)
Testosterone
Weight (%)
5e−8 1e−5 0.05 1
P−value Threshold
(C)
Magnitude
female < male
female = male
female > male
sample sizes per threshold are equal
Height
BMI
Waist:hip (BMI adjusted)
Testosterone
Weight (%)
5e−8 1e−5 0.05 1
P−value Threshold

Figure S5: Simulation of sex-invariant genetic effects but with variable environmental variance, related to Figure 3

Mixture weights based on various parameters for number of causal SNPs, male-specific heritability, and female to male environmental variance ratio are shown for all hypothesis covariance matrices represented by the axes. Results are generated from a simulation study based on **Eq. 2**, in which environmental variance differs between the sexes while genetic effects stay the same to test whether *mash* interprets the environmental variance difference as GxSex (**Methods "Environmental variance simulation for *mash*"**). We tested three parameters for female to male environmental variance ratio [1, 1.5, 5]. Parameters for the number of causal SNPs [100, 1K, 10K] and male heritability [0.01, 0.1, 0.5] are listed on the top right.

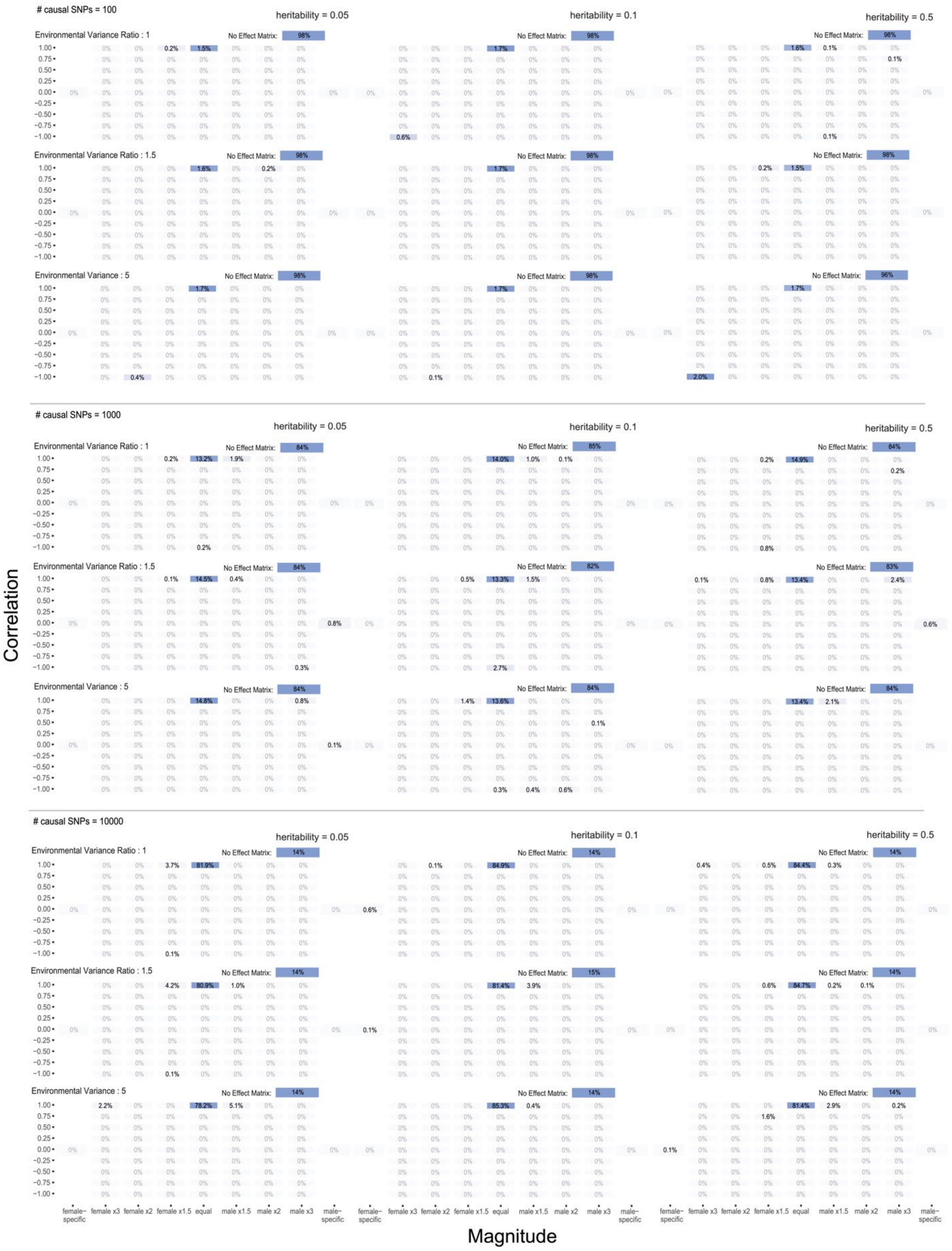

# causal SNPs = 100
# causal SNPs = 1000
# causal SNPs = 10000
heritability = 0.05
heritability = 0.1
heritability = 0.5
Environmental Variance Ratio : 1
Environmental Variance Ratio : 1.5
Environmental Variance : 5
No Effect Matrix:
Correlation
Magnitude
female-specific
female x3
female x2
female x1.5
equal
male x1.5
male x2
male x3
male-specific

Figure S6: Simulation of covariance structure, related to Figure 3

We compared the distribution of mixture weights estimated in *mash* when inputting effect estimates and standard errors are sampled from a pre-specified variance-covariance matrix. The matrices and associated proportions sampled from a particular matrix is shown on the left. We set the female to male environmental variance ratio as 1.2:1 and the heritability to 0.5. **(A)** We simulate a null model in which all effect estimates are drawn from a matrix representing equal effects between males and females. **(B)** 86% of the true effects have equal effects in males and females and 14% are sampled from a relationship in which effects in females are perfectly correlated but have twice the variance of that in males. **(C)** 86% of the true effects have equal effects in males and females and 14% are sampled from a matrix corresponding to female-private effects.

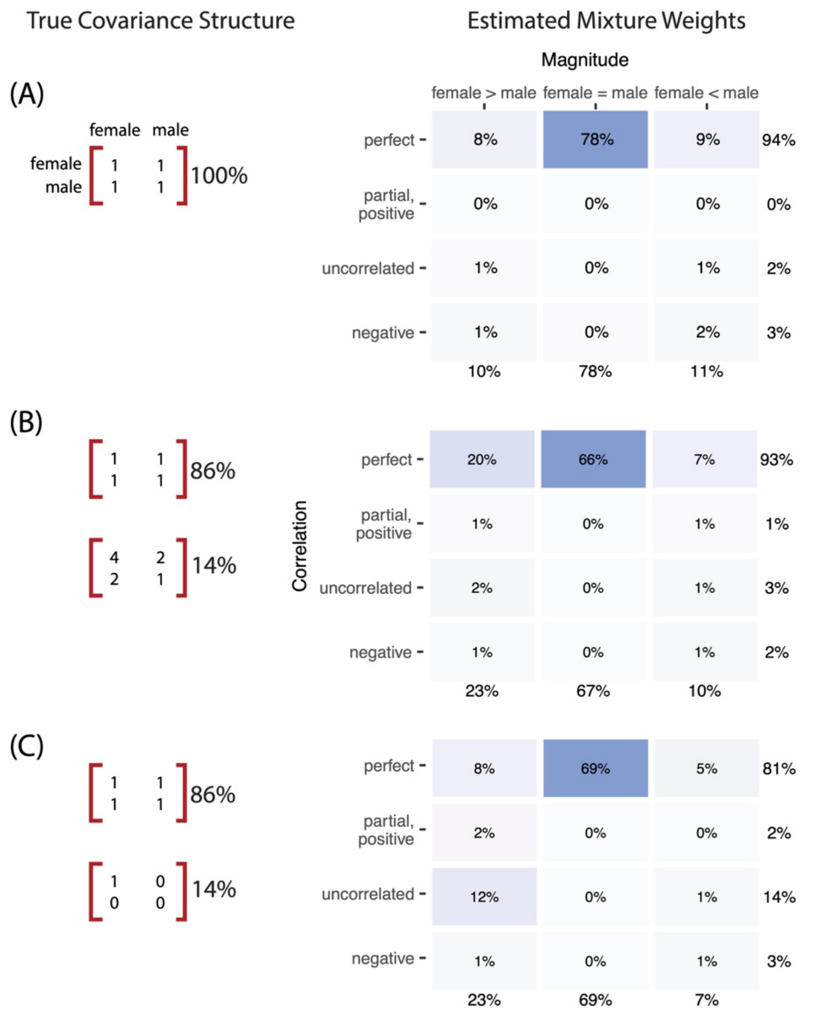

Figure S7: Proportion of non-trivial weights on hypothesis matrices, related to Figure 3 and Figure 4

We examine the proportion of weight on non-trivial hypothesis matrices across 27 complex traits. Here, we define non-trivial as matrices with correlation < 1 or female ≠ male magnitude. The heritability ratio represented by point size is estimated by taking the larger of the two sex-specific heritabilities and dividing it by the smaller. We refer to the 1:1 line to see if more traits are represented more by unequal magnitude or imperfect correlation.

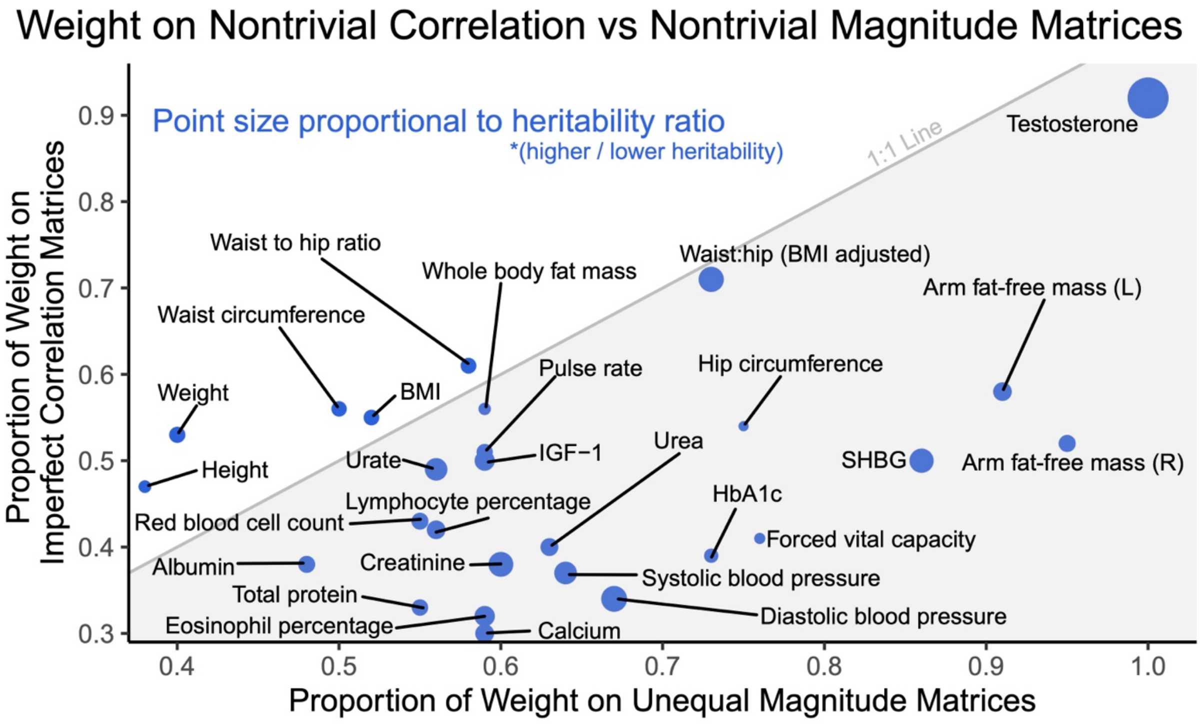

Figure S8: Across traits, phenotypic variance sex ratio correlates with the phenotypic mean sex ratio, related to Figure 4

The solid gray line shows a linear fit, excluding two outliers: testosterone and waist:hip ratio adjusted for BMI.

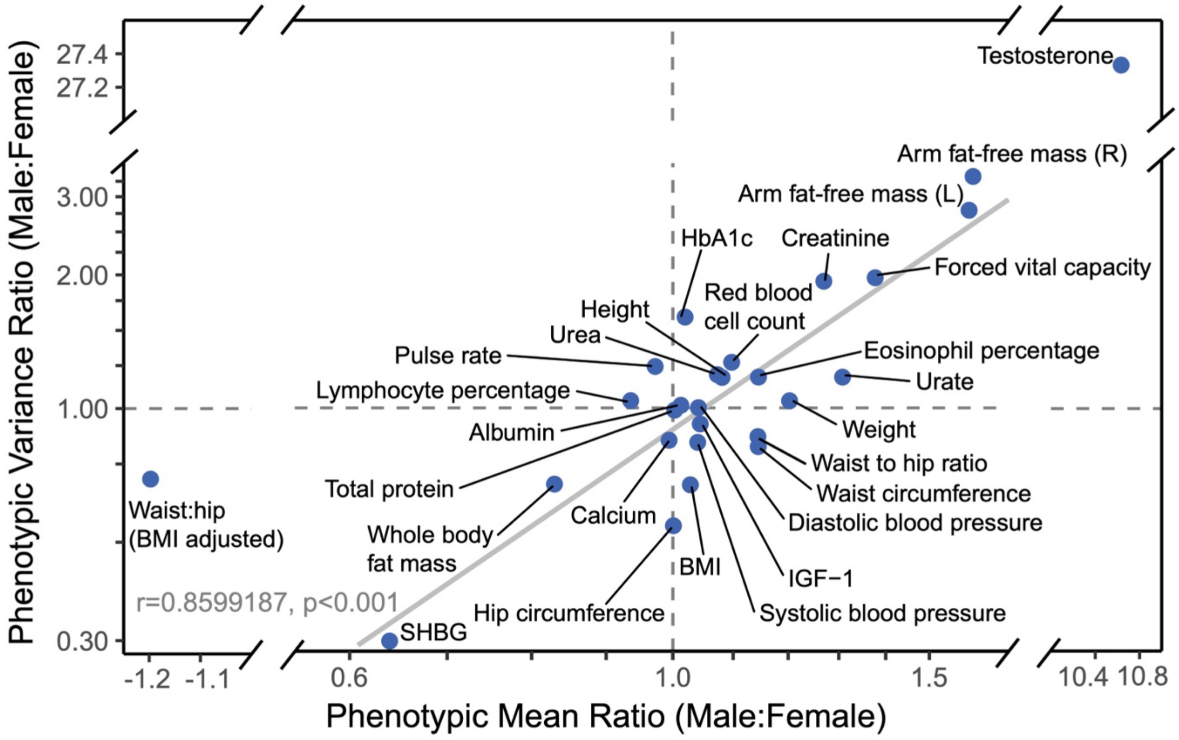

Figure S9: Process of generating posterior estimates from *mash*, related to Figure 3

Posterior mean and local false sign rate (lfsr) estimates are generated using *mash* from sex-specific summary statistics. We fit the model on the average of 100 mixture weights. The process is described in the **Text S7** section.

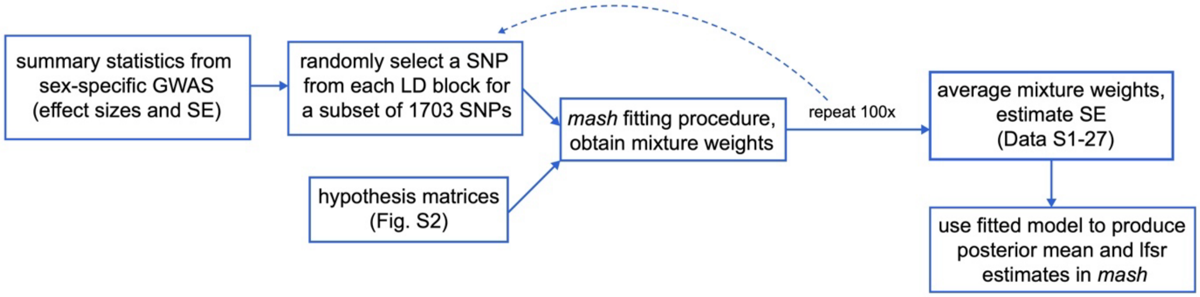

Figure S10: Process of comparing models for complex phenotype prediction, related to Figure 3

The process is described in **Text S8**. **(A)** The sample test set is obtained by sampling 25k males and 25k females. We then create the training set, by removing the test set from the original list of sample IDs. **(B)** After obtaining summary statistics from GWAS, we perform clumping to adjust for LD. Covariance-aware effect estimates are acquired by inputting the sex-specific summary statistics through *mash* to generate posterior mean and lfsr estimates **Fig. S9**. lfsr estimates are converted into pseudo p-values by matching to original p-values after ordering both from smallest to largest. We also conduct PGS analysis over four p-value thresholds on each of the five models, thus obtaining 20 PGS. **(C)** Prediction accuracy is measured based on R^2, from the linear regression of phenotype value on covariates and the polygenic scores. The incremental R^2 is also estimated by subtracting R^2 from the null model R^2, based on the regression of the phenotype value on just the covariates.

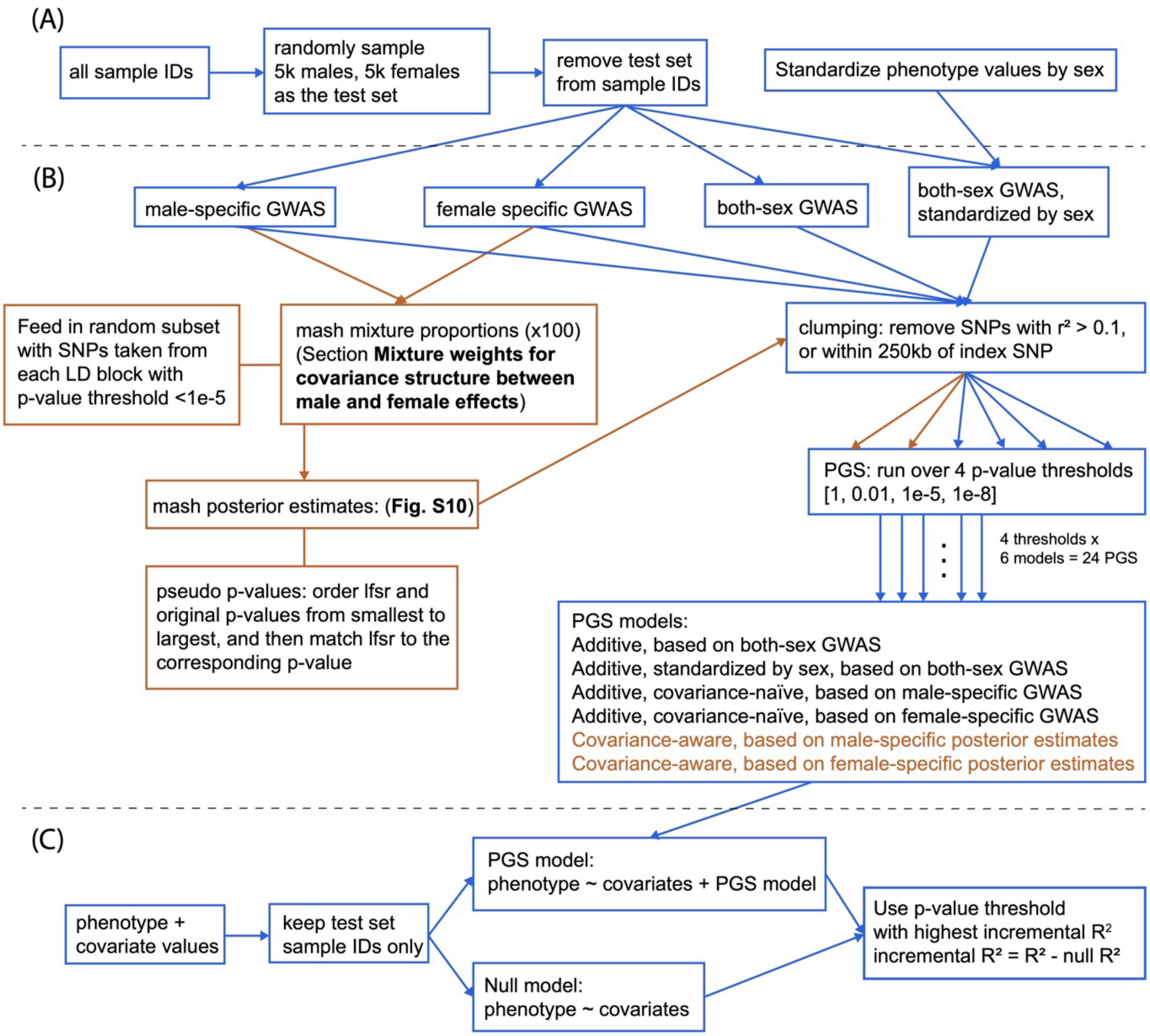

(A)
all sample IDs
randomly sample 5k males, 5k females as the test set
remove test set from sample IDs
Standardize phenotype values by sex
(B)
male-specific GWAS
female specific GWAS
both-sex GWAS
both-sex GWAS, standardized by sex
Feed in random subset with SNPs taken from each LD block with p-value threshold <1e-5
mash mixture proportions (x100) (Section **Mixture weights for covariance structure between male and female effects**)
mash posterior estimates: (**Fig. S10**)
pseudo p-values: order lfsr and original p-values from smallest to largest, and then match lfsr to the corresponding p-value
clumping: remove SNPs with r² > 0.1, or within 250kb of index SNP
PGS: run over 4 p-value thresholds [1, 0.01, 1e-5, 1e-8]
4 thresholds x 6 models = 24 PGS
PGS models:
Additive, based on both-sex GWAS
Additive, standardized by sex, based on both-sex GWAS
Additive, covariance-naïve, based on male-specific GWAS
Additive, covariance-naïve, based on female-specific GWAS
Covariance-aware, based on male-specific posterior estimates
Covariance-aware, based on female-specific posterior estimates
(C)
phenotype + covariate values
keep test set sample IDs only
PGS model: phenotype ~ covariates + PGS model
Null model: phenotype ~ covariates
Use p-value threshold with highest incremental R²
incremental R² = R² - null R²

Figure S11: Evaluating evidence for systematic amplification, related to Figure 2

We regressed the male (green) and female (orange) trait values to polygenic scores estimated in an independent sample of males (top) and females (bottom) separately. Each line corresponds to a separate regression of trait values in one sex to polygenic scores estimated in one sex. Each point represents the mean value in one decile of the polygenic score. The fitted line and associated R^2 are estimated from the raw, non-binned data.

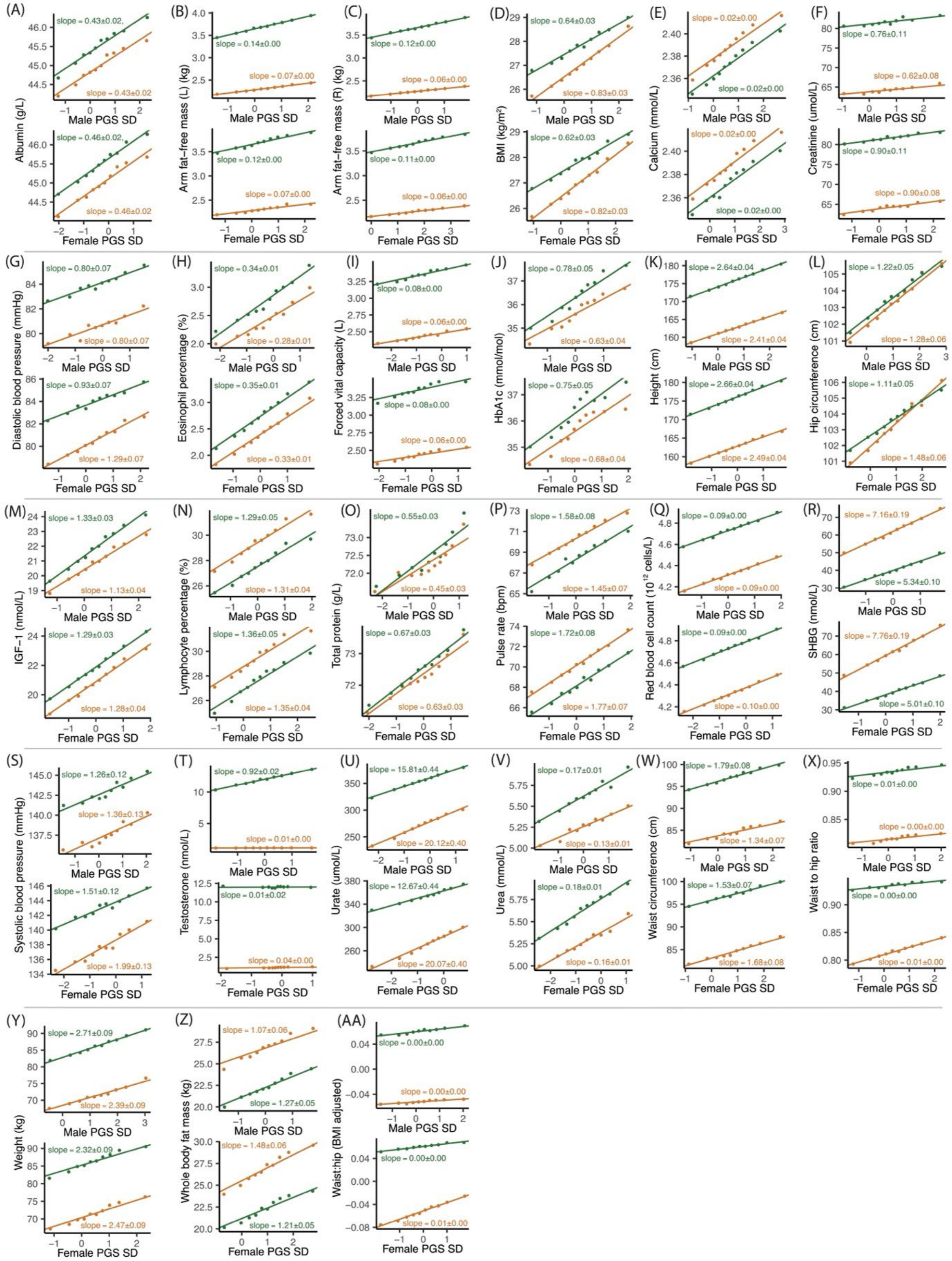

(A)
Albumin (g/L)
slope = 0.43±0.02,
slope = 0.43±0.02
slope = 0.46±0.02
slope = 0.46±0.02
(B)
Arm fat–free mass (L) (kg)
slope = 0.14±0.00
slope = 0.07±0.00
slope = 0.12±0.00
slope = 0.07±0.00
(C)
Arm fat–free mass (R) (kg)
slope = 0.12±0.00
slope = 0.06±0.00
slope = 0.11±0.00
slope = 0.06±0.00
(D)
BMI (kg/m²)
slope = 0.64±0.03
slope = 0.83±0.03
slope = 0.62±0.03
slope = 0.82±0.03
(E)
Calcium (mmol/L)
slope = 0.02±0.00
slope = 0.02±0.00
slope = 0.02±0.00
slope = 0.02±0.00
(F)
Creatinine (umol/L)
slope = 0.76±0.11
slope = 0.62±0.08
slope = 0.90±0.11
slope = 0.90±0.08
(G)
Diastolic blood pressure (mmHg)
slope = 0.80±0.07
slope = 0.80±0.07
slope = 0.93±0.07
slope = 1.29±0.07
(H)
Eosinophil percentage (%)
slope = 0.34±0.01
slope = 0.28±0.01
slope = 0.35±0.01
slope = 0.33±0.01
(I)
Forced vital capacity (L)
slope = 0.08±0.00
slope = 0.06±0.00
slope = 0.08±0.00
slope = 0.06±0.00
(J)
HbA1c (mmol/mol)
slope = 0.78±0.05
slope = 0.63±0.04
slope = 0.75±0.05
slope = 0.68±0.04
(K)
Height (cm)
slope = 2.64±0.04
slope = 2.41±0.04
slope = 2.66±0.04
slope = 2.49±0.04
(L)
Hip circumference (cm)
slope = 1.22±0.05
slope = 1.28±0.06
slope = 1.11±0.05
slope = 1.48±0.06
(M)
IGF−1 (nmol/L)
slope = 1.33±0.03
slope = 1.13±0.04
slope = 1.29±0.03
slope = 1.28±0.04
(N)
Lymphocyte percentage (%)
slope = 1.29±0.05
slope = 1.31±0.04
slope = 1.36±0.05
slope = 1.35±0.04
(O)
Total protein (g/L)
slope = 0.55±0.03
slope = 0.45±0.03
slope = 0.67±0.03
slope = 0.63±0.03
(P)
Pulse rate (bpm)
slope = 1.58±0.08
slope = 1.45±0.07
slope = 1.72±0.08
slope = 1.77±0.07
(Q)
Red blood cell count (10¹² cells/L)
slope = 0.09±0.00
slope = 0.09±0.00
slope = 0.09±0.00
slope = 0.10±0.00
(R)
SHBG (nmol/L)
slope = 7.16±0.19
slope = 5.34±0.10
slope = 7.76±0.19
slope = 5.01±0.10
(S)
Systolic blood pressure (mmHg)
slope = 1.26±0.12
slope = 1.36±0.13
slope = 1.51±0.12
slope = 1.99±0.13
(T)
Testosterone (nmol/L)
slope = 0.92±0.02
slope = 0.01±0.00
slope = 0.01±0.02
slope = 0.04±0.00
(U)
Urate (umol/L)
slope = 15.81±0.44
slope = 20.12±0.40
slope = 12.67±0.44
slope = 20.07±0.40
(V)
Urea (mmol/L)
slope = 0.17±0.01
slope = 0.13±0.01
slope = 0.18±0.01
slope = 0.16±0.01
(W)
Waist circumference (cm)
slope = 1.79±0.08
slope = 1.34±0.07
slope = 1.53±0.07
slope = 1.68±0.08
(X)
Waist to hip ratio
slope = 0.01±0.00
slope = 0.00±0.00
slope = 0.00±0.00
slope = 0.01±0.00
(Y)
Weight (kg)
slope = 2.71±0.09
slope = 2.39±0.09
slope = 2.32±0.09
slope = 2.47±0.09
(Z)
Whole body fat mass (kg)
slope = 1.07±0.06
slope = 1.27±0.05
slope = 1.48±0.06
slope = 1.21±0.05
(AA)
Waist:hip (BMI adjusted)
slope = 0.00±0.00
slope = 0.00±0.00
slope = 0.00±0.00
slope = 0.01±0.00
Male PGS SD
Female PGS SD

Figure S12: The predictive utility of G-by-Sex aware polygenic scores, related to Figure 3

We compared the incremental squared correlation (incremental R^2) of the phenotypic values and a predictor for four predictors. Incremental R^2 was obtained by taking the difference in R^2 between a prediction with covariates and the polygenic score and a prediction with only covariates. For each of the three models, we chose the highest incremental R^2 across four p-value thresholds (**Text S8**). The additive (purple), additive standardized by sex (yellow), sex-specific covariance-naïve (blue), and sex-specific covariance aware (red) models were calculated from effect estimates generated in GWAS, with the latter two in sex-stratified samples. The sex-specific, covariance aware model (red) refers to a polygenic score constructed using *mash* posterior effect estimates, which considers the covariance in genetic effects between the sexes. Sex-specific models contained a median sample size of around 123K males and 147K females, whereas the additive model retained the full training set of around 270K individuals. Incremental R^2 values and ±3 SE-wide error bars were computed using 20-fold cross-validation.

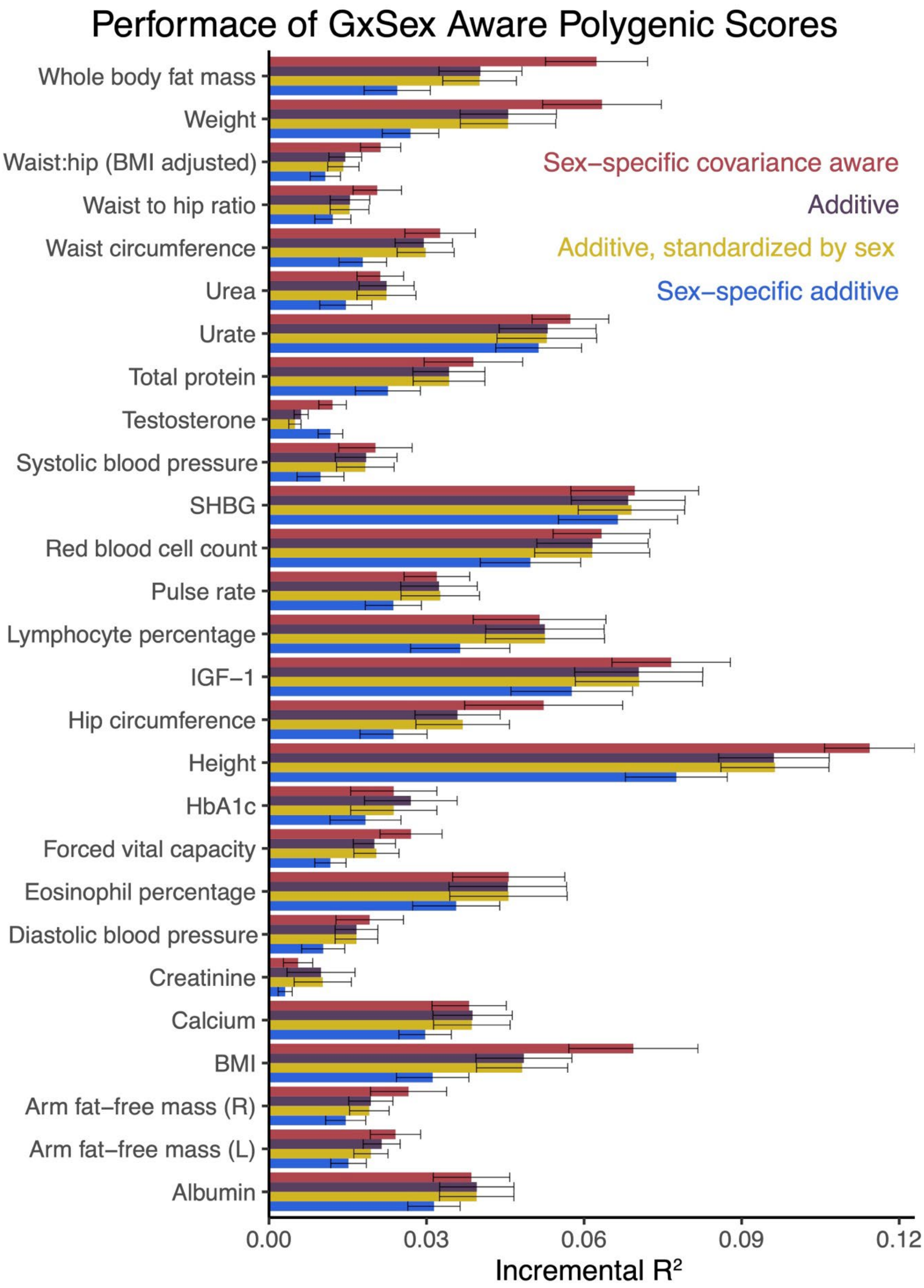

Performace of GxSex Aware Polygenic Scores
Sex–specific covariance aware
Additive
Additive, standardized by sex
Sex–specific additive
Whole body fat mass
Weight
Waist:hip (BMI adjusted)
Waist to hip ratio
Waist circumference
Urea
Urate
Total protein
Testosterone
Systolic blood pressure
SHBG
Red blood cell count
Pulse rate
Lymphocyte percentage
IGF–1
Hip circumference
Height
HbA1c
Forced vital capacity
Eosinophil percentage
Diastolic blood pressure
Creatinine
Calcium
BMI
Arm fat–free mass (R)
Arm fat–free mass (L)
Albumin
0.00
0.03
0.06
0.09
0.12
Incremental R²

Figure S13: Genetic effect and testosterone levels – using additive model PGS, related to Figure 5

The relationship between genetic effect and testosterone level bins, separated by sex, is depicted for all traits. Genetic effect, with ±1 SE error bars, is estimated from the slope of the regression of phenotypic values and polygenic scores from the additive model, multiplied by the polygenic score standard deviation (**Fig. S12**). The hollow data point represents a testosterone bin with overlapping range between males and females.

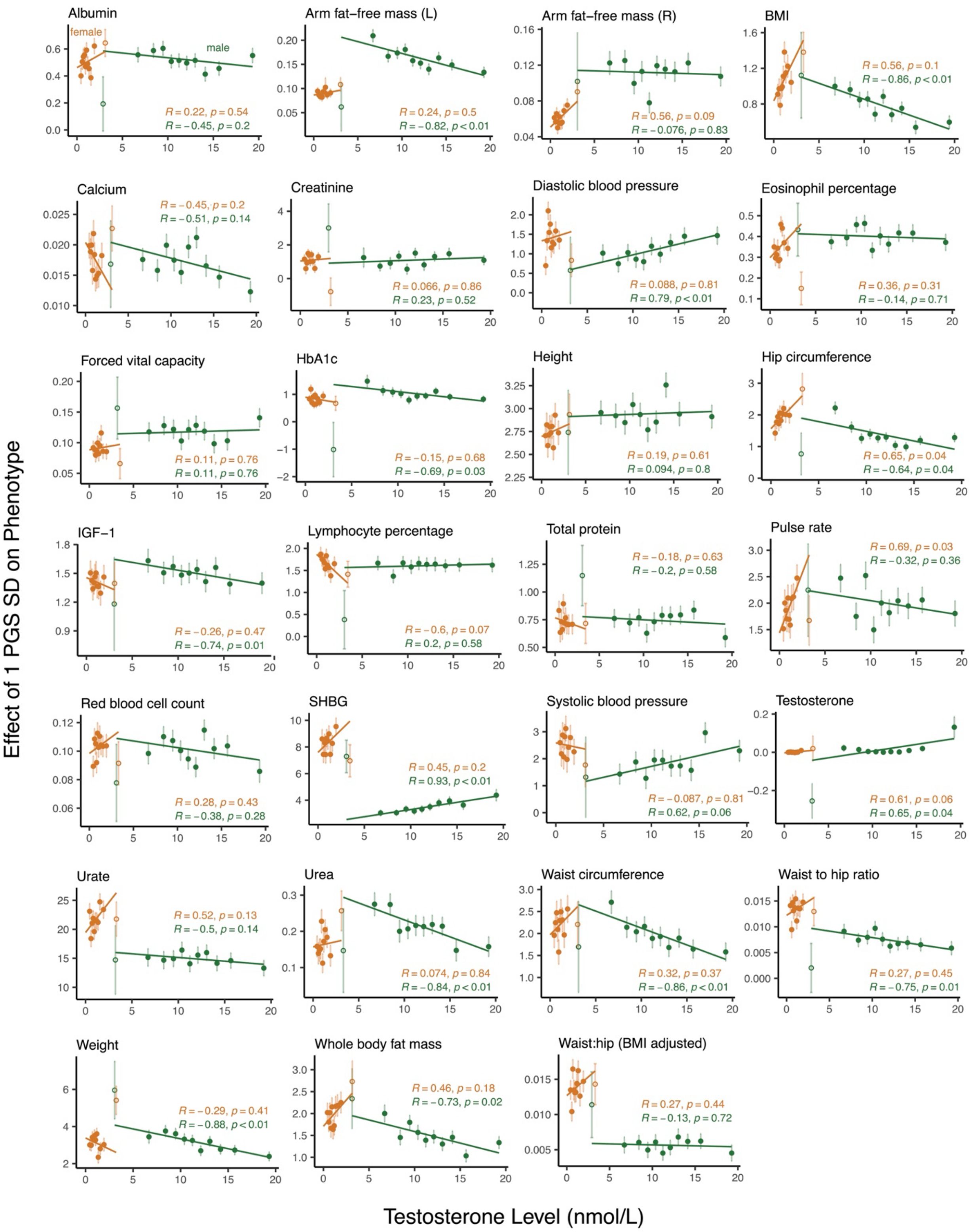
Effect of 1 PGS SD on Phenotype
Albumin
female
male
R = 0.22, p = 0.54
R = −0.45, p = 0.2
Arm fat−free mass (L)
R = 0.24, p = 0.5
R = −0.82, p < 0.01
Arm fat−free mass (R)
R = 0.56, p = 0.09
R = −0.076, p = 0.83
BMI
R = 0.56, p = 0.1
R = −0.86, p < 0.01
Calcium
R = −0.45, p = 0.2
R = −0.51, p = 0.14
Creatinine
R = 0.066, p = 0.86
R = 0.23, p = 0.52
Diastolic blood pressure
R = 0.088, p = 0.81
R = 0.79, p < 0.01
Eosinophil percentage
R = 0.36, p = 0.31
R = −0.14, p = 0.71
Forced vital capacity
R = 0.11, p = 0.76
R = 0.11, p = 0.76
HbA1c
R = −0.15, p = 0.68
R = −0.69, p = 0.03
Height
R = 0.19, p = 0.61
R = 0.094, p = 0.8
Hip circumference
R = 0.65, p = 0.04
R = −0.64, p = 0.04
IGF−1
R = −0.26, p = 0.47
R = −0.74, p = 0.01
Lymphocyte percentage
R = −0.6, p = 0.07
R = 0.2, p = 0.58
Total protein
R = −0.18, p = 0.63
R = −0.2, p = 0.58
Pulse rate
R = 0.69, p = 0.03
R = −0.32, p = 0.36
Red blood cell count
R = 0.28, p = 0.43
R = −0.38, p = 0.28
SHBG
R = 0.45, p = 0.2
R = 0.93, p < 0.01
Systolic blood pressure
R = −0.087, p = 0.81
R = 0.62, p = 0.06
Testosterone
R = 0.61, p = 0.06
R = 0.65, p = 0.04
Urate
R = 0.52, p = 0.13
R = −0.5, p = 0.14
Urea
R = 0.074, p = 0.84
R = −0.84, p < 0.01
Waist circumference
R = 0.32, p = 0.37
R = −0.86, p < 0.01
Waist to hip ratio
R = 0.27, p = 0.45
R = −0.75, p = 0.01
Weight
R = −0.29, p = 0.41
R = −0.88, p < 0.01
Whole body fat mass
R = 0.46, p = 0.18
R = −0.73, p = 0.02
Waist:hip (BMI adjusted)
R = 0.27, p = 0.44
R = −0.13, p = 0.72
Testosterone Level (nmol/L)

Figure S14: Genetic effect and testosterone levels – using sex-specific model PGS, related to Figure 5

The same type of figures as **Fig. S13** are displayed with the exception of utilizing PGS from the sex-specific, covariance-naïve model for estimation of genetic effect.

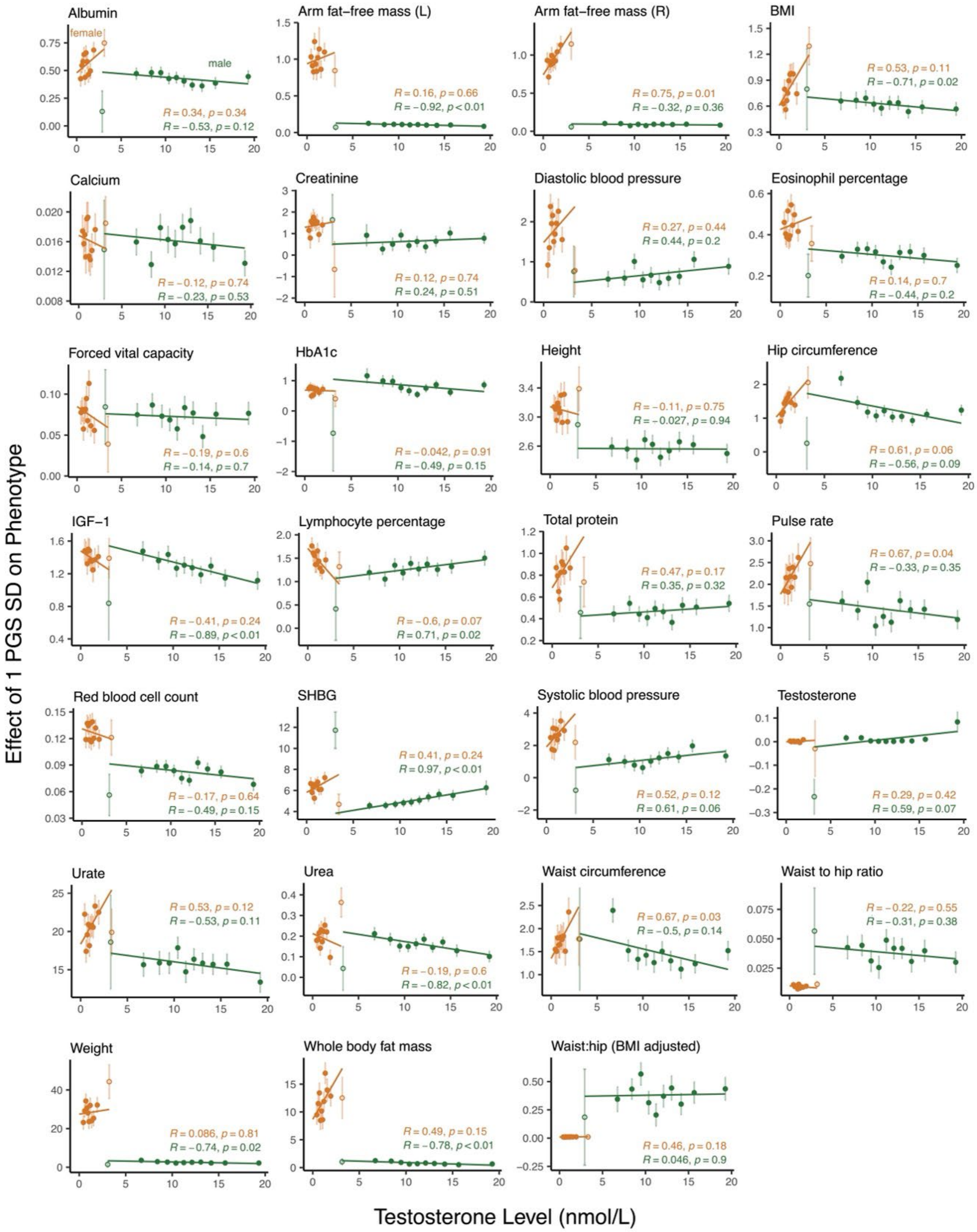

Figure S15: Genetic effect and PGS for testosterone, related to Figure 5

We used mendelian randomization to assess the causal relationship between genetic effect and testosterone levels, similar to **Fig. S13**. **(A)** The plots illustrate the relationship between amplification of total genetic effect of each trait and PGS based on sex-specific summary statistics for testosterone. Genetic effect, with ±1 SE error bars, is estimated from the slope of the regression of phenotype values on the PGS based on additive summary statistics in each PGS bin for testosterone, multiplied by the PGS standard deviation. The x-axis takes the mean PGS for testosterone from each bin. **(B)** The correlation for each sex (90% CI) and all traits between genetic effect and PGS for testosterone across the bins is shown, with traits in descending order by the difference of correlation estimates between the sexes.

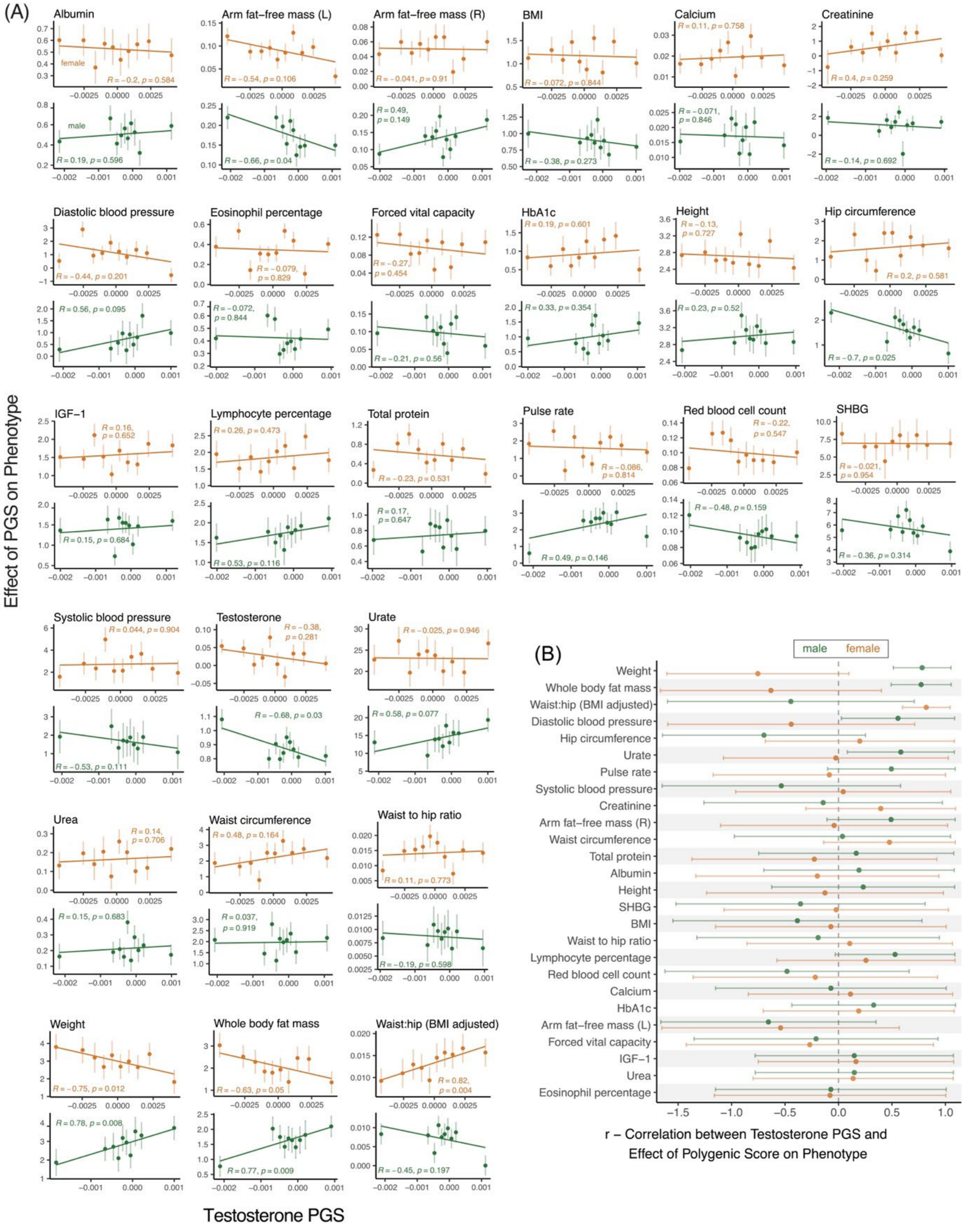
(A)
Albumin
Arm fat−free mass (L)
Arm fat−free mass (R)
BMI
Calcium
Creatinine
female
male
Diastolic blood pressure
Eosinophil percentage
Forced vital capacity
HbA1c
Height
Hip circumference
IGF−1
Lymphocyte percentage
Total protein
Pulse rate
Red blood cell count
SHBG
Systolic blood pressure
Testosterone
Urate
Urea
Waist circumference
Waist to hip ratio
Weight
Whole body fat mass
Waist:hip (BMI adjusted)
Effect of PGS on Phenotype
Testosterone PGS
(B)
male
female
Weight
Whole body fat mass
Waist:hip (BMI adjusted)
Diastolic blood pressure
Hip circumference
Urate
Pulse rate
Systolic blood pressure
Creatinine
Arm fat−free mass (R)
Waist circumference
Total protein
Albumin
Height
SHBG
BMI
Waist to hip ratio
Lymphocyte percentage
Red blood cell count
Calcium
HbA1c
Arm fat−free mass (L)
Forced vital capacity
IGF−1
Urea
Eosinophil percentage
−1.5
−1.0
−0.5
0.0
0.5
1.0
r − Correlation between Testosterone PGS and
Effect of Polygenic Score on Phenotype

Figure S16: Genetic effect residualized for age and testosterone levels, related to Figure 5

In each bin, we examined the relationship between the polygenic effect residualized for mean age and the mean testosterone level. This plot shows the correlation (90% CI) between the two across ten bins.

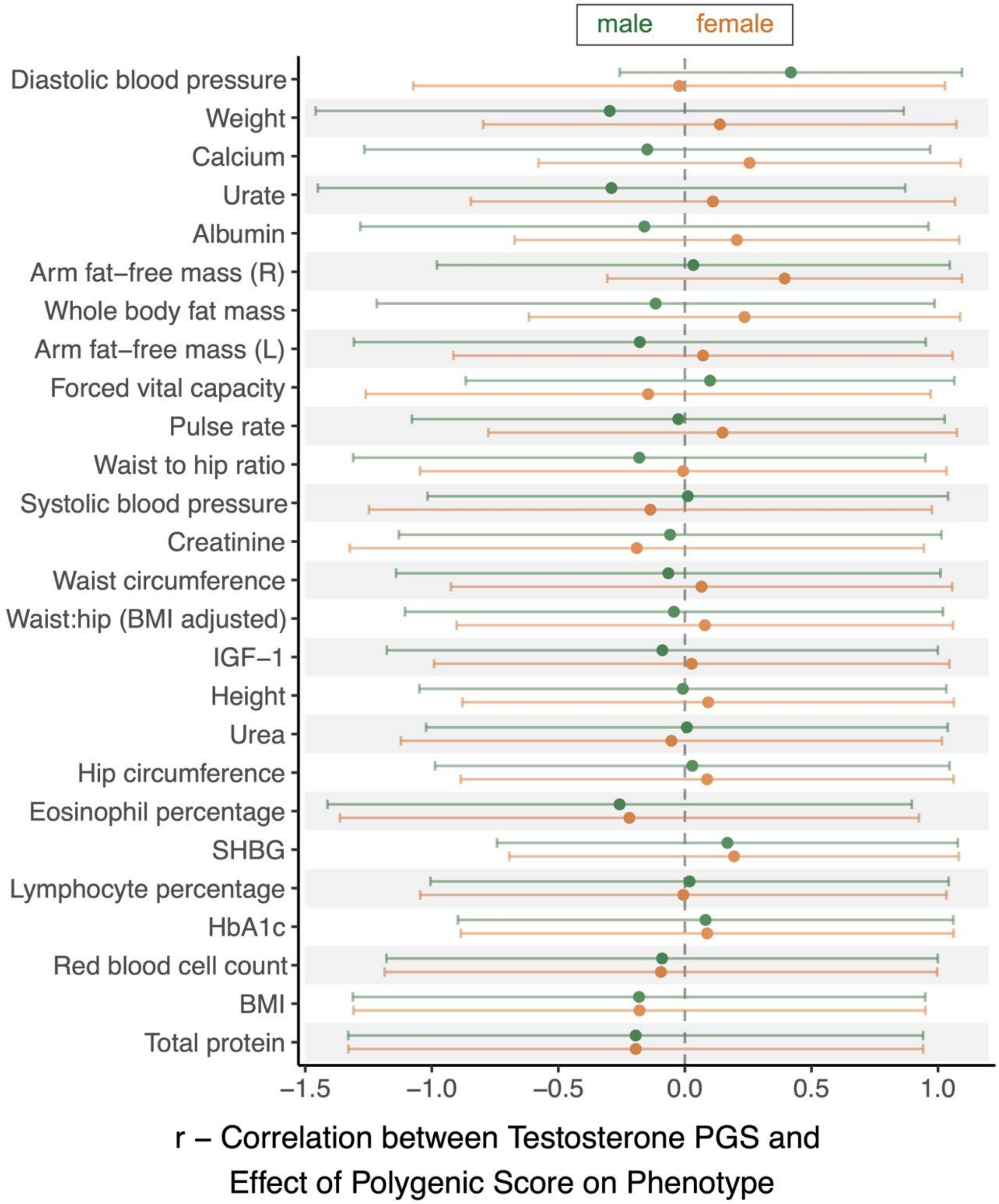

Figure S17: Example of shared polygenic and environmental amplification in muscle, related to Figure 6

An example of equal amplification of genetic and environmental effect (**"Are polygenic and environmental effects jointly amplified?"** in main text**)** is depicted in muscle after resistance training. The example follows the model in **Fig. 6A**, in which the genetic effect (genetic regulation) and environmental effect (resistance exercise), both affect a core pathway (involving IGF-1) for muscle. The effect size of the core pathway is then amplified by a modulator such as testosterone, producing the sexual-dimorphism shown in muscle.

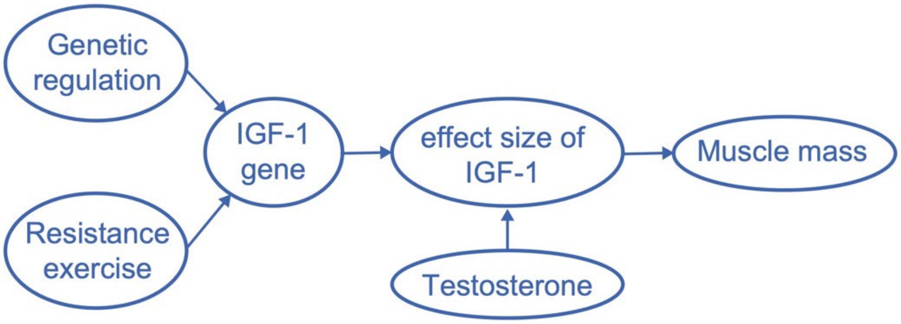

Figure S18: Competing models for sex differences in trait variance, related to Figure 6

We compare two different models for sex differences in trait variance, greater-male and estrus-mediated variability as discussed in Zajitschek et al.[21] and **Text S11**, with our model of pervasive amplification linking genetic and environmental effects ("**Are polygenic and environmental effects jointly amplified?"** in main text**). (A)** We illustrate the difference between the variability between ($V_{between}$) and the variability within (V_{within}) using individual distributions. We define the three models according to the relationship between male and female $V_{between}$ and $V_{within.}$ **(B)** The three models are superimposed as different colored lines on **Fig. 6B** to compare the distribution of traits across the models. Traits in blue, green, and yellow are consistent (within 90% CI) with the model of pervasive amplification, greater-male variability, and estrus-mediated variability alone, respectively. Traits in grey are consistent with more than one model. Traits in black are inconsistent with either model.

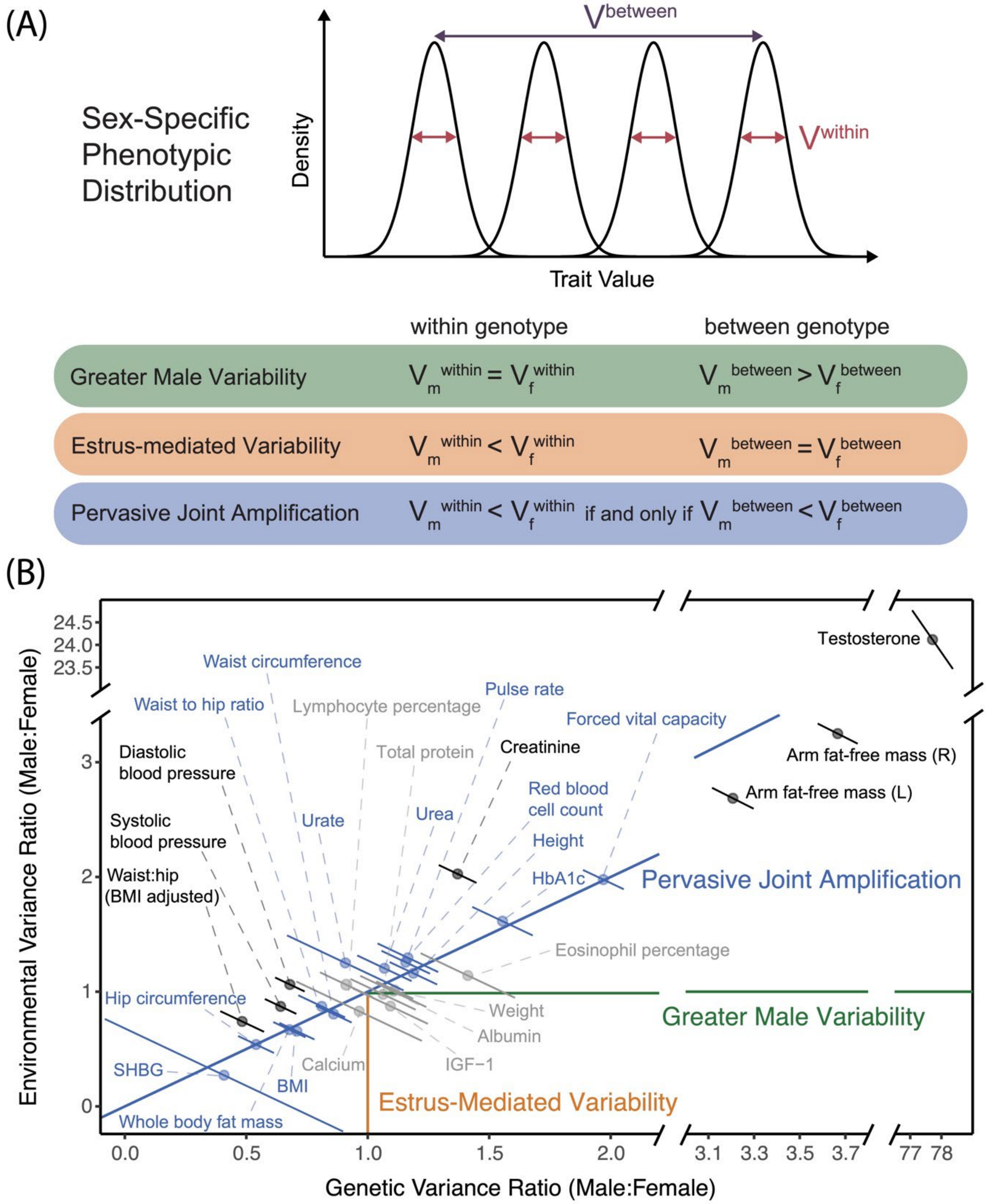

(A)
Sex-Specific Phenotypic Distribution
Density
Trait Value
Vbetween
Vwithin
within genotype
between genotype
Greater Male Variability
$V_m^{within} = V_f^{within}$
$V_m^{between} > V_f^{between}$
Estrus-mediated Variability
$V_m^{within} < V_f^{within}$
$V_m^{between} = V_f^{between}$
Pervasive Joint Amplification
$V_m^{within} < V_f^{within}$ if and only if $V_m^{between} < V_f^{between}$
(B)
Environmental Variance Ratio (Male:Female)
Genetic Variance Ratio (Male:Female)
Testosterone
Waist circumference
Pulse rate
Waist to hip ratio
Lymphocyte percentage
Forced vital capacity
Diastolic blood pressure
Total protein
Creatinine
Arm fat-free mass (R)
Arm fat-free mass (L)
Red blood cell count
Urate
Urea
Systolic blood pressure
Height
Waist:hip (BMI adjusted)
HbA1c
Pervasive Joint Amplification
Eosinophil percentage
Hip circumference
Weight
Greater Male Variability
Albumin
SHBG
Calcium
IGF−1
BMI
Estrus-Mediated Variability
Whole body fat mass

Figure S19: Z-scores for strength of sexually-antagonistic selection, related to Figure 7

Shown are Z-scores for the strength of sexually-antagonistic selection, *A* in **Eq 1**, computed for various gnomAD subsamples (categorized by ancestry group). Different panels show results obtained with different p-value thresholds on marginal significance of sex-specific association in the GWAS (in either sex), including 10^{-8} **(A)**, 10^{-5} **(B)**, and 10^{-3} **(C)**. Bars show 90% resampling confidence intervals.

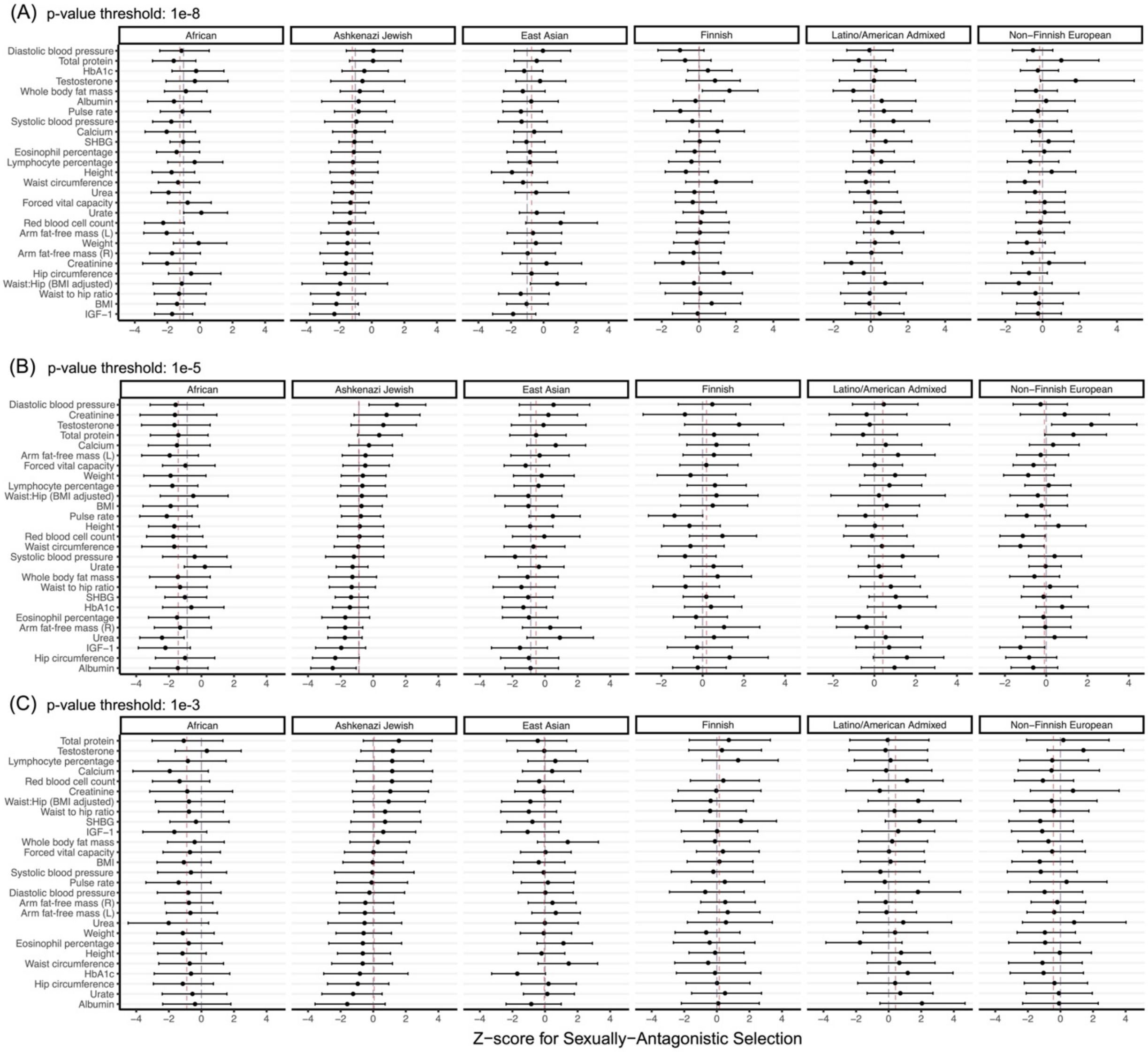

Figure S20: Z-scores using UK Biobank data, related to Figure 7

This figure parallels **Fig. 7** and **Fig. S19** but uses male-female allele frequency differentiation data from the UK BioBank. Here, the statistic L_{ST} replaces F_{ST}, as explained in the section "**Estimating the potential for sexually-antagonistic selection on standing variation (*A*)**" of the main text. The p-value cutoff for marginal sex-specific GWAS associations included in this analysis was set to 10^{-5}. Bars show 90% resampling confidence intervals.

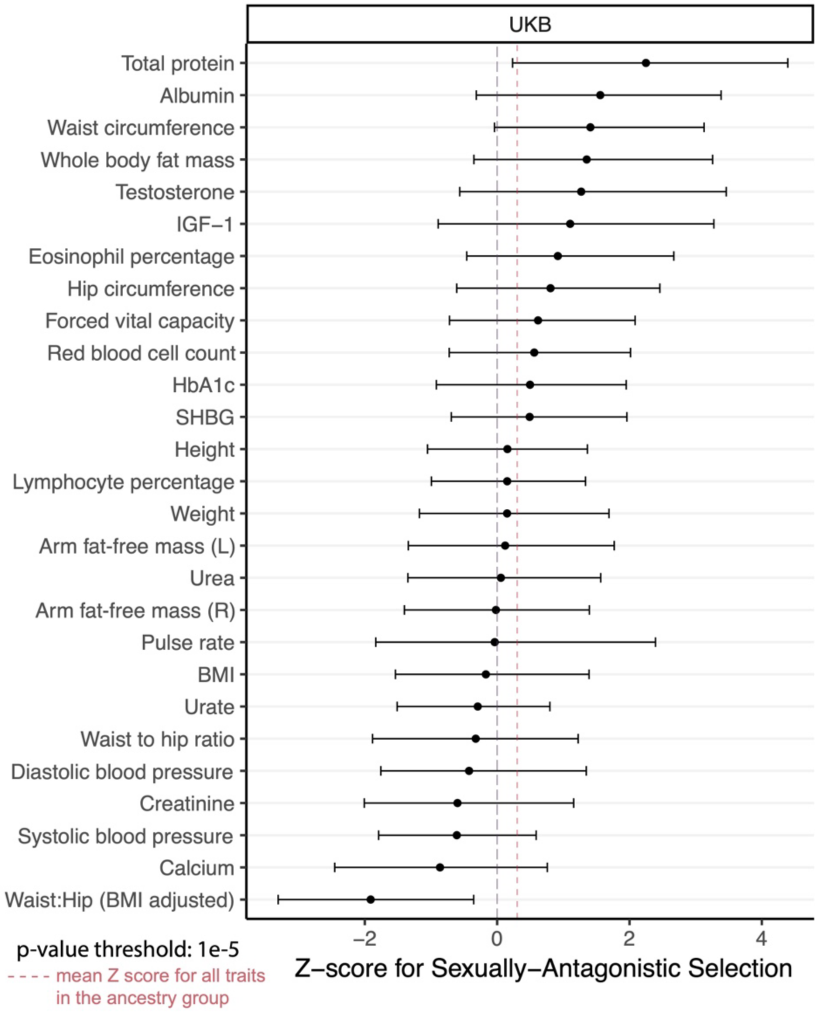

Supplementary Tables

Table S1: Filtering site with small sample sizes, related to Figure 7

For the sexually-antagonistic selection analysis, we removed all sites with less than 1,000 alleles in the sample in order to narrow our research on sites with sufficiently large sample sizes. There were 2,285,169 sites before filtering.

Ancestry	# Sites After Filtering
African/African American	2,282,394
Amish	0
Latino/American Admixed	2,282,394
Ashkenazi Jewish	2,247,203
East Asian	2,255,778
Finnish	2,249,604
Middle Eastern	0
Non-Finnish European	2,283,952
South Asian	2,167,009
Other	2,080,336

Table S2. Filtering monoallelic sites, related to Figure 7

For the sexually-antagonistic selection analysis, and for each ancestry, we removed sites which were monoallelic in one or both sexes.

Ancestry	# Sites after filtering
African/African American	924,946
Latino/American Admixed	519,496
Ashkenazi Jewish	161,271
East Asian	300,323
Finnish	207,162
Non-Finnish European	1,104,008
South Asian	330,518
Other	267,812

Table S3: Filtering sites that were not overlapping between the gnomAD dataset and the GWAS dataset, related to Figure 7

For the sexually-antagonistic selection analysis, any sites which did not occur in both the filtered GWAS data from UKB and the filtered gnomAD data were excluded.

Ancestry	# Sites before filtering by GWAS-gnomAD overlap	# Sites after filtering	# Sites removed
African/African American	924,946	886,467	38,479
Latino/American Admixed	519,496	506,760	12,736
Ashkenazi Jewish	161,271	160,358	913
East Asian	300,323	295,801	4,522
Finnish	207,162	204,914	2,248
Non-Finnish European	1,104,008	1,051,015	52,993
South Asian	330,518	325,444	5,074
Other	267,812	264,682	3,130

Table S4: Filtering by p-value threshold, related to Figure 7

For the sexually-antagonistic selection analysis, we used three different thresholds of GWAS p-values to further restrict which sites were used for analysis by focusing on sites with strong correlation with the trait of interest. Shown are the results of filtering for height, as an example. Results for other traits are highly similar.

Ancestry	p-value threshold	# Sites before this filtering step	# Sites after filtering
Ashkenazi Jewish	1e-3	160,358	2,749
Ashkenazi Jewish	1e-5	160,358	578
Ashkenazi Jewish	1e-8	160,358	298
Finnish	1e-3	204,914	3,975
Finnish	1e-5	204,914	852
Finnish	1e-8	204,914	373

Table S5: Identification of SNPs susceptible to mis-mapping to sex chromosomes—Kasimatis et al., related to Figure 7

We compare our results for excluding sites due to mis-mapping to the results from Kasimatis et al.[18], who removed from their analysis sites in regions with 90% identity or higher with a region in a sex chromosome (as we have, based on BLAT[25] matches), as well as sites overlapping probes of length 40bp or higher.

90% BLAT identity threshold	This study, susceptible to mis-mapping	This study, not identified as susceptible
Kasimatis et al., suceptible to mis-hybridization	15,565 (2.51%)	4,963 (0.80%)
Kasimatis et al., not identified as susceptible	469,864 (75.78%)	129,648 (20.91%)

Table S6: Characterizing GxSex based on independent analysis of individual sex-heterogenous SNPs, related to Figure 3

We simulated GWAS data study arising from the covariance structure inferred for five traits. We then followed the method described in **Text S6** for calling SNPs as sex-heterogenous SNPs and categorizing them.

Generative covariance structure	# sex-heterogenous SNPs	% sex-specific effects	% effects in the opposite direction	% effects in the same directions
Height	994.7 (13.72)	62.3% (0.6%)	37.7% (0.6%)	0.0% (0.0%)
BMI	1527.7 (50.74)	52.1% (2.7%)	26.8% (0.9%)	21.1% (0.9%)
Creatinine	1243.6 (7.67)	52.8% (0.4%)	30.7% (0.4%)	16.4% (0.4%)
IGF-1	1392.3 (10.82)	52.2% (0.5%)	29.1% (0.7%)	18.7% (0.4%)
Systolic blood pressure	1349.2 (15.68)	51.7% (0.3%)	28.4% (0.3%)	20.0% (0.4%)

Supplementary Data

Data S1-S27: Mixture weights on hypothesis covariance matrices, related to Figure 3

Figures similar to **Fig. S3B, C** for all traits. Mixture weights (±SE) estimated from a sample of SNP effects using *mash* are shown with the corresponding hypothesis covariance matrix represented by the axes. Covariance matrices span all hypothesis matrices shown in **Fig. S2**. The "Weight of No Effect Matrix" box represents the weight on a matrix signifying no effects in both sexes. The bottom plot for each traits represents a consolidated version of the top plot. Weights on the bottom plot are shown as percentages of non-null weights.

Data S1: Albumin

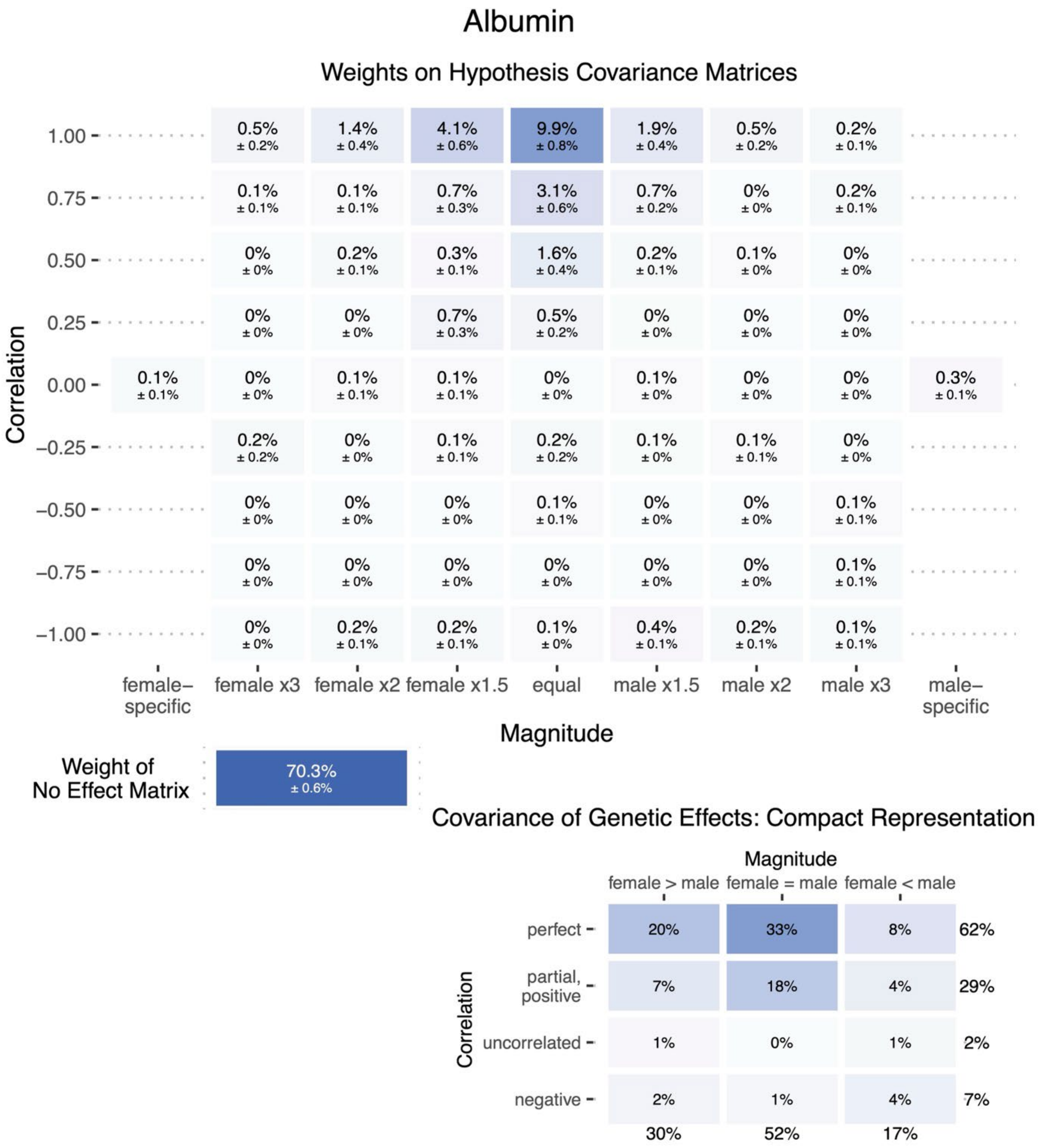

Data S2: Arm fat-free mass (L)

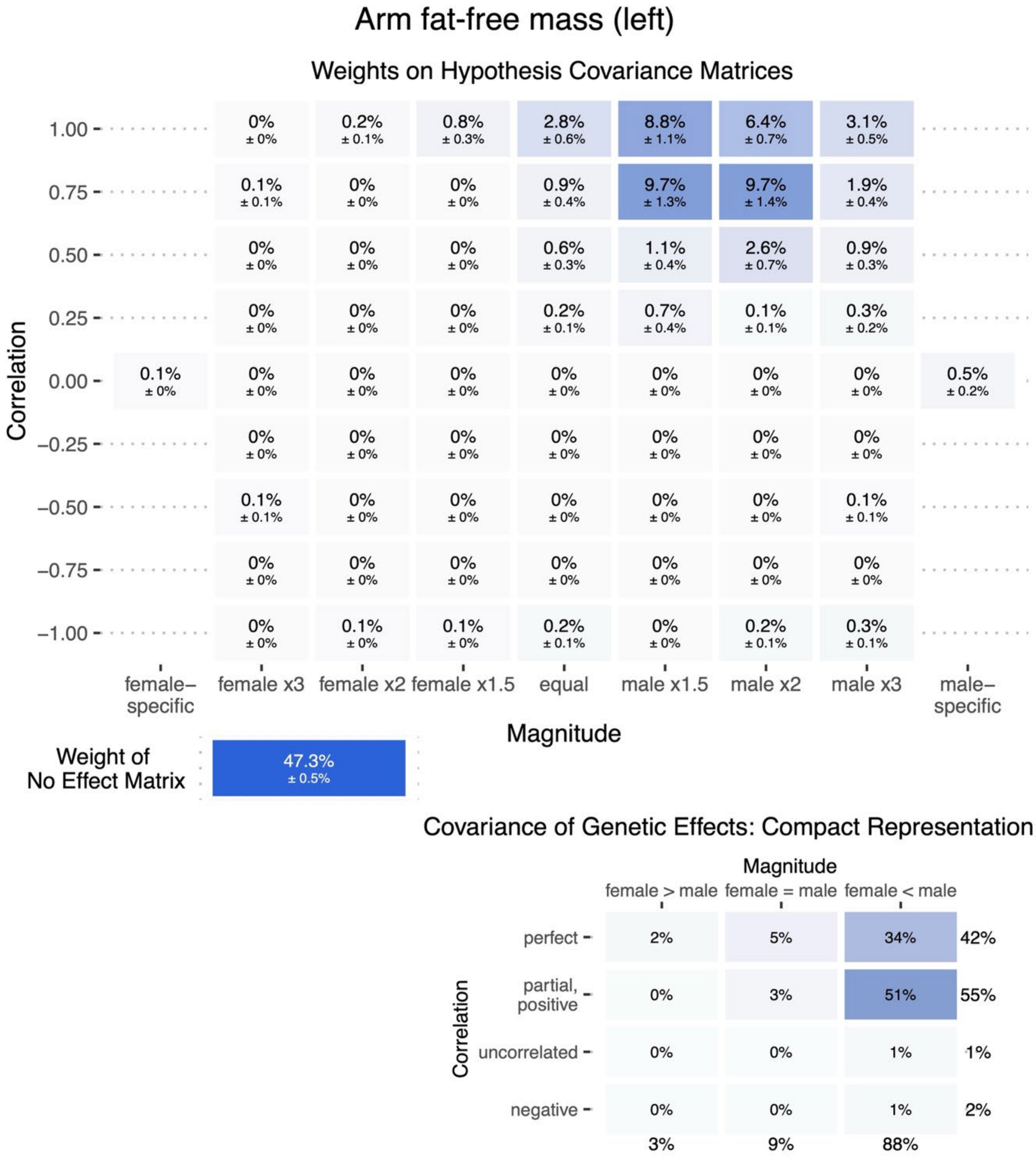

Data S3: Arm fat-free mass (R)

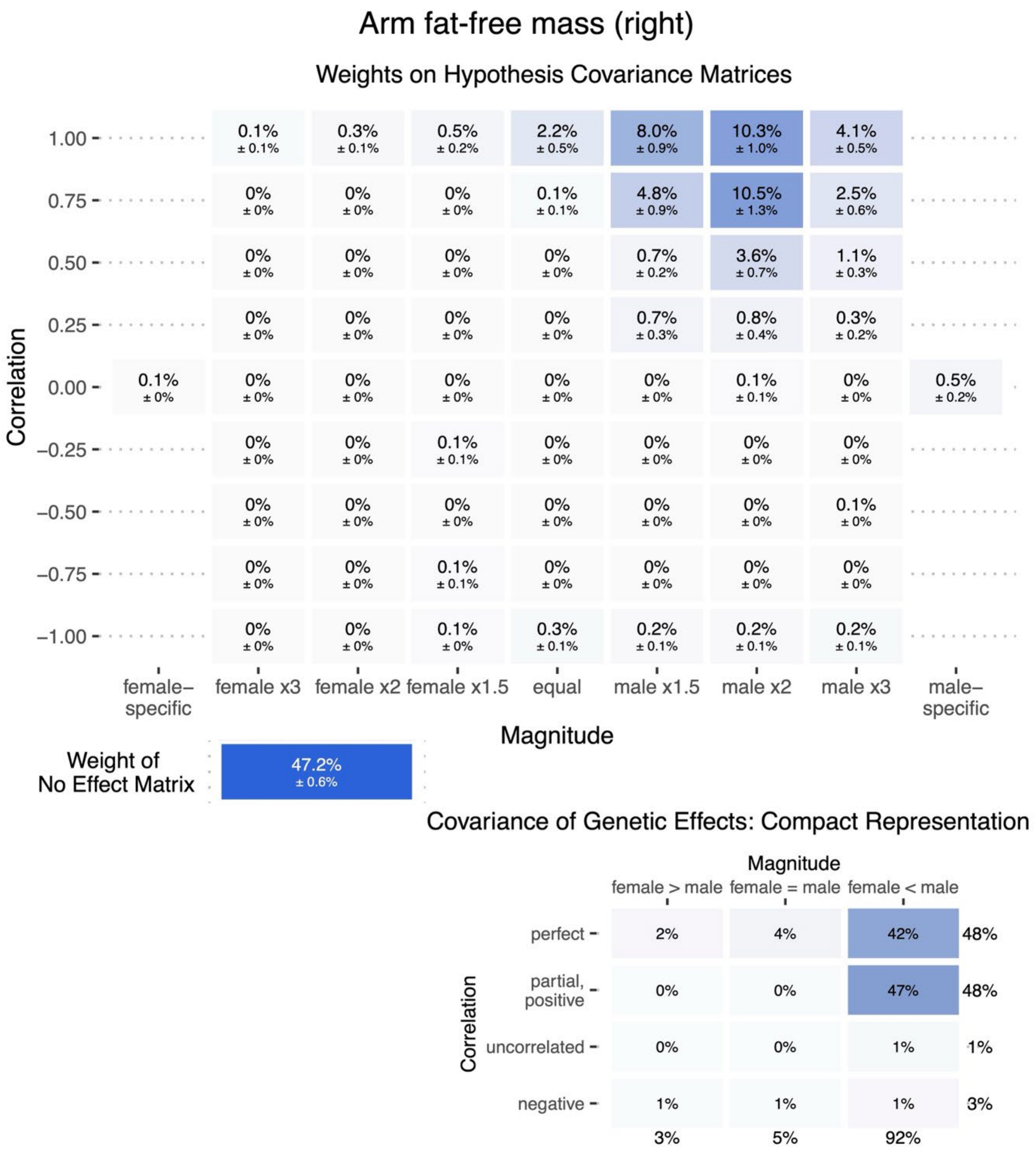

Data S4: BMI

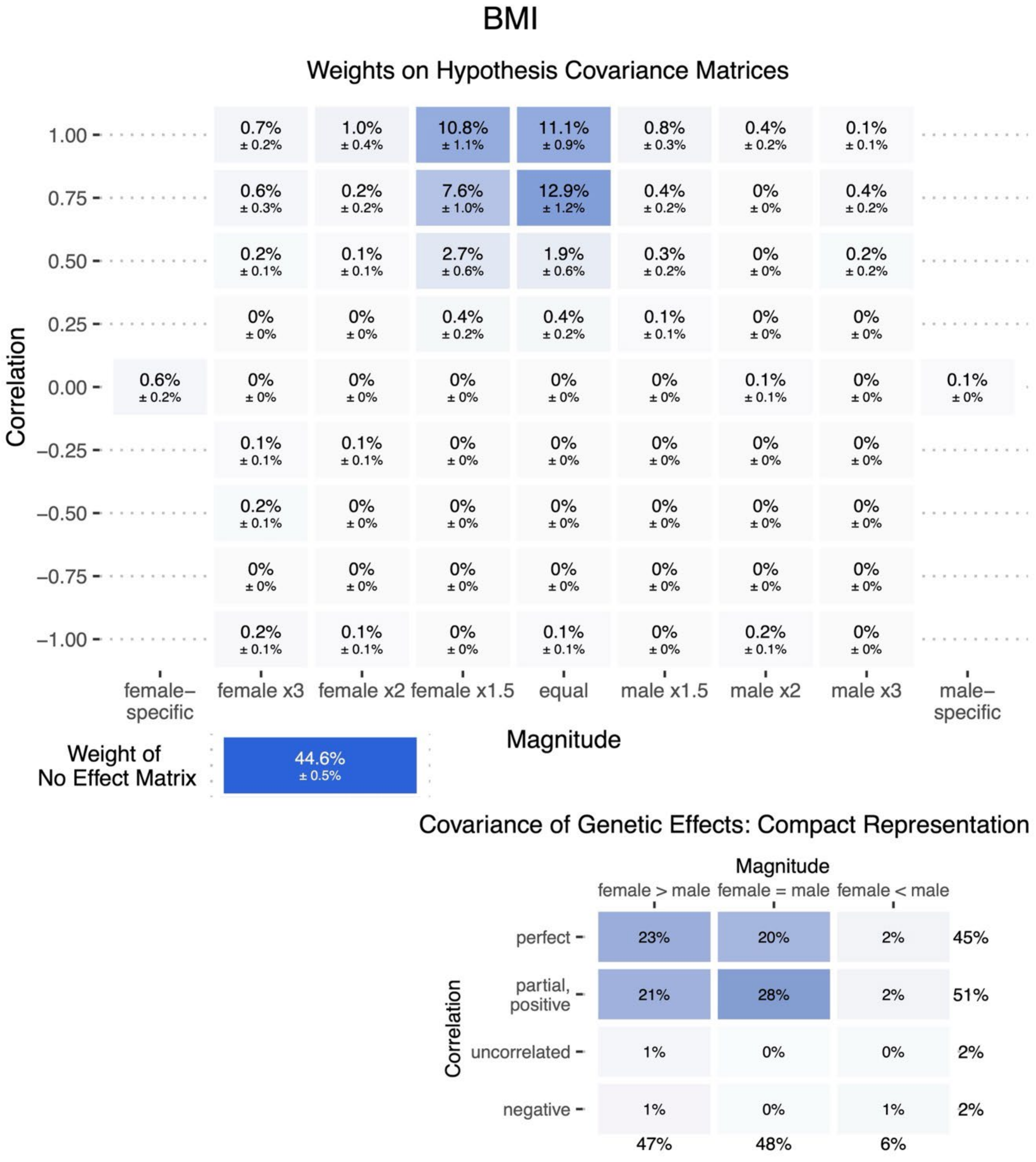

Correlation	female–specific	female x3	female x2	female x1.5	equal	male x1.5	male x2	male x3	male–specific
1.00		0.7% ± 0.2%	1.0% ± 0.4%	10.8% ± 1.1%	11.1% ± 0.9%	0.8% ± 0.3%	0.4% ± 0.2%	0.1% ± 0.1%	
0.75		0.6% ± 0.3%	0.2% ± 0.2%	7.6% ± 1.0%	12.9% ± 1.2%	0.4% ± 0.2%	0% ± 0%	0.4% ± 0.2%	
0.50		0.2% ± 0.1%	0.1% ± 0.1%	2.7% ± 0.6%	1.9% ± 0.6%	0.3% ± 0.2%	0% ± 0%	0.2% ± 0.2%	
0.25		0% ± 0%	0% ± 0%	0.4% ± 0.2%	0.4% ± 0.2%	0.1% ± 0.1%	0% ± 0%	0% ± 0%	
0.00	0.6% ± 0.2%	0% ± 0%	0% ± 0%	0% ± 0%	0% ± 0%	0% ± 0%	0.1% ± 0.1%	0% ± 0%	0.1% ± 0%
−0.25		0.1% ± 0.1%	0.1% ± 0.1%	0% ± 0%	0% ± 0%	0% ± 0%	0% ± 0%	0% ± 0%	
−0.50		0.2% ± 0.1%	0% ± 0%	0% ± 0%	0% ± 0%	0% ± 0%	0% ± 0%	0% ± 0%	
−0.75		0% ± 0%	0% ± 0%	0% ± 0%	0% ± 0%	0% ± 0%	0% ± 0%	0% ± 0%	
−1.00		0.2% ± 0.1%	0.1% ± 0.1%	0% ± 0%	0.1% ± 0.1%	0% ± 0%	0.2% ± 0.1%	0% ± 0%	

Correlation	female > male	female = male	female < male	
perfect	23%	20%	2%	45%
partial, positive	21%	28%	2%	51%
uncorrelated	1%	0%	0%	2%
negative	1%	0%	1%	2%
	47%	48%	6%	

Data S5: Calcium

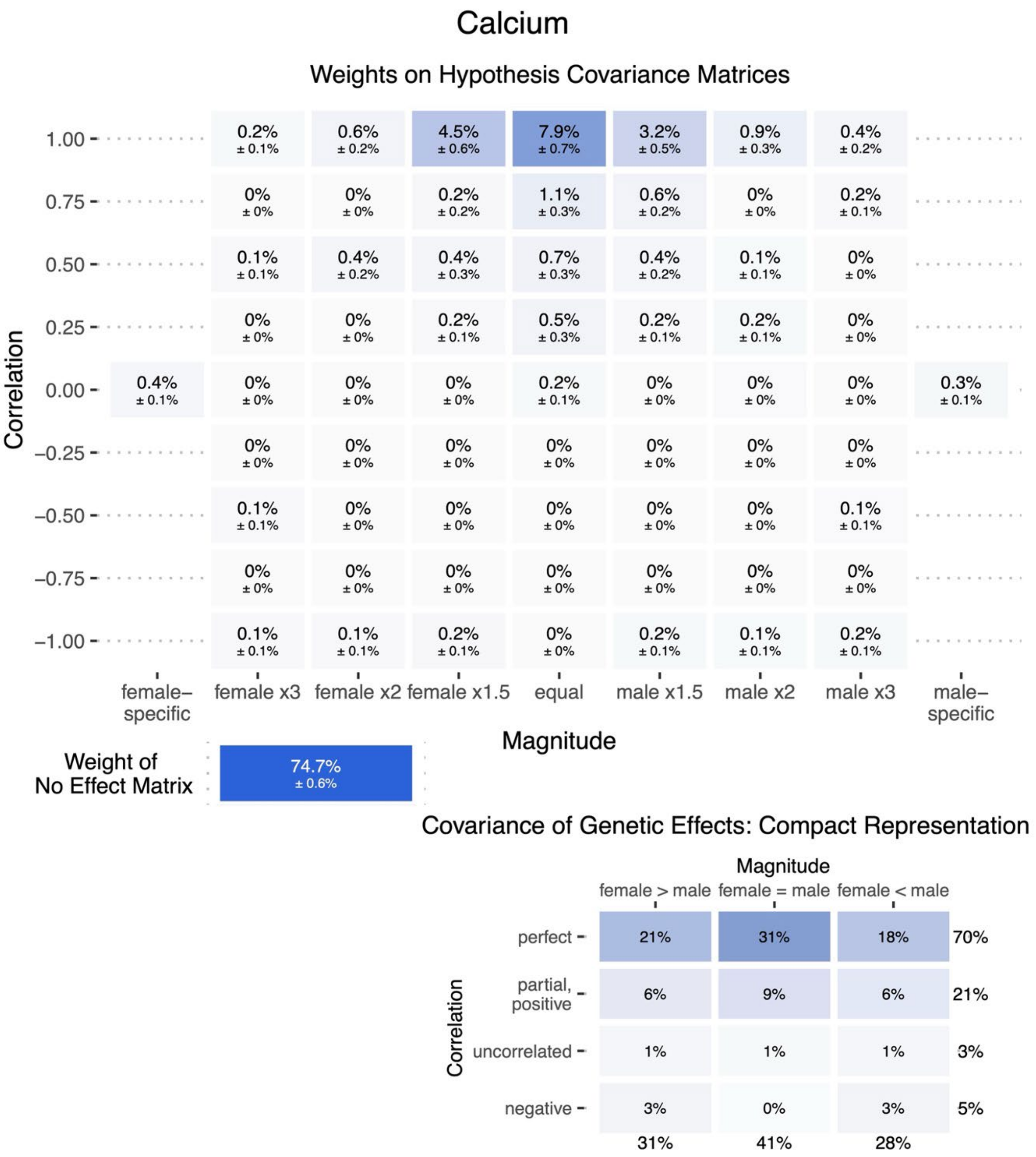

Data S6: Creatinine

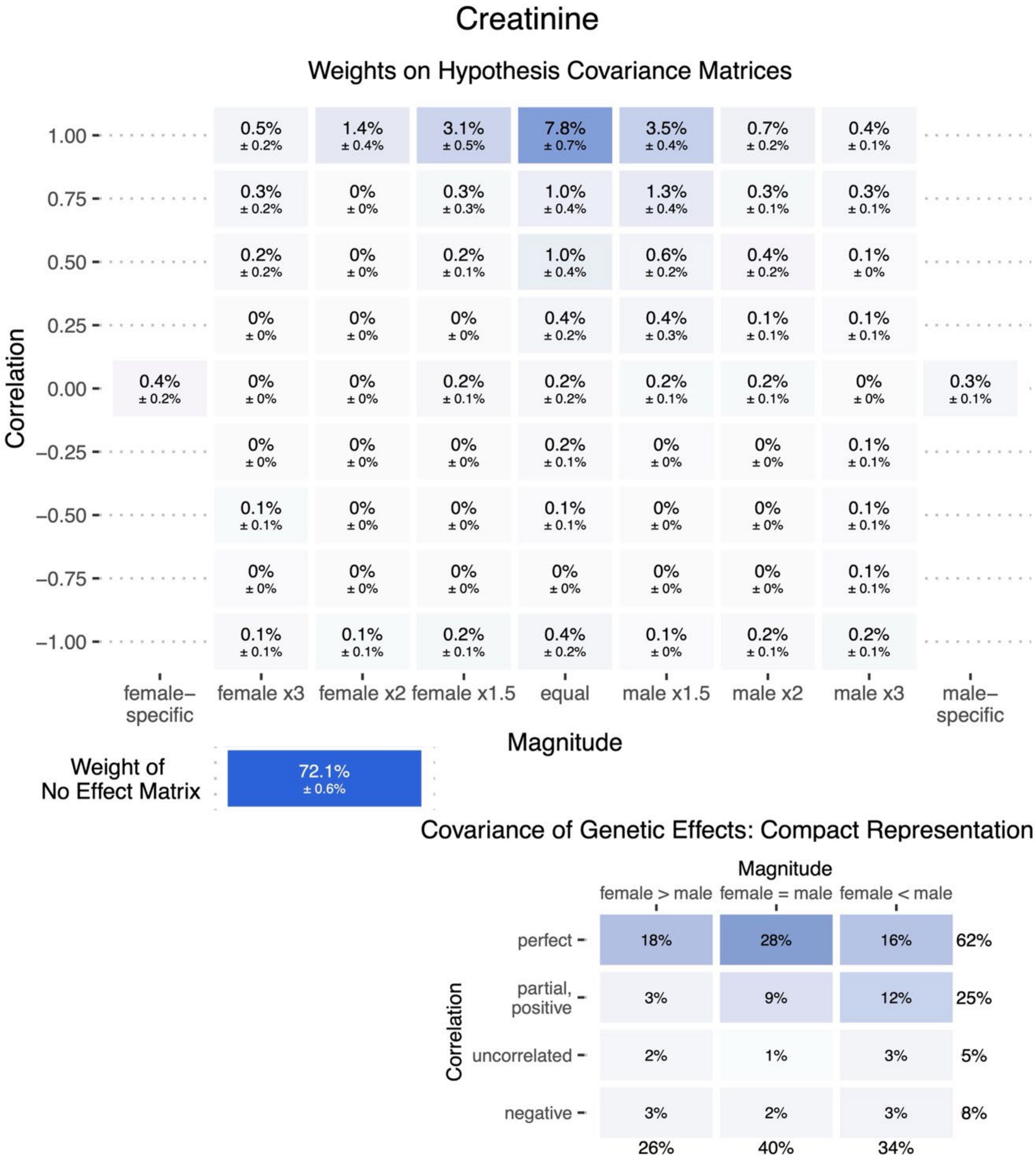

Data S7: Diastolic blood pressure

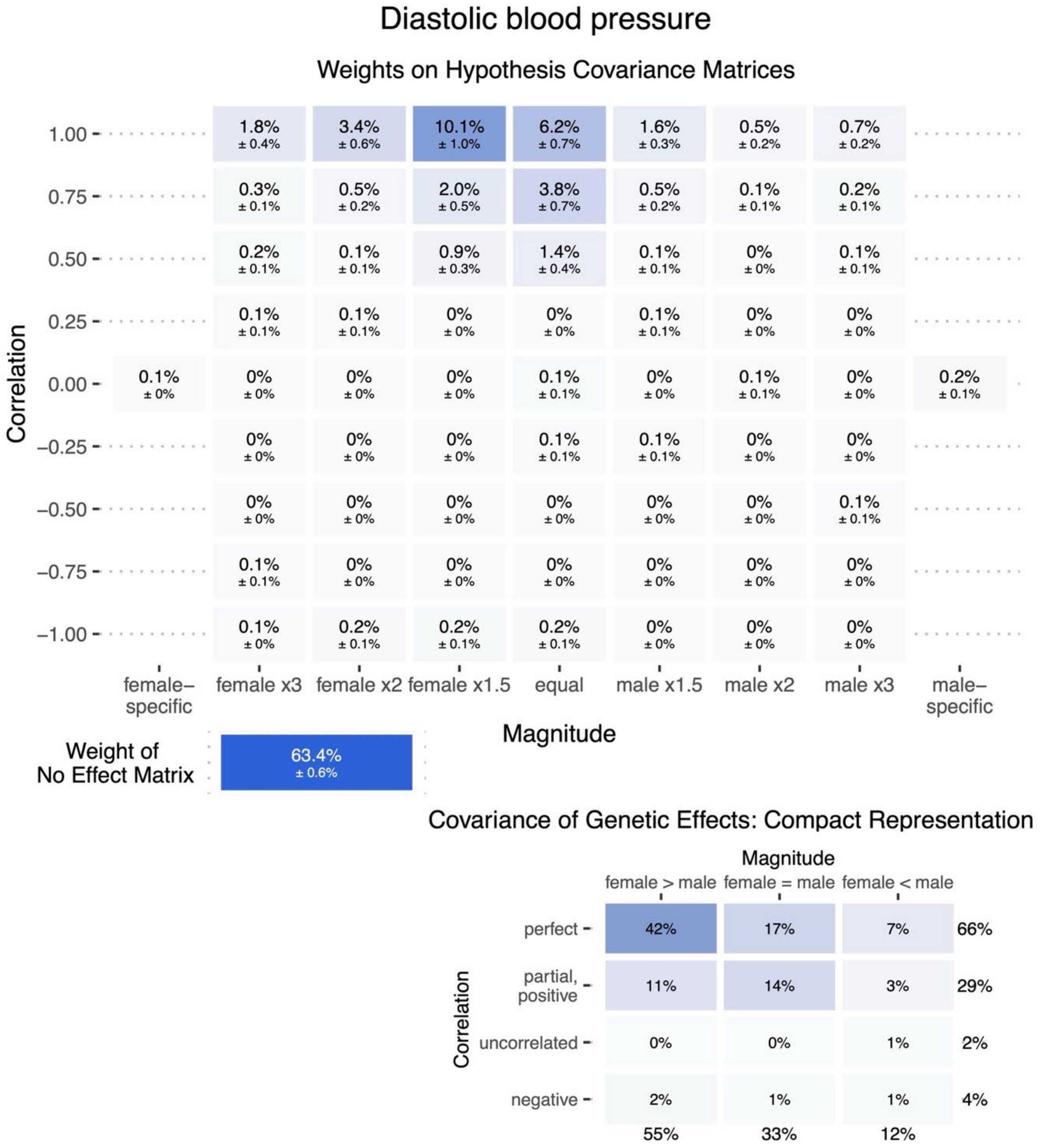

Data S8: Eosinophil percentage

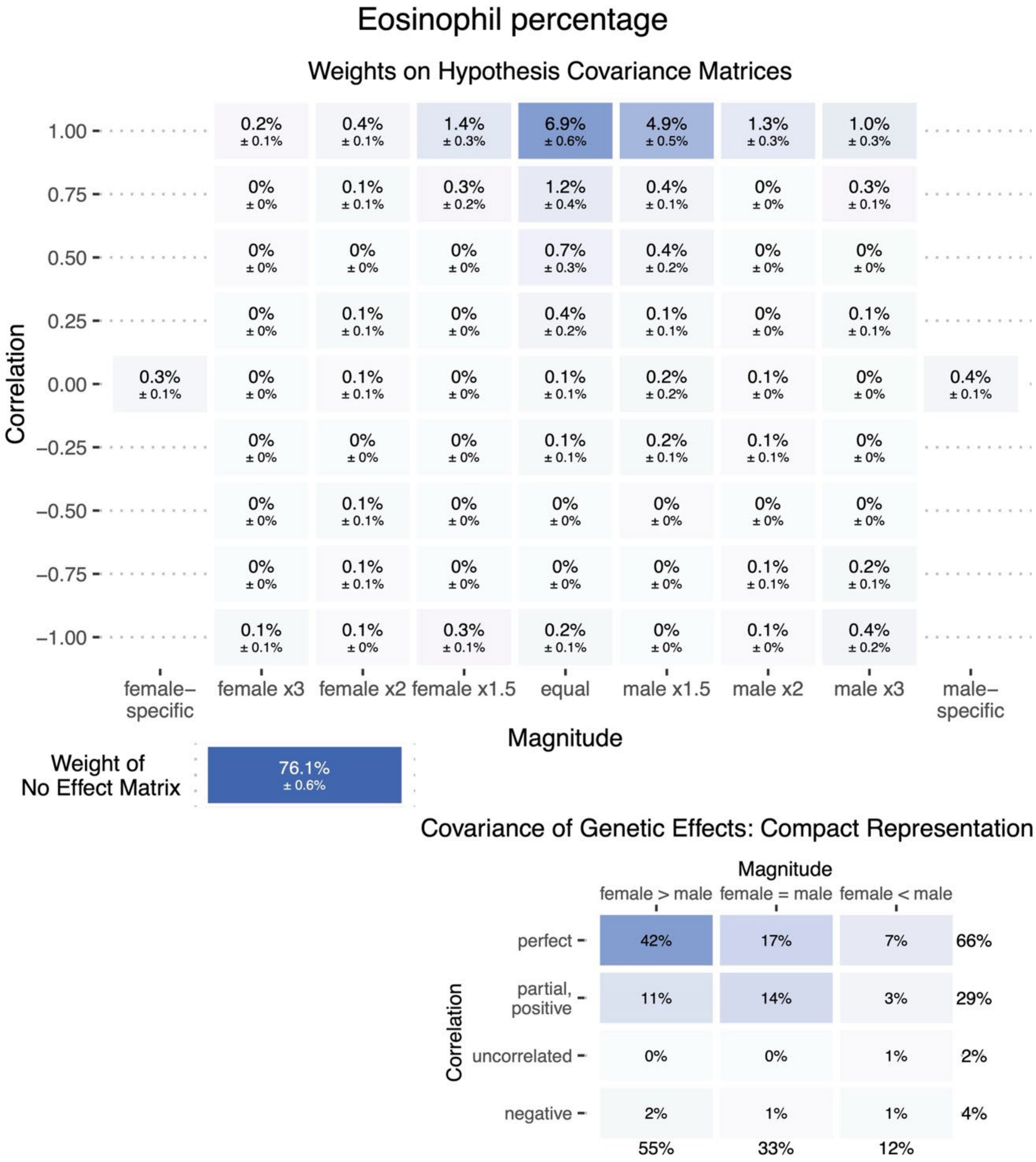

Data S9: Forced vital capacity

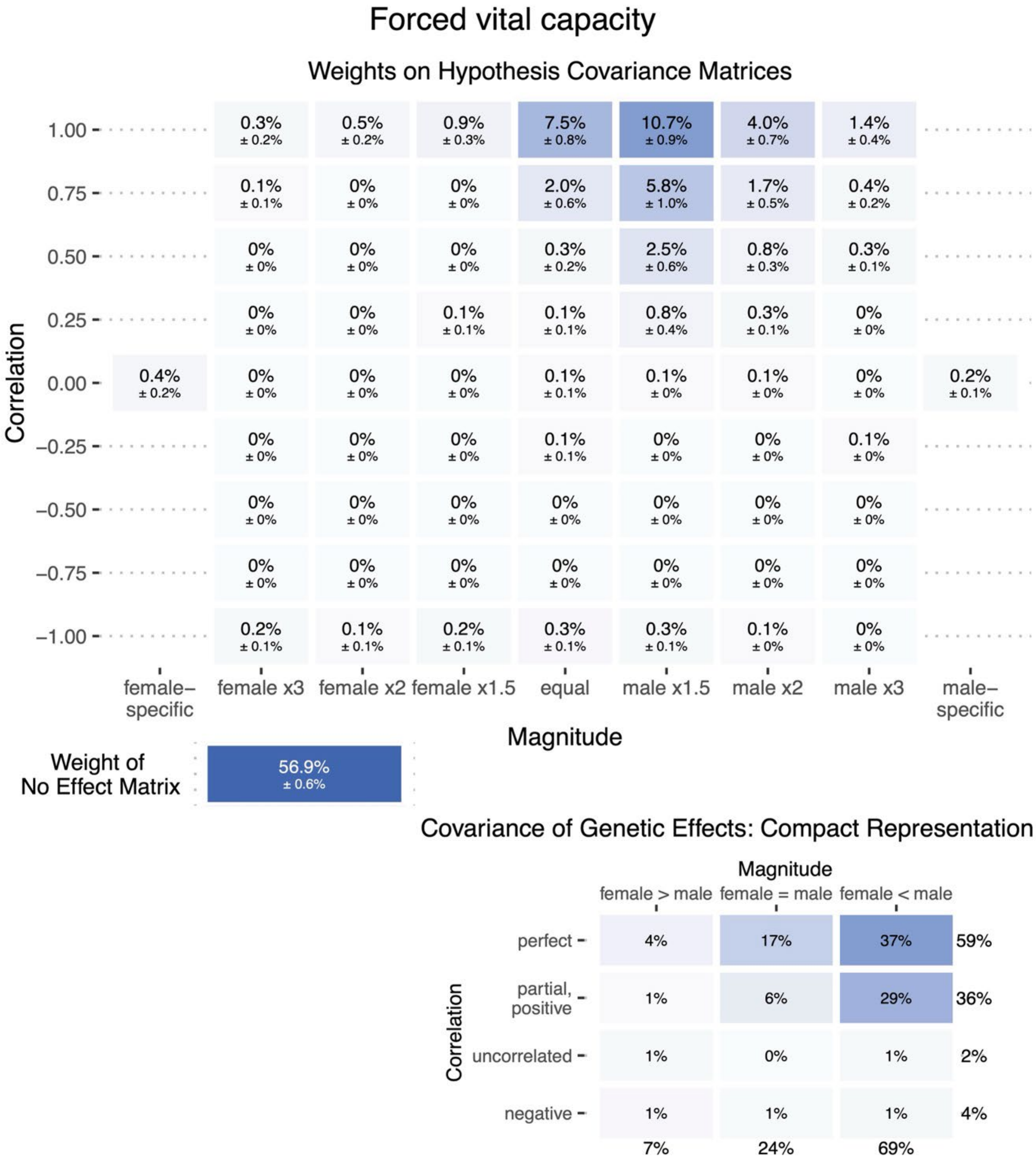

Data S10: HbA1c

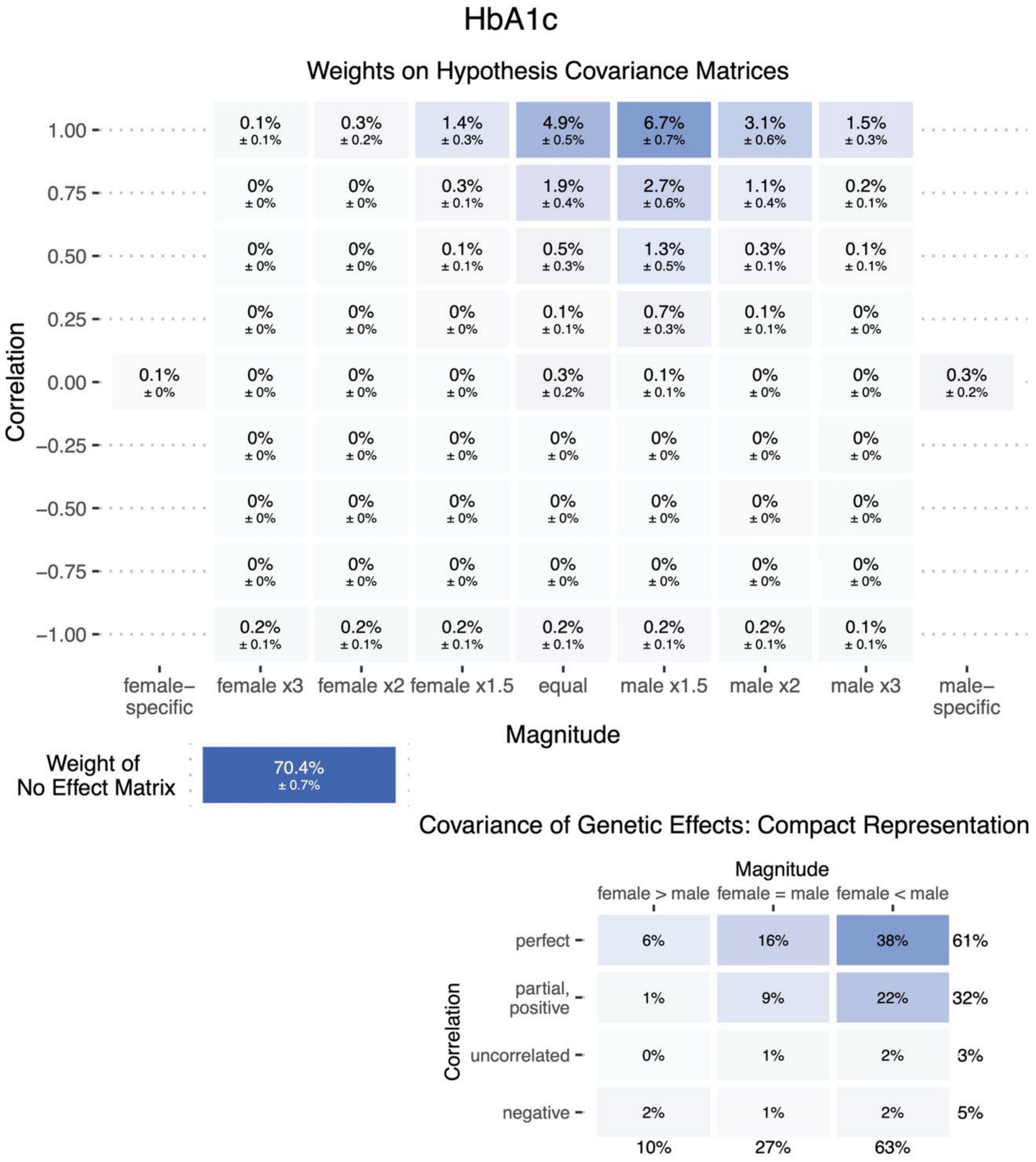

Data S11: Height

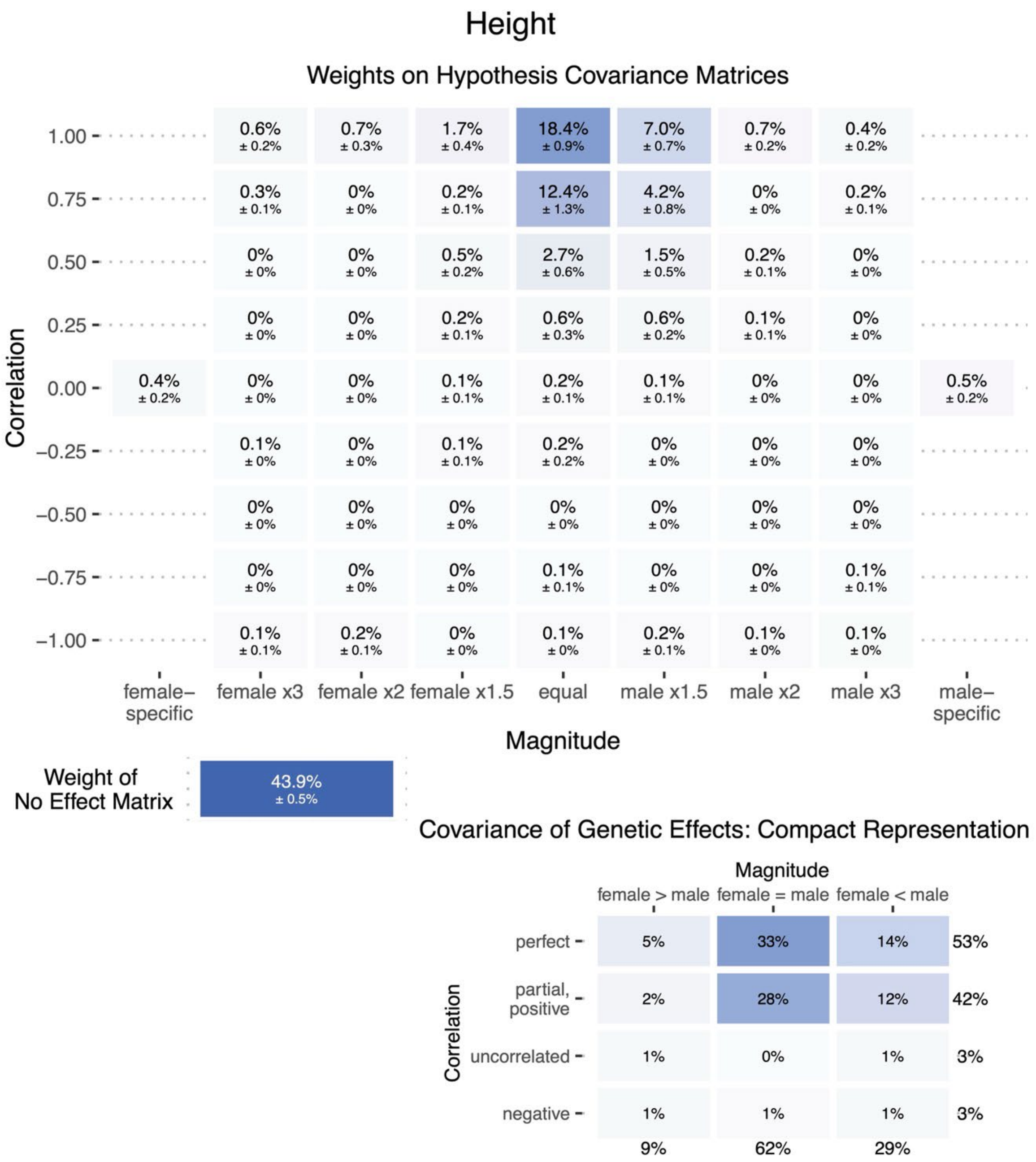

Data S12: Hip circumference

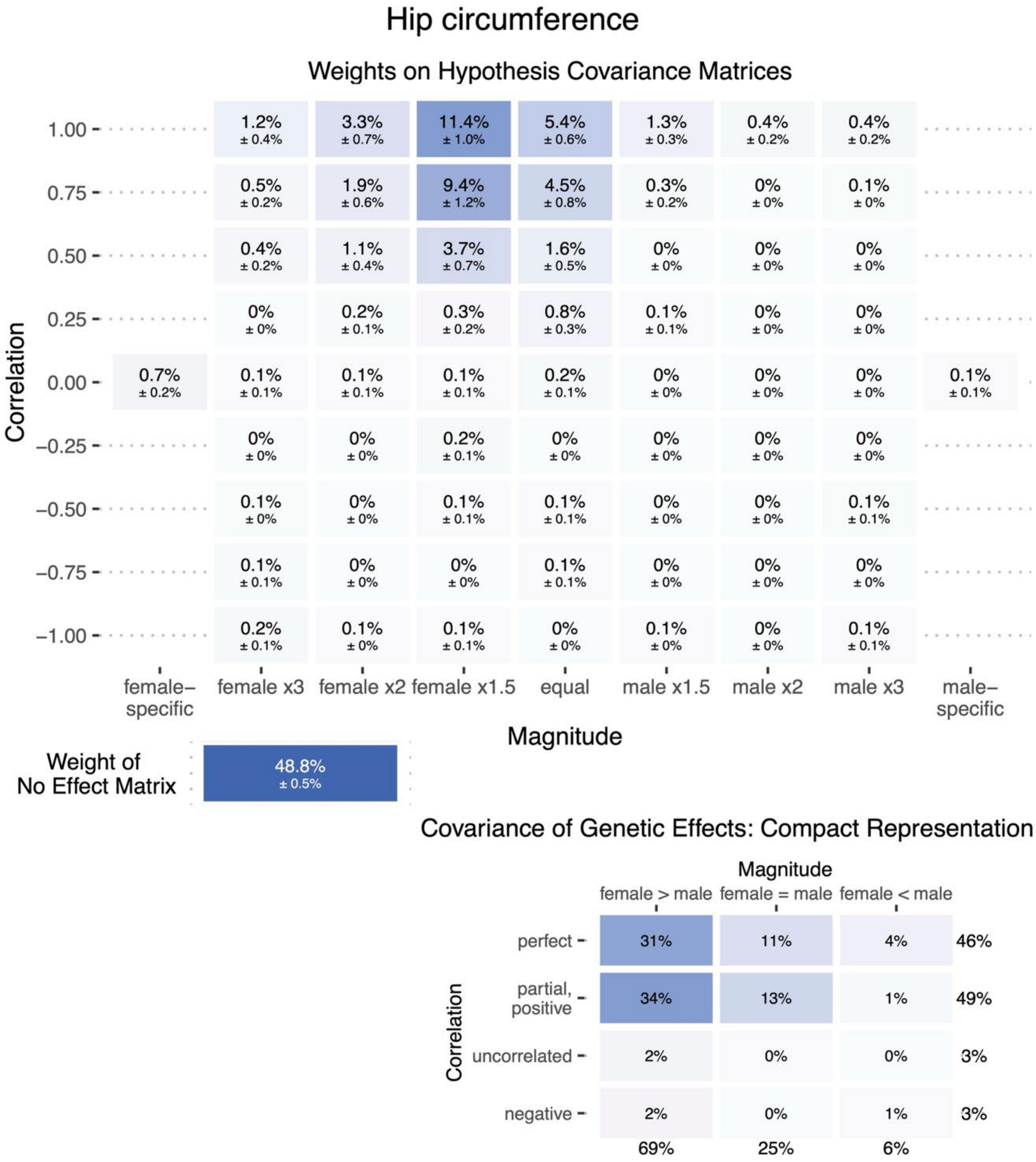

Data S13: IGF-1

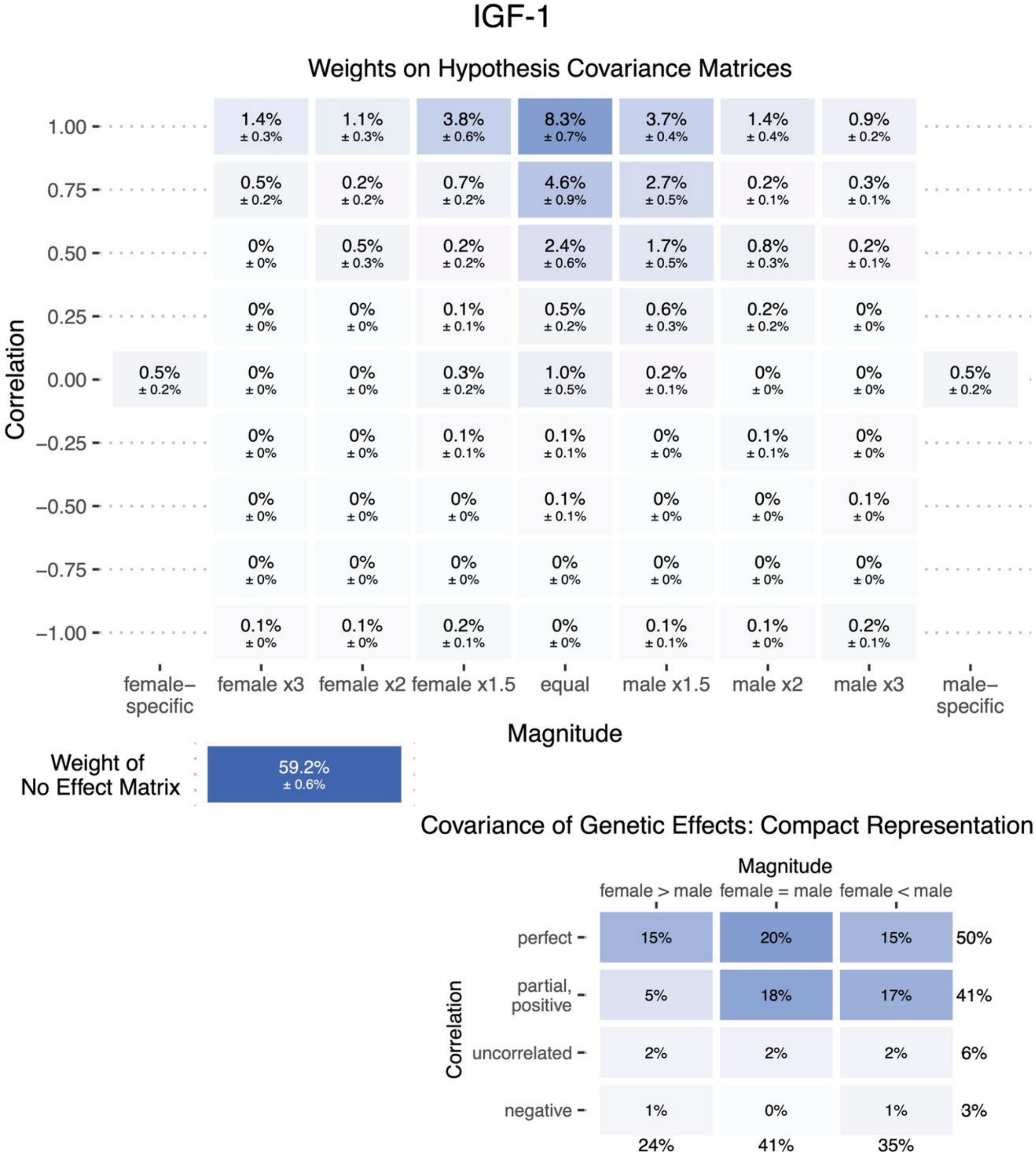

Data S14: Lymphocyte percentage

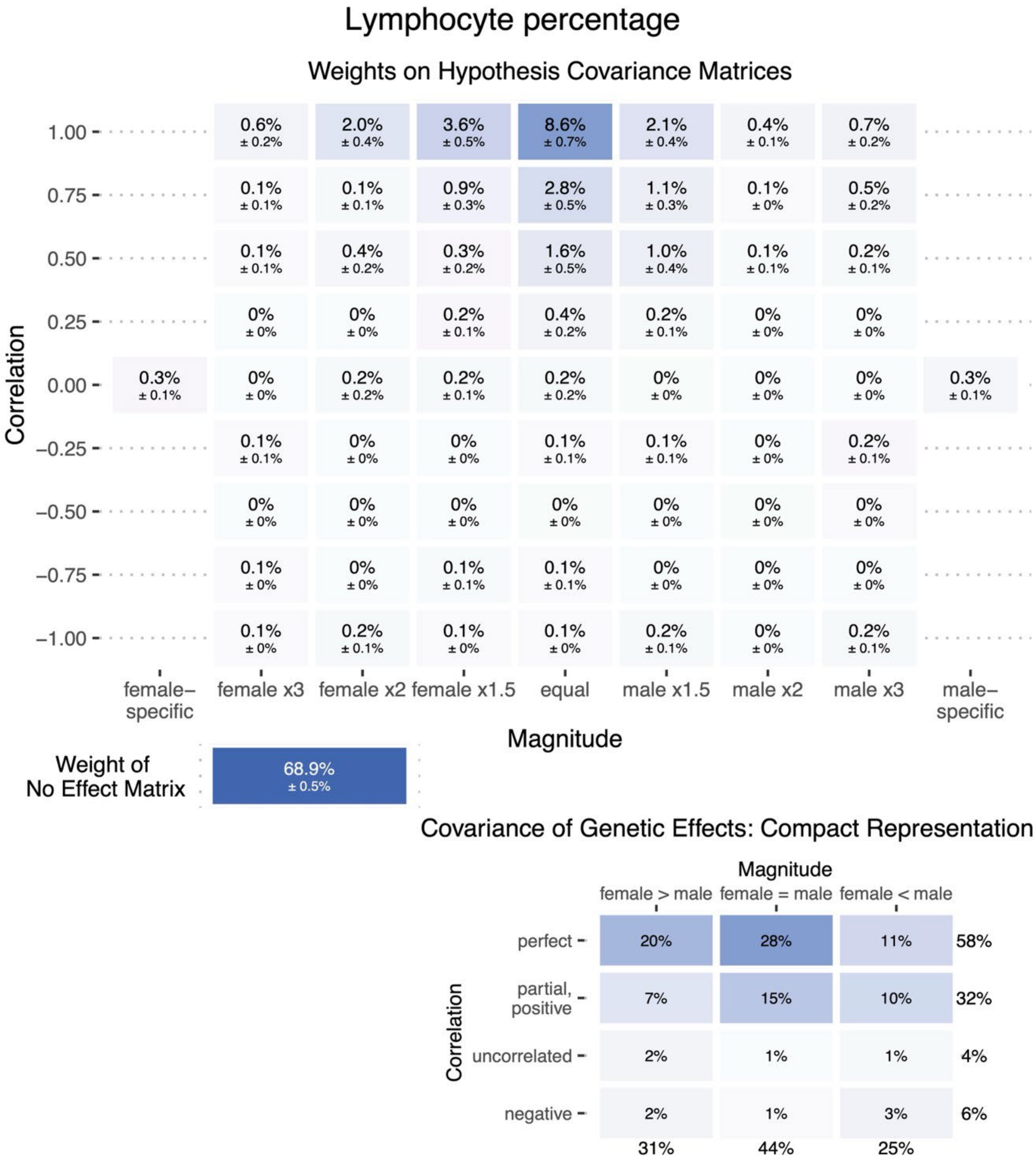

Data S15: Total protein

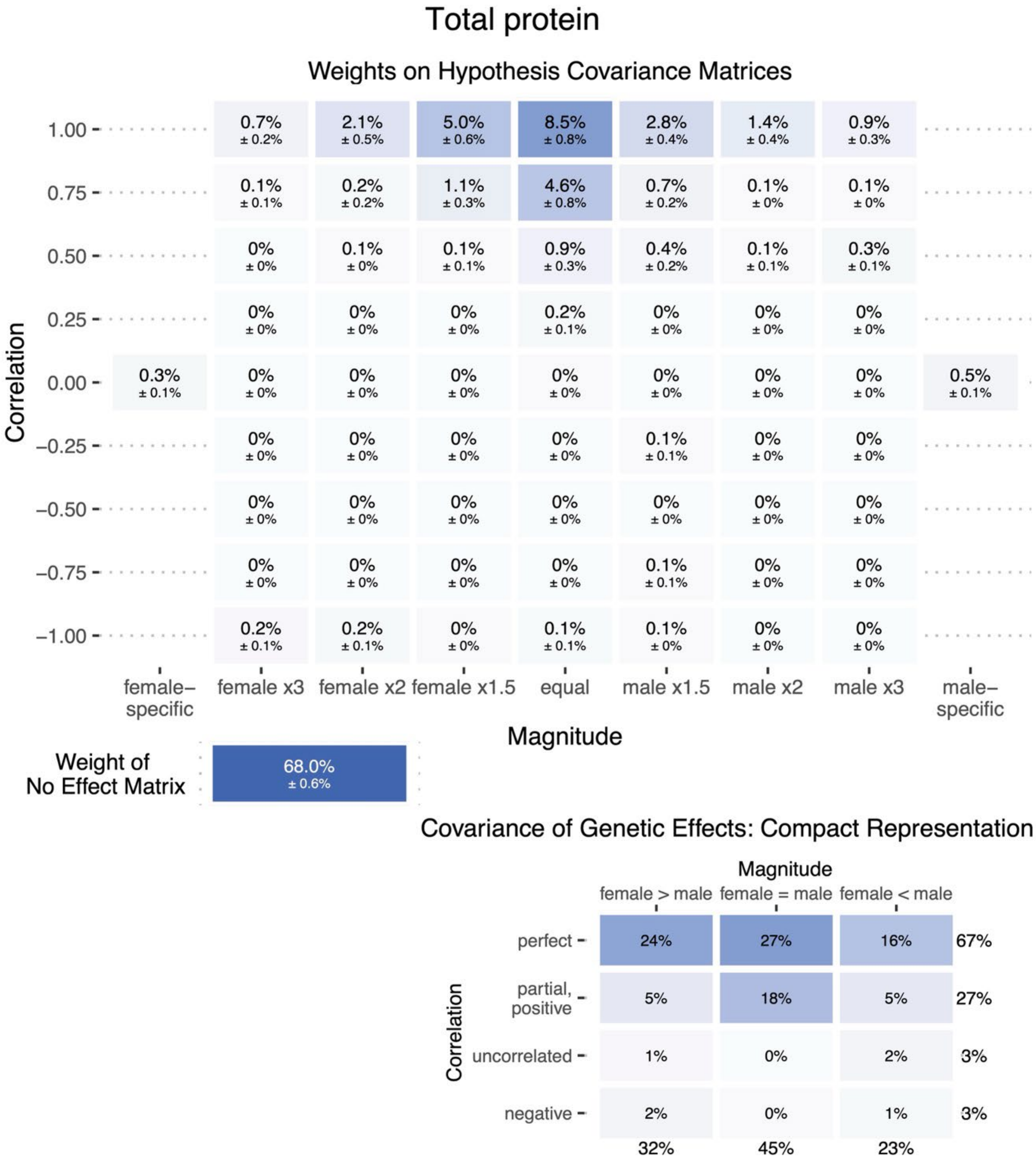

Data S16: Pulse rate

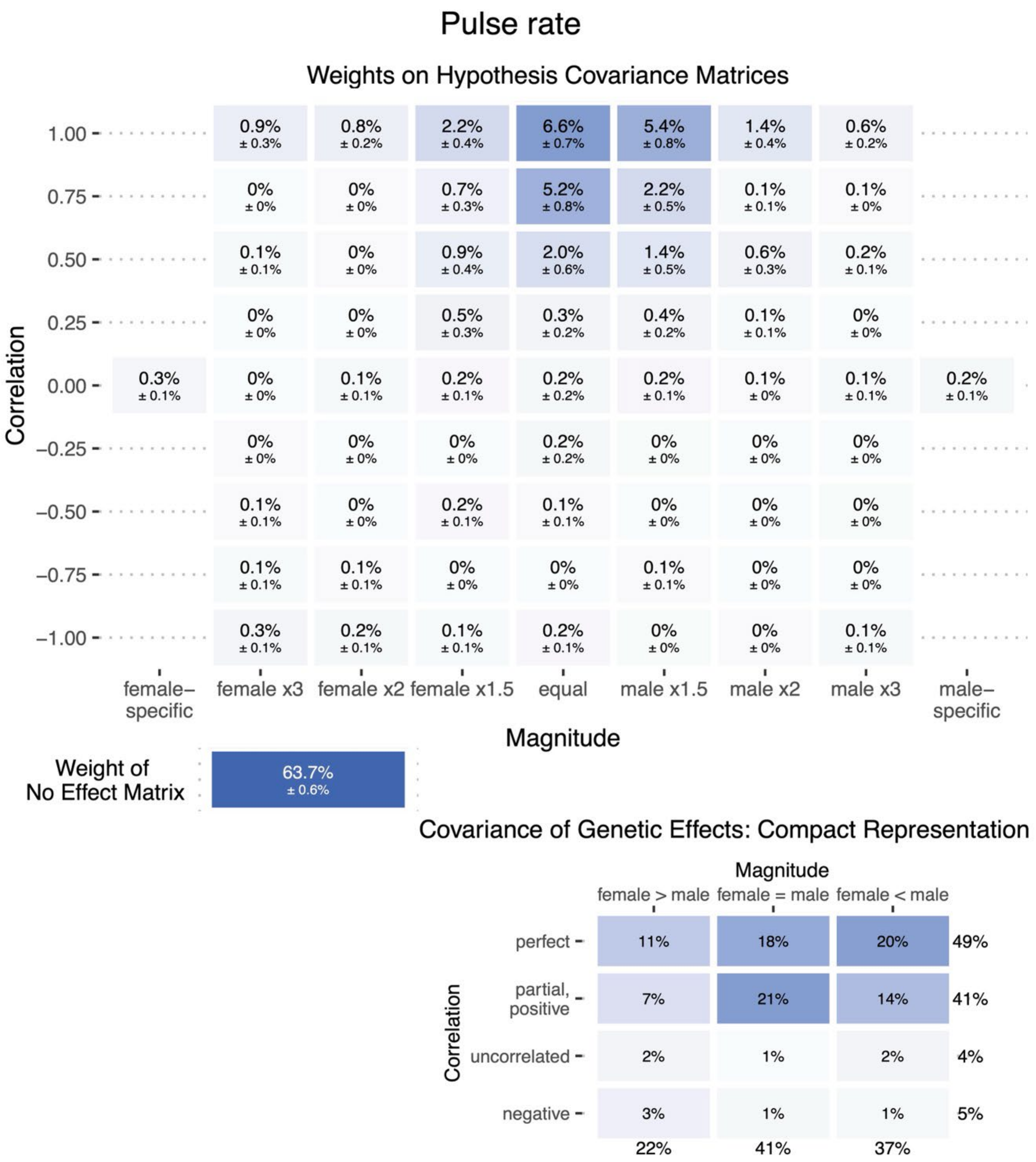

Data S17: Red blood cell count

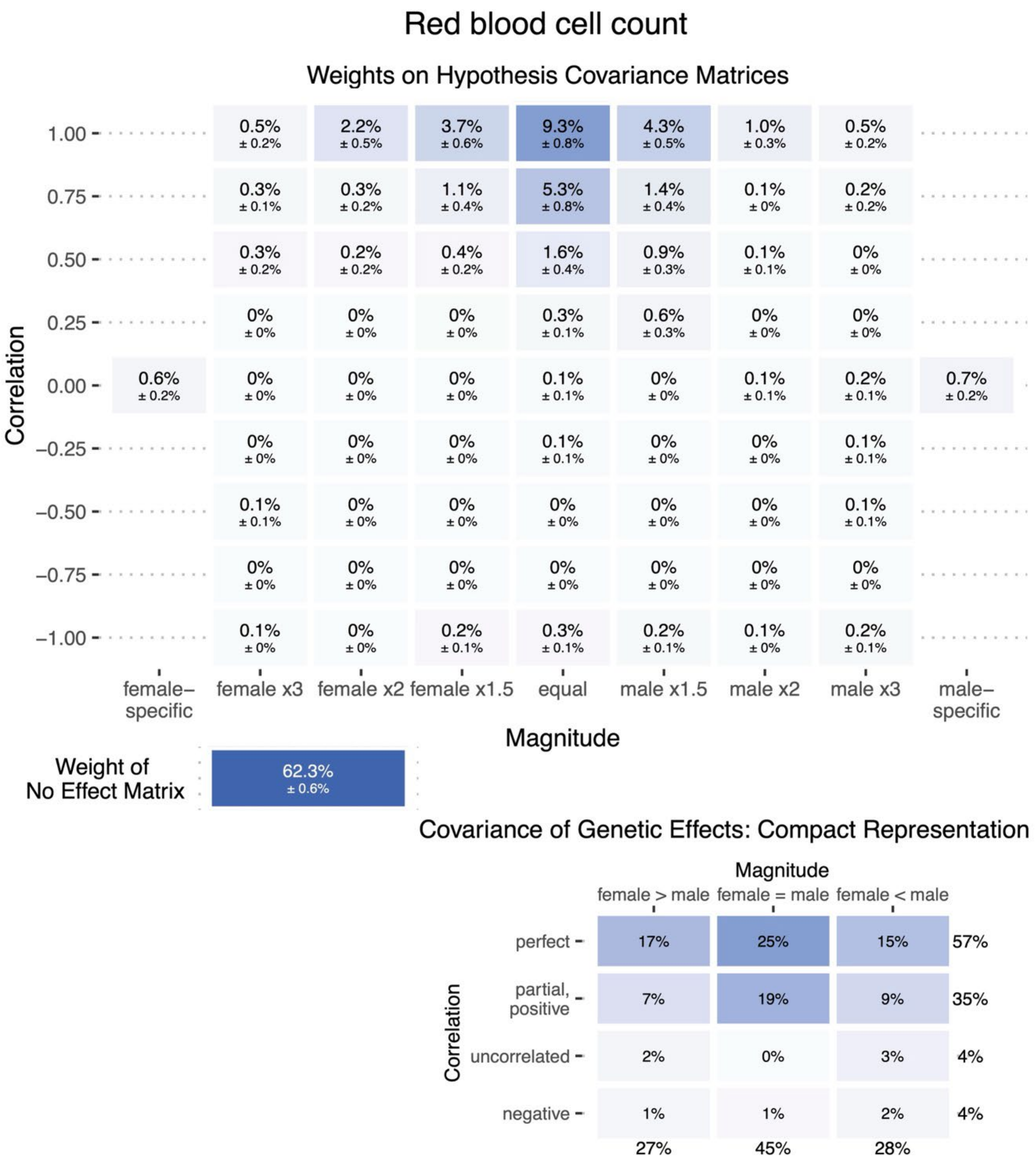

Data S18: SHBG

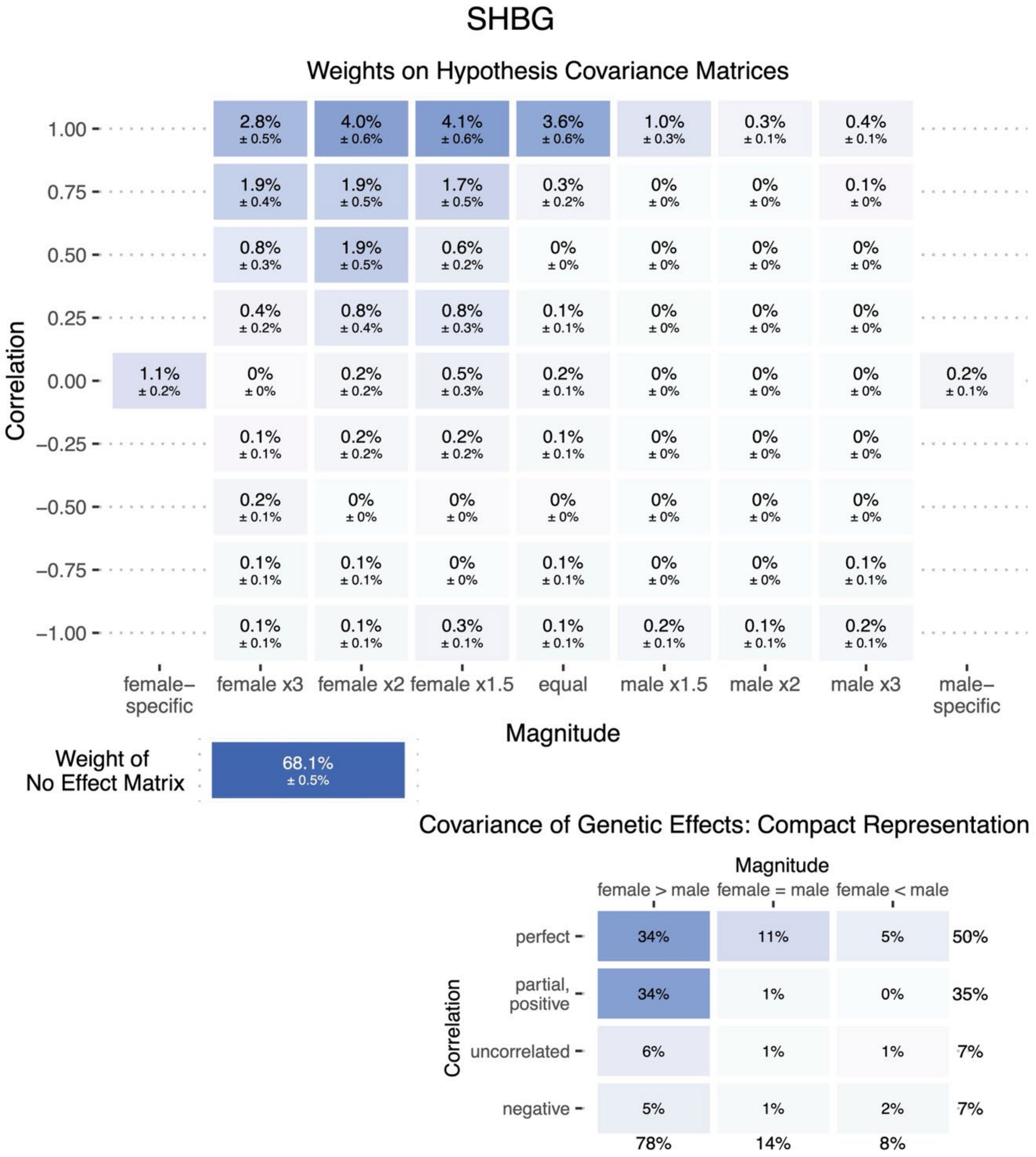

Data S19: Systolic blood pressure

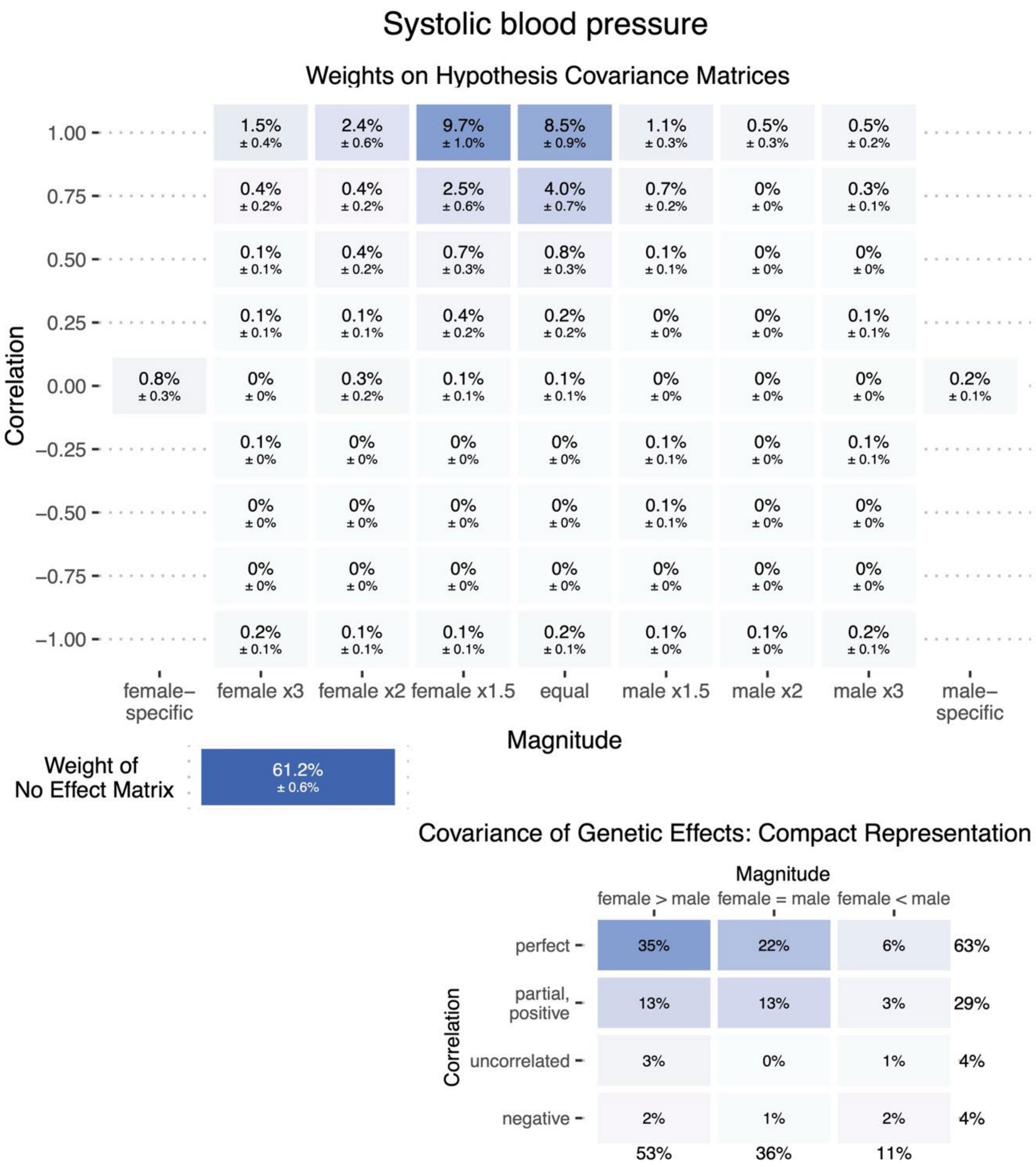

Data S20: Testosterone

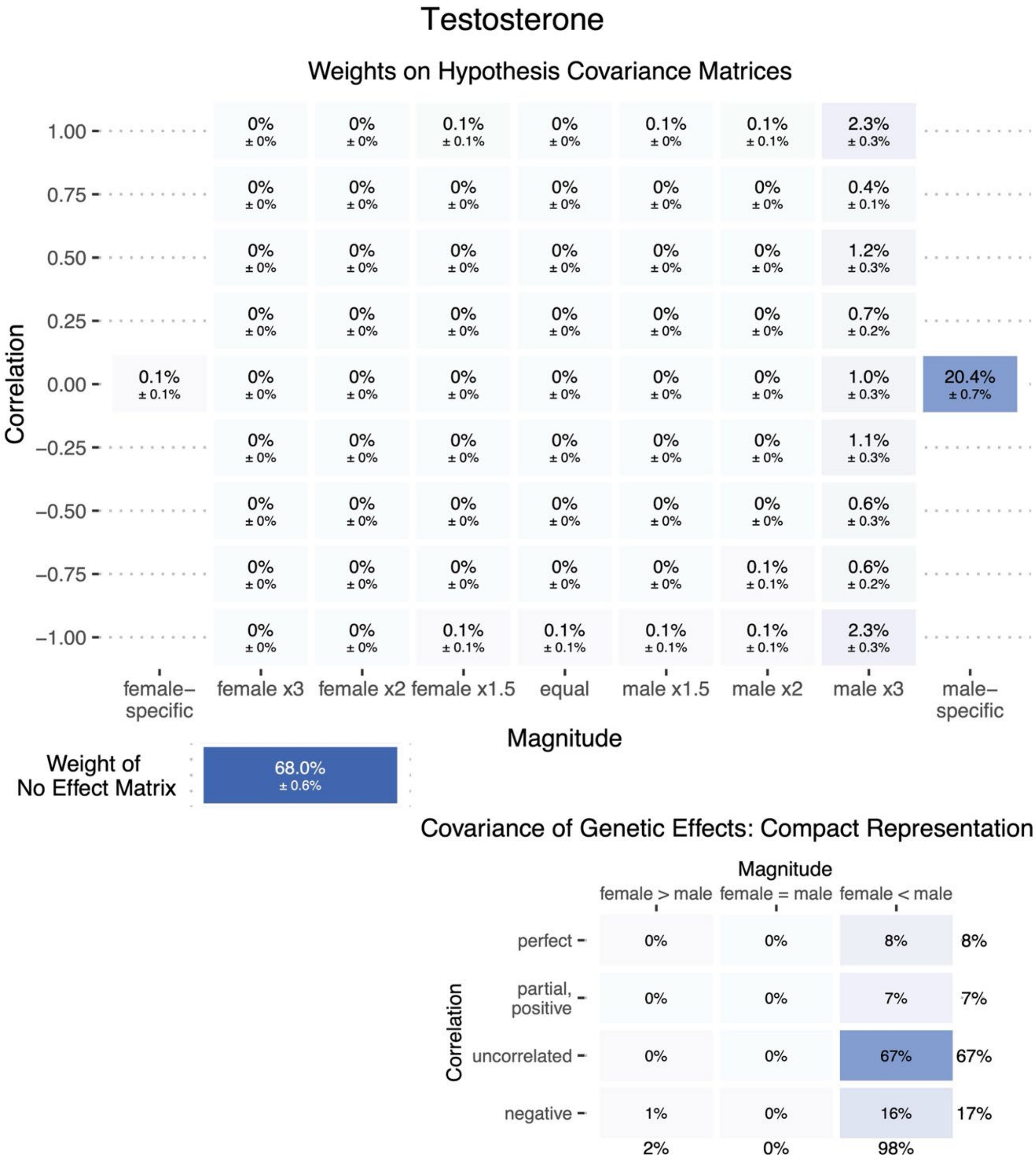

Data S21: Urate

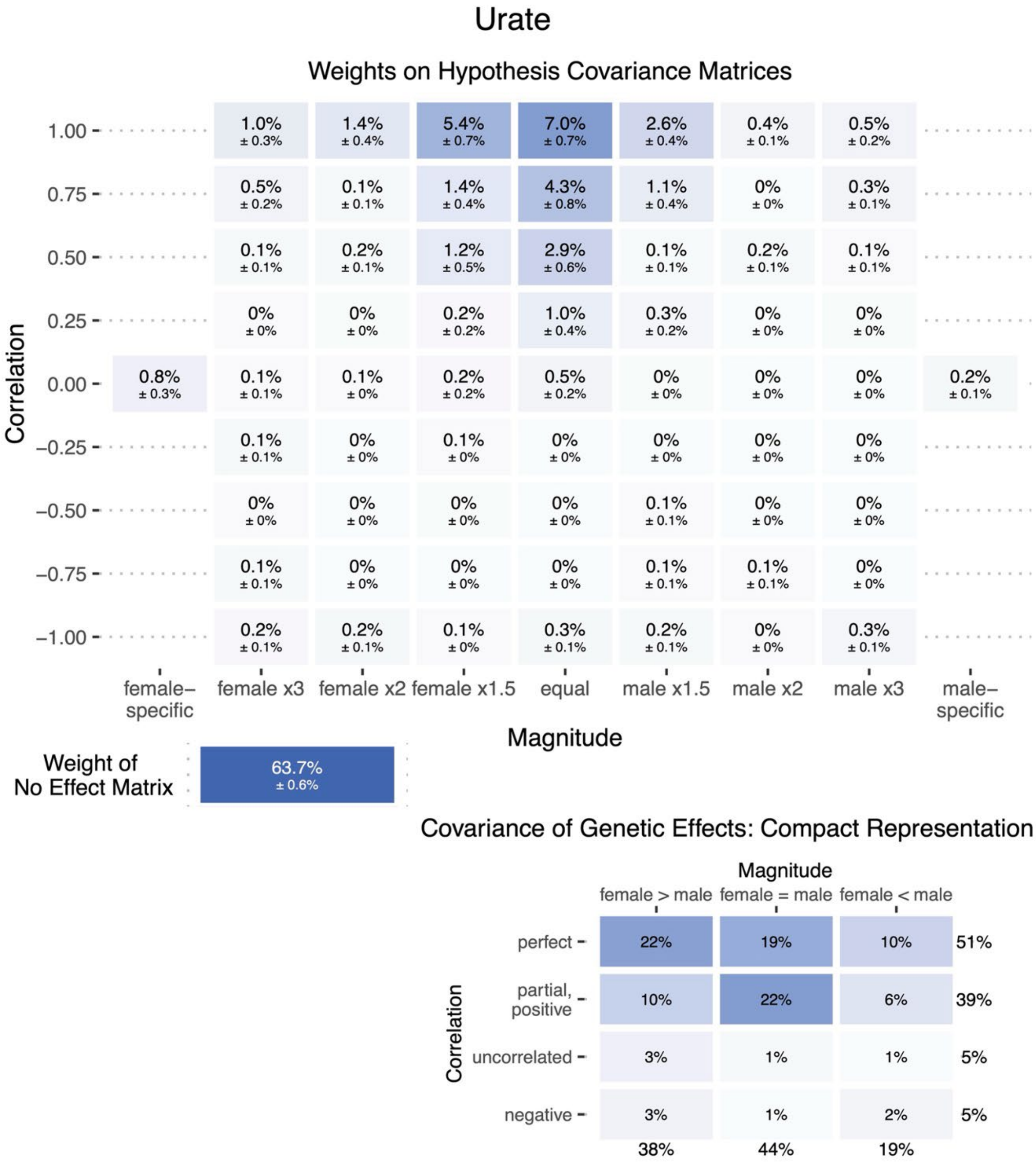

Data S22: Urea

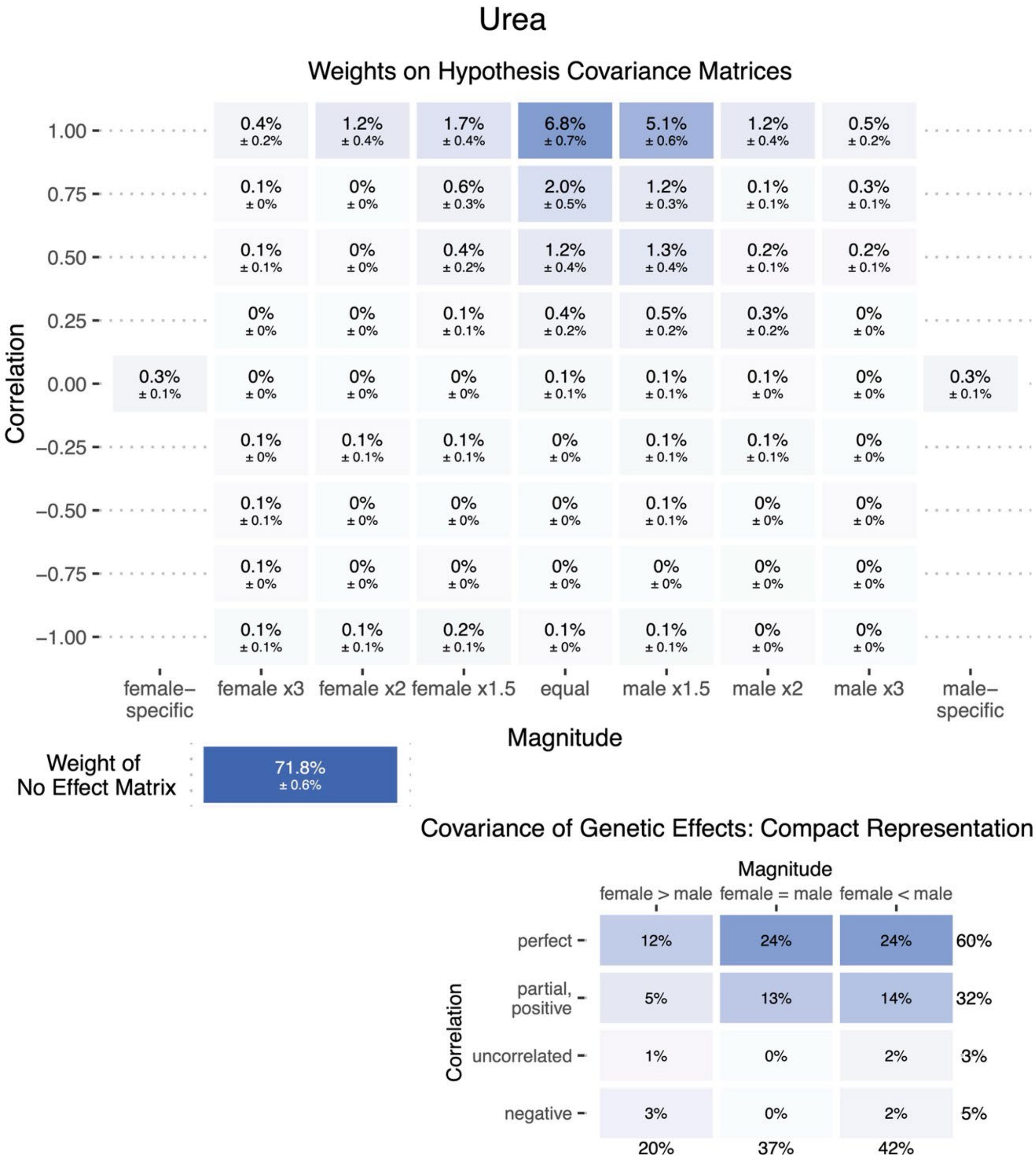

Data S23: Waist circumference

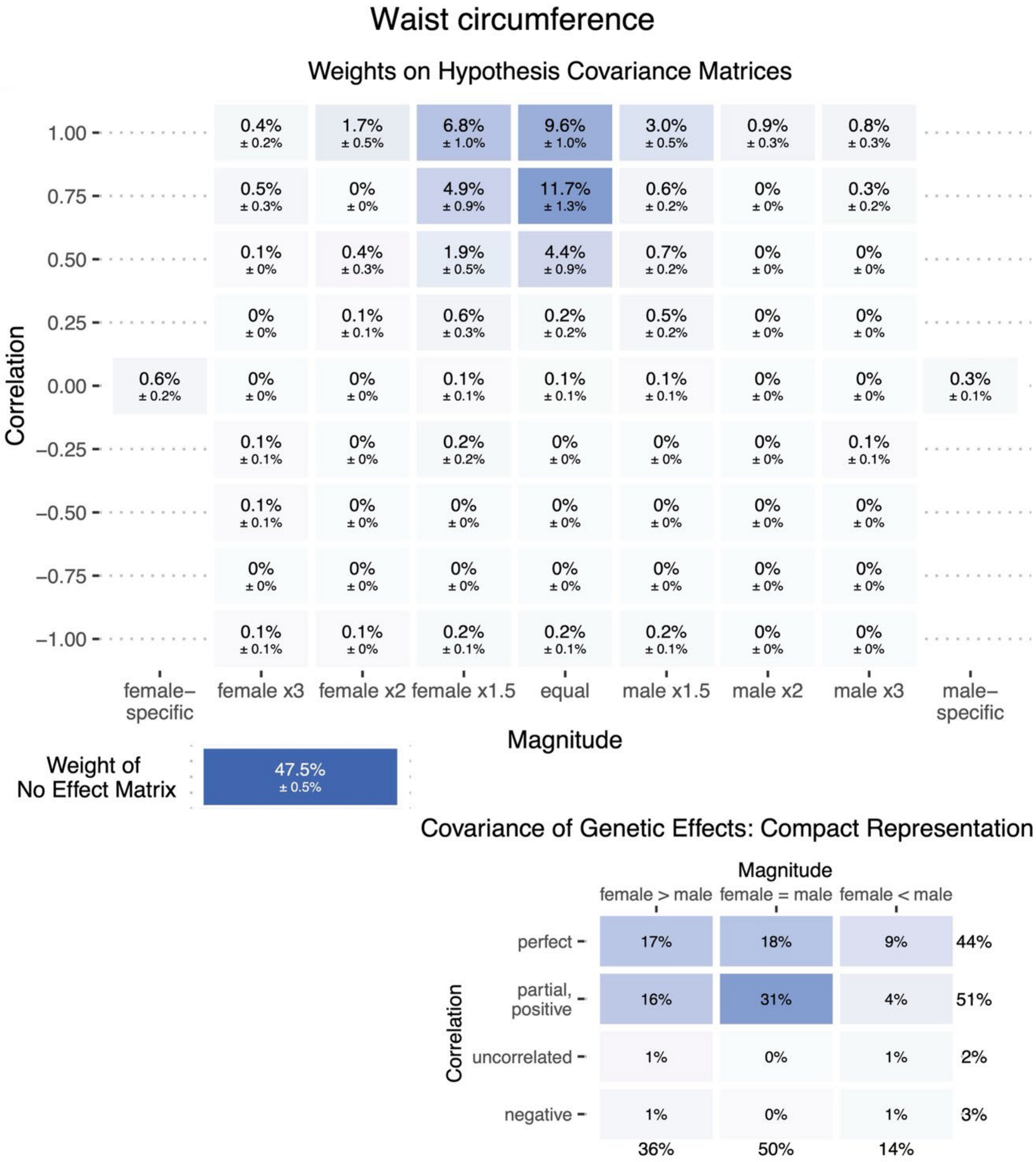

Data S24: Waist to hip ratio

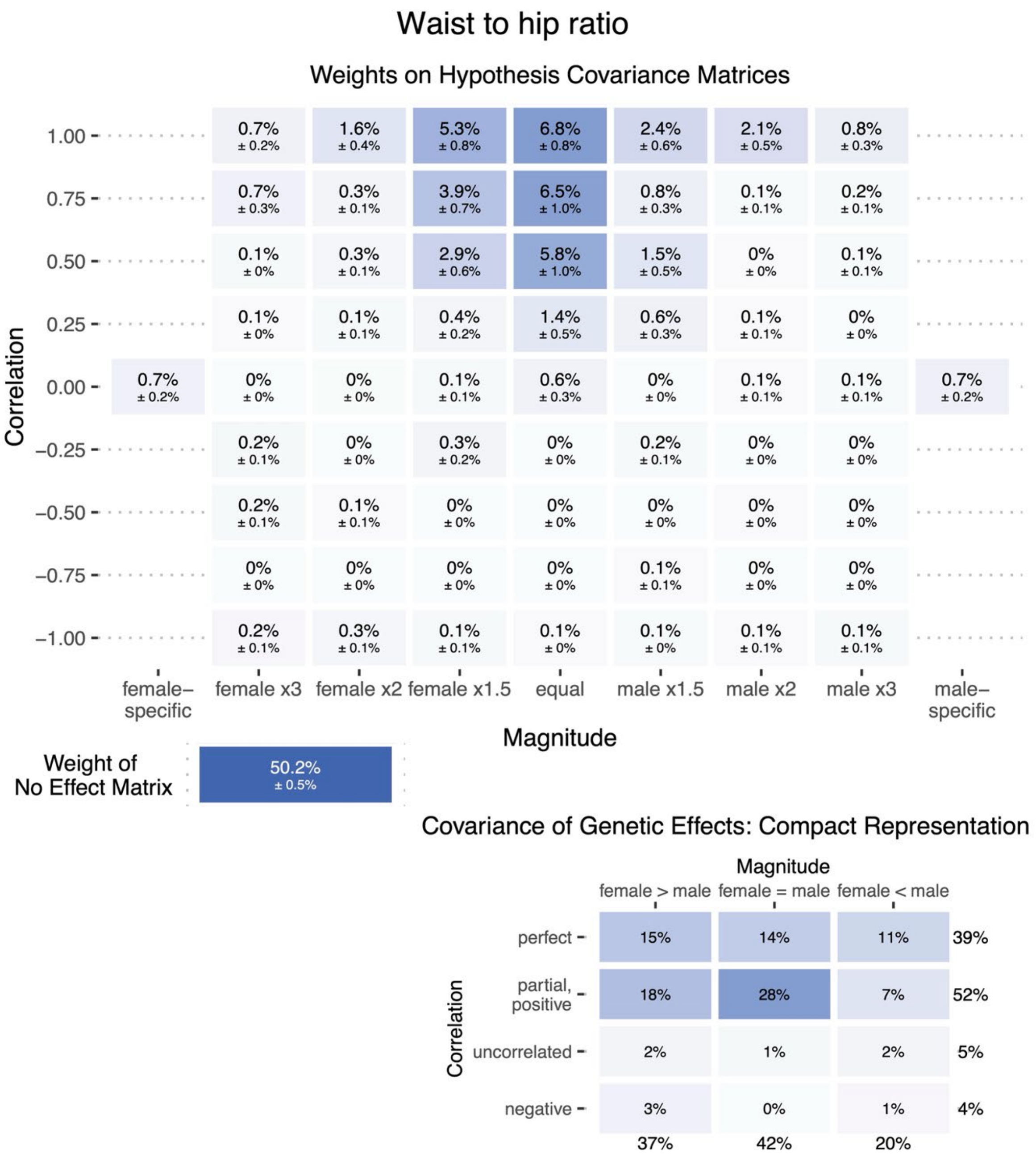

Data S25: Weight

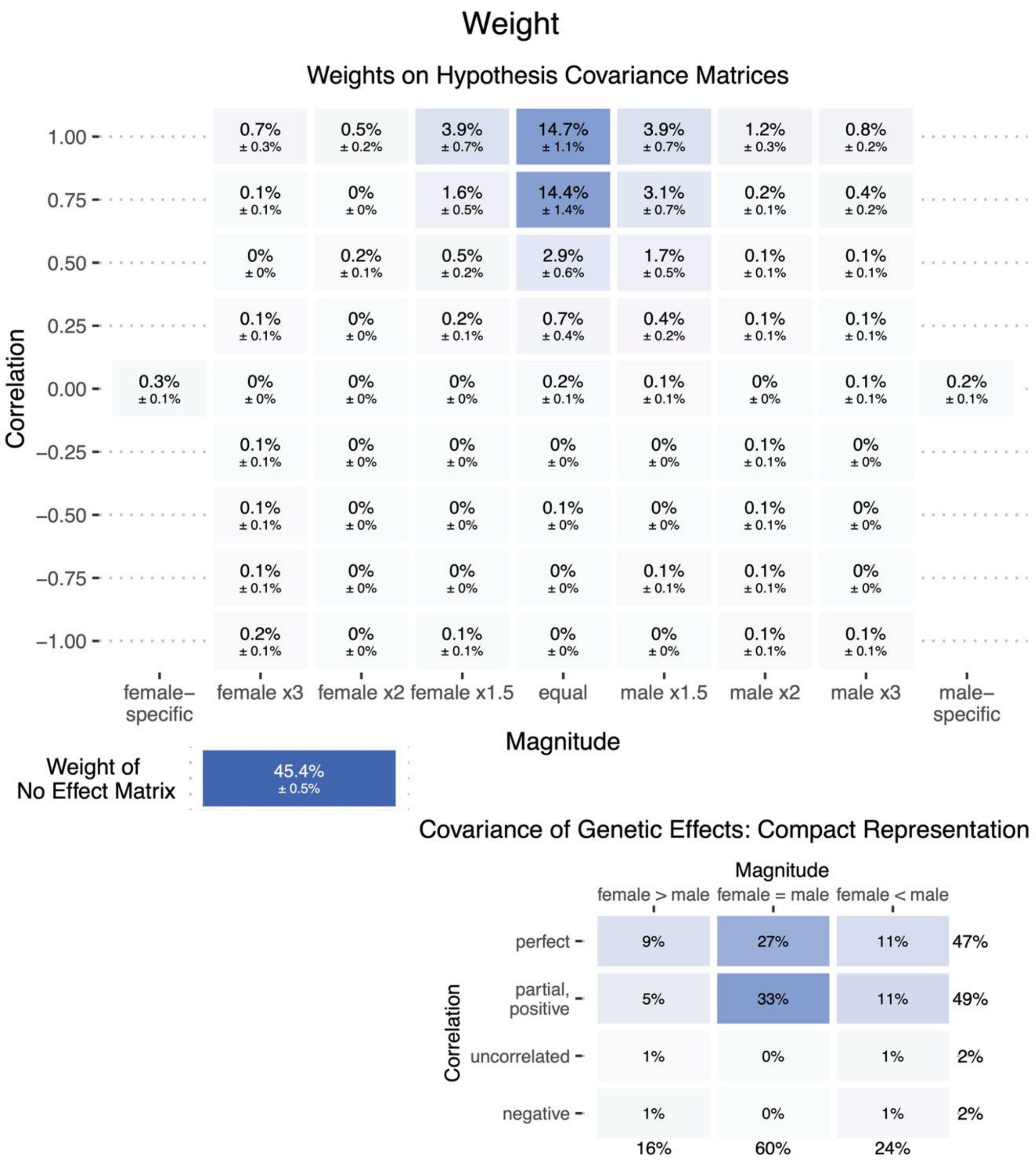

Correlation	female-specific	female x3	female x2	female x1.5	equal	male x1.5	male x2	male x3	male-specific
1.00		0.7% ± 0.3%	0.5% ± 0.2%	3.9% ± 0.7%	14.7% ± 1.1%	3.9% ± 0.7%	1.2% ± 0.3%	0.8% ± 0.2%	
0.75		0.1% ± 0.1%	0% ± 0%	1.6% ± 0.5%	14.4% ± 1.4%	3.1% ± 0.7%	0.2% ± 0.1%	0.4% ± 0.2%	
0.50		0% ± 0%	0.2% ± 0.1%	0.5% ± 0.2%	2.9% ± 0.6%	1.7% ± 0.5%	0.1% ± 0.1%	0.1% ± 0.1%	
0.25		0.1% ± 0.1%	0% ± 0%	0.2% ± 0.1%	0.7% ± 0.4%	0.4% ± 0.2%	0.1% ± 0.1%	0.1% ± 0.1%	
0.00	0.3% ± 0.1%	0% ± 0%	0% ± 0%	0% ± 0%	0.2% ± 0.1%	0.1% ± 0.1%	0% ± 0%	0.1% ± 0.1%	0.2% ± 0.1%
−0.25		0.1% ± 0.1%	0% ± 0%	0% ± 0%	0% ± 0%	0% ± 0%	0.1% ± 0.1%	0% ± 0%	
−0.50		0.1% ± 0.1%	0% ± 0%	0% ± 0%	0.1% ± 0%	0% ± 0%	0.1% ± 0.1%	0% ± 0%	
−0.75		0.1% ± 0.1%	0% ± 0%	0% ± 0%	0% ± 0%	0.1% ± 0.1%	0.1% ± 0.1%	0% ± 0%	
−1.00		0.2% ± 0.1%	0% ± 0%	0.1% ± 0.1%	0% ± 0%	0% ± 0%	0.1% ± 0.1%	0.1% ± 0.1%	

Correlation	female > male	female = male	female < male	
perfect	9%	27%	11%	47%
partial, positive	5%	33%	11%	49%
uncorrelated	1%	0%	1%	2%
negative	1%	0%	1%	2%
	16%	60%	24%	

Data S26: Whole body fat mass

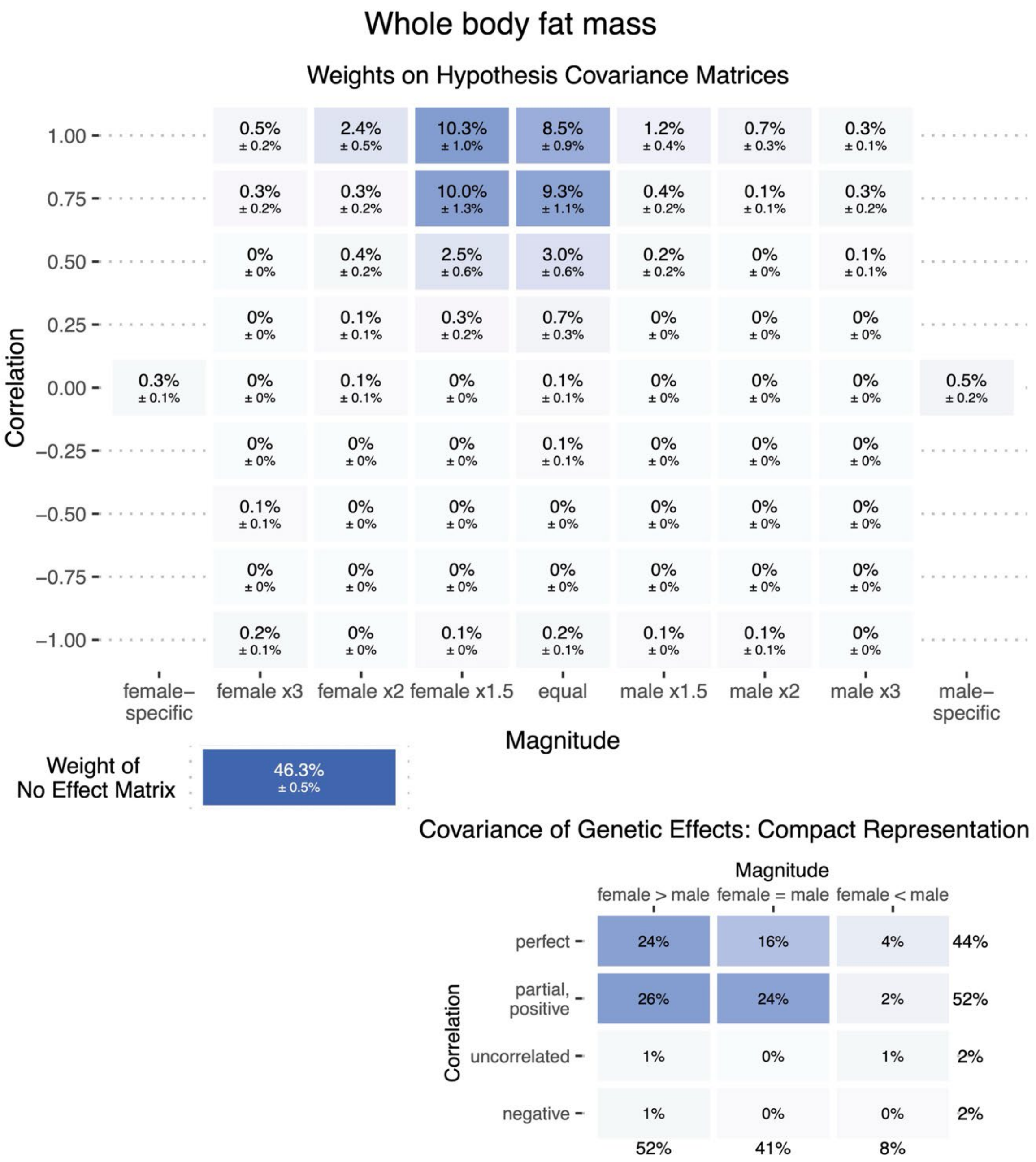

Data S27: Waist:hip (BMI adjusted)

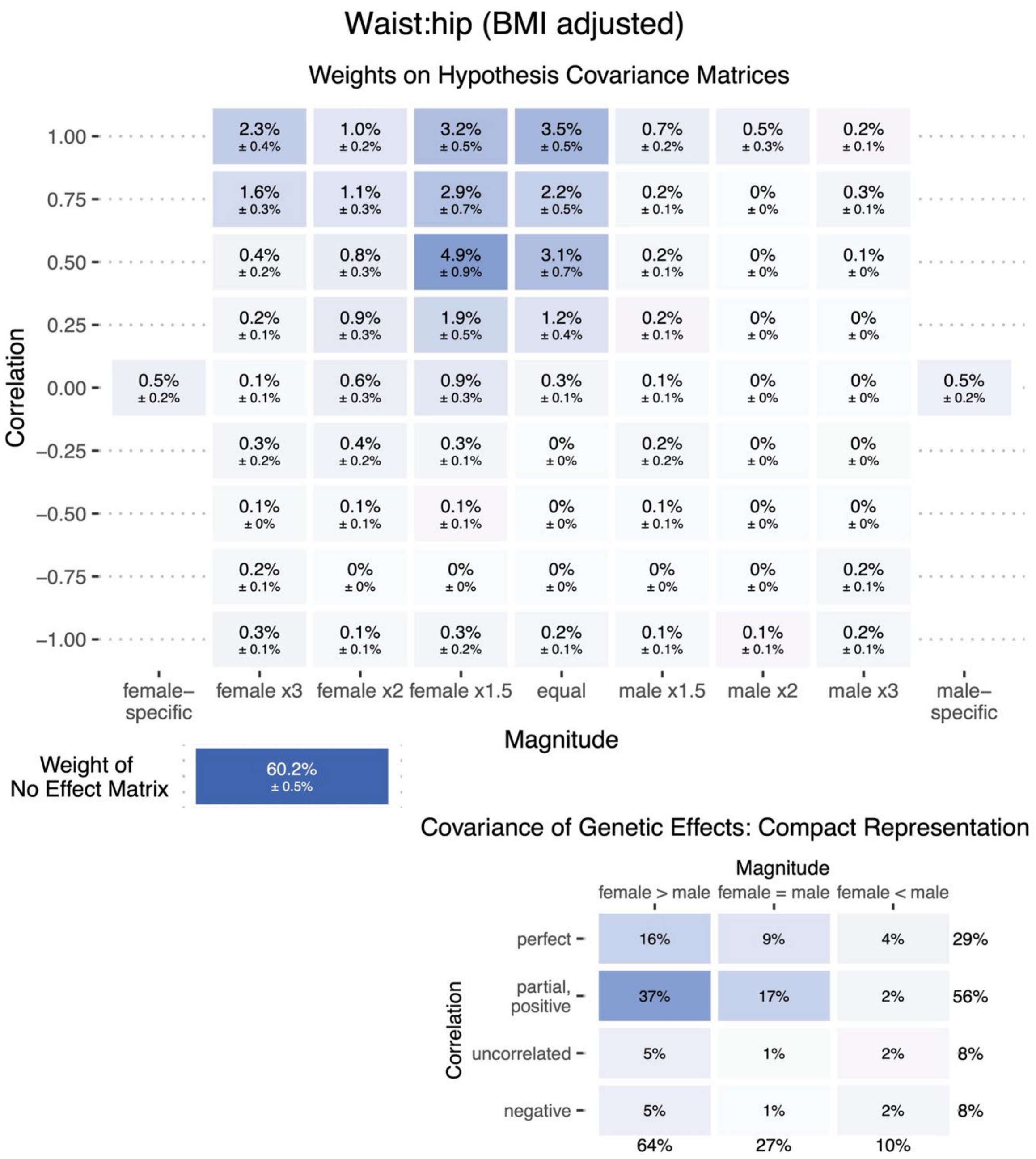

References

1. Purcell, S., and Chang, C. (2020). PLINK 2.00 alpha.
2. Chang, C.C., Chow, C.C., Tellier, L.C., Vattikuti, S., Purcell, S.M., and Lee, J.J. (2015). Second-generation PLINK: rising to the challenge of larger and richer datasets. Gigascience *4*, 7. 10.1186/s13742-015-0047-8.
3. Graffelman, J., and Moreno, V. (2013). The mid p-value in exact tests for Hardy-Weinberg equilibrium. Stat Appl Genet Mol Biol *12*, 433–448. 10.1515/sagmb-2012-0039.
4. Graffelman, J., and Weir, B.S. (2016). Testing for Hardy-Weinberg equilibrium at biallelic genetic markers on the X chromosome. Heredity (Edinb) *116*, 558–568. 10.1038/hdy.2016.20.
5. Wigginton, J.E., Cutler, D.J., and Abecasis, G.R. (2005). A note on exact tests of Hardy-Weinberg equilibrium. Am J Hum Genet *76*, 887–893. 10.1086/429864.
6. Purcell, S., Neale, B., Todd-Brown, K., Thomas, L., Ferreira, M.A.R., Bender, D., Maller, J., Sklar, P., de Bakker, P.I.W., Daly, M.J., et al. (2007). PLINK: a tool set for whole-genome association and population-based linkage analyses. Am J Hum Genet *81*, 559–575. 10.1086/519795.
7. Abbot, L., Bryant, S., Churchhouse, C., Ganna, A., Howrigan, D., Palmer, D., Neale, B., Walters, R., Carey, C., and The Hail Team (2017). GWAS Results. http://www.nealelab.is/uk-biobank/.
8. Bulik-Sullivan, B.K., Loh, P.-R., Finucane, H.K., Ripke, S., Yang, J., Patterson, N., Daly, M.J., Price, A.L., and Neale, B.M. (2015). LD Score regression distinguishes confounding from polygenicity in genome-wide association studies. Nat Genet *47*, 291–295. 10.1038/ng.3211.
9. McLaren, W., Gil, L., Hunt, S.E., Riat, H.S., Ritchie, G.R.S., Thormann, A., Flicek, P., and Cunningham, F. (2016). The Ensembl Variant Effect Predictor. Genome Biol *17*, 122. 10.1186/s13059-016-0974-4.
10. Bulik-Sullivan, B.K., Finucane, H.K., Anttila, V., Gusev, A., Day, F.R., Loh, P.-R., Duncan, L., Perry, J.R.B., Patterson, N., Robinson, E.B., et al. (2015). An atlas of genetic correlations across human diseases and traits. Nat Genet *47*, 1236–1241. 10.1038/ng.3406.
11. Choi, S.W., Mak, T.S.-H., and O'Reilly, P.F. (2020). Tutorial: a guide to performing polygenic risk score analyses. Nat Protoc *15*, 2759–2772. 10.1038/s41596-020-0353-1.
12. Berisa, T., and Pickrell, J.K. (2016). Approximately independent linkage disequilibrium blocks in human populations. Bioinformatics *32*, 283–285. 10.1093/bioinformatics/btv546.

13. Auton, A., Abecasis, G.R., Altshuler, D.M., Durbin, R.M., Abecasis, G.R., Bentley, D.R., Chakravarti, A., Clark, A.G., Donnelly, P., Eichler, E.E., et al. (2015). A global reference for human genetic variation. Nature *526*, 68–74. 10.1038/nature15393.

14. Karczewski, K.J., Francioli, L.C., Tiao, G., Cummings, B.B., Alföldi, J., Wang, Q., Collins, R.L., Laricchia, K.M., Ganna, A., Birnbaum, D.P., et al. (2020). The mutational constraint spectrum quantified from variation in 141,456 humans. Nature *581*, 434–443. 10.1038/s41586-020-2308-7.

15. Kent, W.J., Sugnet, C.W., Furey, T.S., Roskin, K.M., Pringle, T.H., Zahler, A.M., and Haussler, and D. (2002). The Human Genome Browser at UCSC. Genome Res *12*, 996–1006. 10.1101/gr.229102.

16. Danecek, P., Auton, A., Abecasis, G., Albers, C.A., Banks, E., DePristo, M.A., Handsaker, R.E., Lunter, G., Marth, G.T., Sherry, S.T., et al. (2011). The variant call format and VCFtools. Bioinformatics *27*, 2156–2158. 10.1093/bioinformatics/btr330.

17. Pirastu, N., Cordioli, M., Nandakumar, P., Mignogna, G., Abdellaoui, A., Hollis, B., Kanai, M., Rajagopal, V.M., Parolo, P.D.B., Baya, N., et al. (2021). Genetic analyses identify widespread sex-differential participation bias. Nat Genet *53*, 663–671. 10.1038/s41588-021-00846-7.

18. Kasimatis, K.R., Abraham, A., Ralph, P.L., Kern, A.D., Capra, J.A., and Phillips, P.C. (2021). Evaluating human autosomal loci for sexually antagonistic viability selection in two large biobanks. Genetics *217*. 10.1093/genetics/iyaa015.

19. Schneider, V.A., Graves-Lindsay, T., Howe, K., Bouk, N., Chen, H.-C., Kitts, P.A., Murphy, T.D., Pruitt, K.D., Thibaud-Nissen, F., Albracht, D., et al. (2017). Evaluation of GRCh38 and de novo haploid genome assemblies demonstrates the enduring quality of the reference assembly. Genome Res *27*, 849–864. 10.1101/gr.213611.116.

20. Morgulis, A., Coulouris, G., Raytselis, Y., Madden, T.L., Agarwala, R., and Schäffer, A.A. (2008). Database indexing for production MegaBLAST searches. Bioinformatics *24*, 1757–1764. 10.1093/bioinformatics/btn322.

21. Zajitschek, S.R., Zajitschek, F., Bonduriansky, R., Brooks, R.C., Cornwell, W., Falster, D.S., Lagisz, M., Mason, J., Senior, A.M., Noble, D.W., et al. (2020). Sexual dimorphism in trait variability and its eco-evolutionary and statistical implications. Elife *9*. 10.7554/eLife.63170.

22. CUERVO, J.J., and MØLLER, A.P. (1999). Phenotypic variation and fluctuating asymmetry in sexually dimorphic feather ornaments in relation to sex and mating system. Biological Journal of the Linnean Society *68*, 505–529. 10.1111/j.1095-8312.1999.tb01186.x.

23. Reinhold, K., and Engqvist, L. (2013). THE VARIABILITY IS IN THE SEX CHROMOSOMES. Evolution (N Y) *67*, 3662–3668. 10.1111/evo.12224.

24. Pomiankowski, A., and Møller, A.P. (1995). A resolution of the lek paradox. Proc R Soc Lond B Biol Sci *260*, 21–29. 10.1098/rspb.1995.0054.

25. Kent, W.J. (2002). BLAT — The BLAST-Like Alignment Tool. Genome Res *12*, 656–664. 10.1101/gr.229202.
